# The (Co)Evolution of Language and Music Under Human Self-Domestication

**DOI:** 10.1007/s12110-023-09447-1

**Published:** 2023-04-25

**Authors:** Antonio Benítez-Burraco, Aleksey Nikolsky

**Affiliations:** 1 Department of Spanish Language, Linguistics and Literary Theory (Linguistics), Faculty of Philology, University of Seville, Seville, Spain; 2 Independent Researcher, Austin, TX USA; 3 Departamento de Lengua Española, Facultad de Filología, Área de Lingüística General, Lingüística y Teoría de la Literatura, Universidad de Sevilla, C/ Palos de la Frontera s/n, Sevilla, 41007 España

**Keywords:** Music evolution, Language evolution, Self-domestication, Cultural niche construction, Music transmission

## Abstract

Together with language, music is perhaps the most distinctive behavioral trait of the human species. Different hypotheses have been proposed to explain why only humans perform music and how this ability might have evolved in our species. In this paper, we advance a new model of music evolution that builds on the self-domestication view of human evolution, according to which the human phenotype is, at least in part, the outcome of a process similar to domestication in other mammals, triggered by the reduction in reactive aggression responses to environmental changes. We specifically argue that self-domestication can account for some of the cognitive changes, and particularly for the behaviors conducive to the complexification of music through a cultural mechanism. We hypothesize four stages in the evolution of music under self-domestication forces: (1) collective protomusic; (2) private, timbre-oriented music; (3) small-group, pitch-oriented music; and (4) collective, tonally organized music. This line of development encompasses the worldwide diversity of music types and genres and parallels what has been hypothesized for languages. Overall, music diversity might have emerged in a gradual fashion under the effects of the enhanced cultural niche construction as shaped by the progressive decrease in reactive (i.e., impulsive, triggered by fear or anger) aggression and the increase in proactive (i.e., premeditated, goal-directed) aggression.

**Supplementary Information:**

The online version contains supplementary material available at 10.1007/s12110-023-09447-1.

Just like language, music constitutes a distinctive behavioral trait of humans. However, current understanding of the role of music in shaping human evolution, as well as the matter of origins of music, remain far from clear—in contrast to what is known about the contribution of language (but see Honing, 2019; Perlovsky, 2017; Schulkin, 2013; Tomlinson, 2015; Wallin et al., 2000, for some hypotheses). At the same time, notable parallels exist between the *structural* and *functional* properties of music and language (see Jackendoff, 2009, for a useful review)—to the extent that some authors have argued in favor of their common evolutionary origins (Brown, 2000; Harvey, 2017; see de Boer & Ravignani, 2021, for a recent critical view). In this paper, we wish to substantiate this view with a new model that heavily builds on current findings and methodologies of evolutionary linguistics. Just like the language types that emerged throughout human history as humans became more tolerant and prosocial, following a steady reduction in reactive aggression (Benítez-Burraco & Progovac, 2020), music acquired diverse typology, complexity, and functionality that accompanied its global spread.

We start this paper by overviewing the commonalities between music and language—one of the very few available sources to establish the evolutionary prehistory of music and language. Next we outline the self-domestication hypothesis of human evolution and explain its benefits for modeling the evolution of music and language. Finally we discuss possible ways for music’s interaction with language in their parallel development.

## Music and Language: Common Evolutionary Roots

Overall, existing hypotheses about the origins of music fall into two general classes. The first one regards music as a by-product of the extended use of some preexisting biologically important capacity, such as vocal signaling, sound imitation, auditory analysis, motor coordination, problem solving, and linguistic communication. The second class of theories claims that music was selected for some evolutionary advantage(s).

Music shares many commonalities with language. Both feature numerous functions, typologies of complexity, and a pronounced evolutionary continuity of the cognitive and communicative abilities of other species. In other words, many characteristic traits of human musical and linguistic communication can be traced in animal communication (see Corballis, 2020; Cowley & Kuhle, 2020; Pereira et al., 2020 for recent views).

We shall list the most important similarities between language and music. First, a number of structural parameters of music—pitch, rhythm, meter, tempo, dynamics, articulation, and timbre—are also exploited by language (Besson & Schön, 2001; Filippi et al., 2019; Heffner & Slevc, 2015; Patel, 2003; Rohrmeier et al., 2015; Slevc, 2012). For example, pitch changes are used to distinguish different words in tonal languages, or different sentence types, as in prosodic intonation (see Nikolsky & Benítez-Burraco, 2022, for ample discussion).

Second, music equals language in many of its common functions:

1. the *expressive* function—especially, conveying emotions (Altenmüller et al., 2013; Cook, 2002; Eerola & Vuoskoski, 2013; Gabrielsson & Juslin, 2003; Johnson-Laird & Oatley, 2010; Juslin, 2005, 2011, 2013; Krumhansl, 2002; Mohn et al., 2010; Nikolsky, 2015a; Panksepp & Trevarthen, 2009; Peretz, 2013; Perlovsky, 2012; Schiavio et al., 2017; Trainor, 2010; van Goethem & Sloboda, 2011),
2. the *phatic* function—in other words, reinforcing interpersonal and social bonding (Boer & Fischer, 2012; Clarke et al., 2015; Clayton, 2016; Cross, 2009; Dunbar, 2012a, b; Harvey, 2017, 2020; Mehr et al., 2021; Savage et al., 2020; Trevarthen, 2002),
3. the *conative* function—in other words, calling to action (Karl & Robinson, 2015; Kühl, 2011; Leman, 2009; Liszkowski et al., 2012; Mehr et al., 2021; Monelle, 2006; Nazaikinsky, 2013; Rodman & Rodman, 2010; Tagg, 2012; Tarasti, 1998; Vuust & Roepstorff, 2008), and
4. the *mnemonic* function—memory conservation (Belfi et al., 2015; Boer & Fischer, 2012; Janata et al., 2007; Levitin, 2019; Nikolsky, 2016b; Tamm, 2019; van Dijck, 2006; Will, 2004).

Third, as with languages, all human cultures have developed different music systems to support important musical behaviors that fulfill specific social and psychological roles. The form-function links between language and music remain quite stable across various cultures and societies. Although during the past 40 years, Western ethnomusicologists have tended to deny the global universality of specific structural patterns of pitch and rhythm organization, their stance seems to be driven by political reasons—mainly, fear of a Eurocentric bias in conducting scientific comparative study of the world’s music traditions (Blacking, 1977; Gourlay, 1984; Hood, 1977; List, 1971, 1984; Nattiez, 2012; Supičič, 1983).^1^ The arguments for the nonexistence of musical universalities are all limited to the absence of specific higher-order combinatorial patterns in certain music cultures rather than to the omnipresence of certain basic principles of music-making (Brown & Jordania, 2013; Fitch, 2017; Grauer, 1996; Justus & Hutsler, 2005; Kolinski, 1978; Lomax, 1977; McAdams, 1989; Nketia, 1984; Savage et al., 2015; Tagg, 2012; Verhoef & Ravignani, 2021).^2^

A number of common elementary “surface-level” music constructs are virtually omnipresent across the globe and rely on the perceptory mechanisms that are already active immediately after birth:

1. In practically every music culture, listeners recognize musical sounds as more pleasant than other types of sounds and are eager to listen to them for a long time, over and over again (Alworth & Buerkle, 2013; Fitch, 2006; Granot, 2017; Hefer et al., 2009; Lots & Stone, 2008; Nieminen et al., 2011; Salimpoor & Zatorre, 2013; Schubert, 2009; Snowdon, 2021; Watanabe, 2008).
2. Listeners distinguish pleasant (consonant) from unpleasant (dissonant)^3^ simultaneous combinations of musical sounds and only vary in judging which specific combinations are considered “consonant” versus “dissonant” (Bidelman & Krishnan, 2009; Brandl, 2008; Cazden, 1959, 1972, 1980; Lots & Stone, 2008; McPherson et al., 2020; Messner, 2006, 2013; Schellenberg & Trehub, 1996; Tenney, 1988; Terhardt, 1974b).
3. Listeners distinguish melodic steps from leaps (Alekseyev, 1986; Bendixen et al., 2015; Bregman, 1994; Larson, 1997; Nazaikinsky, 1977; Rags, 1980; Sievers et al., 2013; Stefanics et al., 2009; Tiulin, 1937; van Noorden, 1975).
4. Listeners distinguish regular integer-ratio rhythms from irregular rhythms (Arom, 2006; Brown & Jordania, 2013; Drake, 1998; Drake & Bertrand, 2001; Fitch, 2012; Fraisse, 1982; Jacoby et al., 2021; Monahan, 1993; Pressing, 1983; Ravignani et al., 2016).
5. Listeners distinguish binary metric groups from ternary (Abecasis et al., 2005; Bergeson & Trehub, 2006; Clayton, 2000; Fraisse, 1982; Iyer, 1998; Jacoby et al., 2021; London, 2004; Monahan, 1993; Potter et al., 2009; Temperley, 2009).
6. Listeners distinguish fast tempi from slow (Baruch & Drake, 1997; Collier & Collier, 2007; Dalla Bella et al., 2001; Ellis, 1992; Fraisse, 1982; Levitin & Cook, 1996; McAuley, 2010; Trainor et al., 2004; van Noorden & Moelants, 1999).
7. Listeners experience music as virtual movement of a certain character, analogous to physical motion, but in imaginary space, formed by the alternation of tension-inducing and relaxation-inducing structures (Fraisse, 1982; Friberg & Sundberg, 1999; Iyer, 1998; Jackendoff & Lerdahl, 2006; Larson, 2012; Larson & McAdams, 2004; Larson & Vanhandel, 2005; Nazaikinsky, 1988; Nikolsky, 2015b; Rothfarb, 1988).
8. Listeners use no more than 12 pitch-classes (most commonly 5–7 of different sizes) and employ logarithmic incrementation to distinguish between them within a pitch-set (Balzano, 1980; Beliayev, 1990; Brown & Jordania, 2013; Gill & Purves, 2009; Honingh & Bod, 2011; Jacoby et al., 2019; Korsakova-Kreyn, 2013; Mazel, 1952; McAdams, 1989; McBride et al., 2022; Sethares, 2005; Shepard, 2010) that possibly shares roots with the linguistic prosody (Fenk-Oczlon, 2017; Kolinsky et al., 2009; Schwartz et al., 2003; Terhardt, 1984).

Numerous experimental studies suggest the existence of universal cross-cultural patterns of musical communication (Argstatter, 2016; Balkwill & Thompson, 1999; Egermann et al., 2015; Fritz et al., 2006, 2009; Juslin & Laukka, 2003; Kwoun, 2009; Laukka et al., 2013; Sievers et al., 2013; Smith & Williams, 1999; Stevens & Byron, 2009; Trehub et al., 1993; Yurdum et al., 2022). This line of research is extremely important in validating claims of Western ethnomusicologists and identifying the common biomusicological foundation that underlies world’s music cultures.

Fourth, newborns show innate predisposition to acquire music no less than language. Hence, there is evidence that fetuses distinguish music from environmental sounds during the last months of gestation, and newborn infants even remember music they were exposed to during gestation (Parncutt, 2016). Acquisition of music occurs implicitly, even in the absence of formal training (Rohrmeier & Rebuschat, 2012). Infants routinely learn multiple music systems just as they learn multiple languages used by their caretakers (Wong et al., 2009). The development of musical skills in childhood seems to proceed in the direction of building new culture-specific skills of identifying culturally important conventional patterns of musical sounds (e.g., “chords” and “keys”), based on the biologically ingrained foundation of synesthetic perception of musical pitch, rhythm, timbre, and dynamics (see the discussion in Nikolsky, 2022). Ontogenetically, this line of development from implicit “natural” (onomatopoeic) and, therefore, cross-cultural and general to explicit “cultural” (convention-based) learning is not that different from linguistic acquisition (Berry et al., 2002; Dasen, 2012; Greenfield et al., 2003; Johnson & White, 2020; Kidd et al., 2018; Monaghan et al., 2014). This emergence of “cultural” forms of musicking from “natural” forms must be responsible for the significant correlation between the geographic distribution of specific genetic variations and specific folk music traditions, as revealed by recent studies (Brown et al., 2013; Le Bomin et al., 2016; Pamjav et al., 2012).

Finally, music perception and production rely on specific brain circuits, the impairment of which leads to distinctive, music-specific damage (i.e., amusia) (Perrone-Capano et al., 2017; Reybrouck et al., 2018; Stewart et al., 2006; Tillmann et al., 2015; Vuust et al., 2022). This substrate shows extensive overlapping with the substrate of language impairments, specifically in syntax processing (Asano, 2022; Brown et al., 2006; Harvey, 2017; Sun et al., 2018; but see Chen et al., 2021, for an opposing view).

Overall, just as one can argue for a human *linguisticality*—the set of capacities that enable humans to learn and use languages in all their diverse forms (after Haspelmath 2020)—one can argue for a human *musicality*, understood as an innate predisposition to perceive and create music, encompassing all the perceptual, cognitive, and behavioral aspects of music. Our contention here is that these parallels can also be extended to the evolutionary domain. Retaining the parallel with language(s) again, in no way should music be regarded as a recent cultural invention.^4^ Musicality must be an ancient capacity that has manifested in different types of music along the long pathway of *Homo sapiens*, reflecting the milestones in the cultural evolution of our species, as well as important cognitive and behavioral changes.

In view of the similarities reviewed above, some scholars (most notably, Brown 2000) have suggested that language and music might share common evolutionary roots. However, as noted by Cross and colleagues (2013), even were this the case, there are several likely scenarios of their emergence: music developing from language (Spencer’s view), language emerging from music (Darwin’s view), or language and music splitting up from a common *musilanguage* (Brown’s view) and afterwards following different, but still related (and perhaps interacting), trajectories (Harvey, 2020). In this paper, we propose a new model of the evolution of music that adheres to the latter possibility.

## What Music Functions Can Tell About the Evolution of Music

Pretty much as for language, one can think of diverse functions for which music might have been selected—and even estimate a timeline for the selection of each type of function. Most of the functions of music mentioned in the previous section can be characterized as “external” to the subject and thus execute some social role: for example, (1) the establishment and consolidation of social bonds within human groups (Dunbar, 2012a, b; Harvey, 2017, 2018; Savage et al., 2020) and (2) the conveyance of credible information to others either for signaling mate quality (e.g., Merker 2000; Miller, 2000) or for coping with progressively complex social conflicts of interest (Mehr et al., 2021).

Nonetheless, an “internal” role for music has been hypothesized as well, such as Perlovsky’s (2017) view of music as a tool for overcoming unpleasant emotions, resulting from our interaction with the environment. Often, “external” functions of music, most notably those related to social bonding, impact the “internal” state of a subject by influencing the stress-response systems or the rewards systems (see Dunbar 2012a, b; Harvey, 2020; Savage et al., 2020, for discussion). Accordingly, it is not an easy task to infer an evolutionary path for these functions.

One promising approach is to cross-examine the codependencies between the most common music functions, based on the music skills required to process those music structures that characterize each of these functions.^5^ Like language, music is structurally determined by the functions it regularly performs (listed below). Once forged, such structures, in turn, start supporting and conserving a function that shaped them.^6^ As a result, these formative functions form complex dependencies whereby one function cannot operate without another function being accessible.^7^ More importantly, some functions build the foundation for others, supporting new modes of interaction with the physical and, particularly, the cultural environment.

In a recent paper (Nikolsky & Benítez-Burraco, 2022) we present a thorough reconstruction of the entire chain of dependencies of the most common formative music functions, tracking them down to the primordial hedonistic function that underlies all others. We identified 14 operational functions in the recent research literature (Bispham, 2018; Boer & Fischer, 2012; Brown, 2005; Clayton, 2016; Dissanayake, 2005; Levitin, 2019; Perlovsky, 2014; Savage et al., 2015; Schäfer et al., 2012; Schäfer & Sedlmeier, 2009; Stefanija, 2007; Trevarthen, 2009; van Goethem & Sloboda, 2011):^8^

- *hedonistic stimulation* (make music or listen to it to experience pleasure),
- *emotional communication* (make or listen to music that expresses one’s current emotional state or characterizes a state of a third party),
- *emotional regulation* (make or listen to a selected type of music to maintain a desired emotional state or to change an undesired one),
- *compliance to norms* (ritualizing one’s behavior and organizing one’s feelings and goals in accordance with some ideal, collective task, or belief),
- *recreation* (entertain an individual or a social group by doing something not totally predictable, such as improvising, exploring a new instrument, or playing some singing/vocalization games),
- *interpersonal bonding* (secure close relations with another individual or a social group by sharing a musical experience with them),
- *coalition status display* (publicly display one’s membership in a specific social group or project and affirm a wish-to-be social identity),
- *physical aid* (support a specific pattern of physical motion in one’s daily work, play, or workout, collective or solitary),
- *learning aid* (stimulate the discovery of new things and help remember important information, as in children’s learning songs),
- *contemplating an event* (evoke the imagery of an important occasion, holiday, season, sporting event, place of interest, landmark, or monument),
- *calling to action* (music signaling, as in military bugle signals or herding calls—i.e., supporting language-like commands—and the creative use of such semiosis to entertain the audience, as in “program music”),
- *conservation of memories* (preserve a valuable memory for an individual and their close family/friends, usually nostalgic, and maintain one’s mental integrity under pressure),
- *self-promotion* (exhibit one’s music faculties to increase confidence, self-esteem, and/or earn respect or show superiority),
- *personal profiting* (earn money and/or fame by making music as a professional occupation).

We cannot dedicate much space to the discussion of these functions here and will only cover those points that directly relate to the evolution of music and language.

**Fig. 1:**
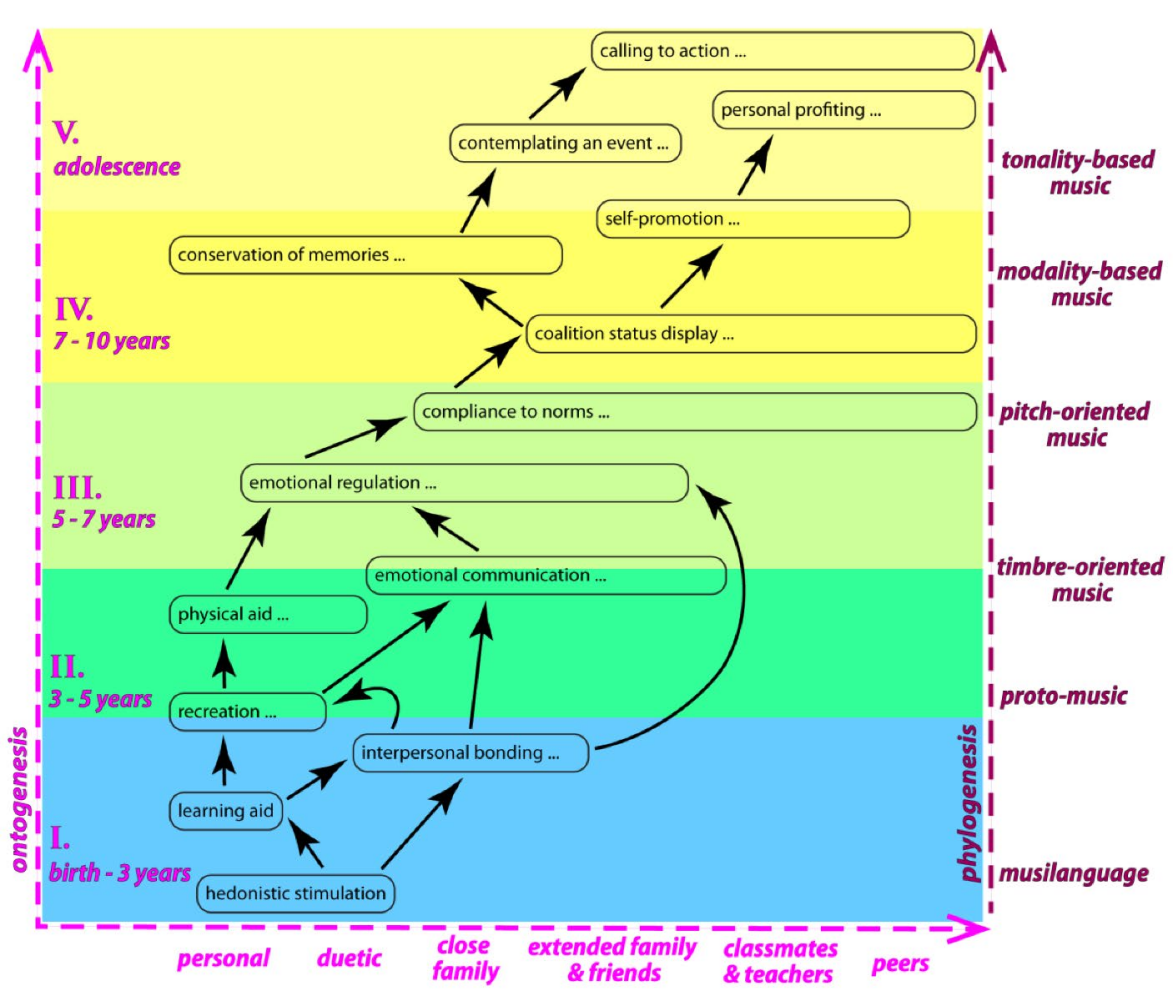
Evolutionary development of operational functions of music. Fourteen operational functions are placed along two axes: temporal (vertical) and social (horizontal). The former (on the left, in pink) reflects the operational dependencies between all functions, which is generally representative of the ontogenetic pattern of acquisition of music skills throughout childhood. On the right (in purple), the corresponding phylogenetic line of development is outlined. The horizontal axis shows the gradual social expansion in the use of functions throughout childhood. The ellipsis after the name of a function indicates that this function keeps developing toward engaging a greater number of participants, the extent of which is reflected by the relative length of the surrounding box after the ellipsis. Black arrows show the derivative relations between functions. A blue rectangle at the bottom encloses functions that are *undifferentiated* from verbal communication and characteristic for the “musilanguage.” A green rectangle marks the functions that are *differentiated* from verbal communication but are not autonomous from it, representative of protomusic and earliest forms of “personal music.” Darker green distinguishes more *biologically* dependent functions from more *culturally* varied ones. A yellow rectangle encloses functions specific to music. Darker yellow distinguishes functions based on *informal*, orally transmitted, and implicit musical grammars from *formally learned*, notation-based, and explicit grammars

Figure 1 summarizes the codependencies that we have established in our 2022 paper (see Nikolsky & Benítez-Burraco 2022 for details). “Hedonistic stimulation” does not depend on any other function and is not only cross-cultural but cross-specific for a number of nonhuman species. Therefore, it is placed at the root. “Personal profiting” and “calling to action” do not support other functions. Therefore, they go to the top. Other functions are distributed in-between according to their dependencies.^9^

Note that the lower-order functions form the succession that fits the pattern of acquisition of musical skills throughout childhood (see the discussion in Nikolsky, 2022). “Hedonistic stimulation” by music seems to be inborn and universal. It supports and enables the acquisition of every other music function. “Learning aid” capitalizes on the capacity of music to bring pleasure, connecting it to the disposition to learn new things and the mnemonic power of music (evident in the earworm phenomenon and the efficacy of music therapy in treating dementia). Multimodal interaction with the mothering figure, whose singing, motherese, touch, movements, and gestures altogether shape this “learning” function, teaches an infant the principles of communication. “Interpersonal bonding” emerges from the ongoing communication with caretakers, usually set by the mother and thereafter expanded to other close relatives. Based on the observed patterns of vocal communication, by the second year of life, infants engage in active musicking—in the form of solitary musical babbling, which introduces the “recreation” function. However, musical babbling remains very similar to verbal babbling. All four of these basic functions are engaged in verbal acquisition too.

Hours of dedicated exercising self-initiated vocalizations, accompanied by spontaneous physical movements, lead to discovery of the expressive capacities of melodic leaps, steps, directionality, dynamics, and, eventually, rhythm and tempo. Infants learn melodic movement as they learn physical movement. Mastering melodic leaps and steps accompanies learning to walk. Thereby, music evolves into the “physical aid” function. Through solitary exploration of singing while moving, playing with toys, drawing, and so on, children discover that certain types of melodic motion suit certain types of physical motion. Specific musical patterns become associated with the affective characteristics of the accompanying locomotion and with the imaginary characters of toys and protagonists of drawings. From this point on, musical expression focuses on “emotional communication,” and verbal expression, on referential communication. However, both keep sharing the same functions: like music, speech conveys emotions, accompanies locomotion in play-games, and entertains (tongue-twisters, nursery rhymes).

Music expression becomes autonomous from speech once children begin using skills they have learned in emotional communication to control their emotional state: avoid negative emotions, bring themselves into a state required by a social situation, and so on. “Emotional regulation” opens doors to “compliance to norms”—children begin learning ritual behaviors for different environmental settings. Music comes handy in organizing “rituals” for collective activities (work songs, play songs, anthems, hymns, theme songs). Since execution of such activities keeps involving a greater number of participants and increasing the distance of musical communication beyond the intimate space (typical for motherese), tonal organization of music starts obtaining pitch orientation (see Nikolsky & Benítez-Burraco, 2022, for details). The emerging pitch patterns become more and more culture-specific—averaging the knowledge and preferences of the growing pool of participants in musicking.

Variety in learned musical rituals enables one to display their “coalition status” to a growing number of people to demonstrate which norms one chooses to abide by. This way music preferences turn into something like a social “identity badge.” This function is exceedingly important among teenagers, laying the ground for another function, very important for adults: “conservation of memories.” Music patronized in youth is usually cherished throughout life and serves to maintain one’s integrity. The latter, in turn, becomes indispensable for “self-promotion.” Raising one’s self-esteem and earning respect through performance and patronization of sophisticated music requires stylistic consistency and adherence to the earlier established values.

“Self-promotion” can evolve into “personal profiting” for those who achieve technical proficiency and artistic integrity in music.^10^ In modern societies, where music schooling supports music notation, reproduction, and wide distribution of music compositions, this option might be quite lucrative—in contrast to folk music cultures, where active musicking constitutes the norm. In turn, notation and formal schooling facilitate accumulation of knowledge and acquisition of basic arranging skills, especially valuable for the evolution of “conservation of memories” into “contemplating an event.” The latter supports the capacity to use music in reference to specific circumstances in their absence from the immediate environment (e.g., contemplating Christmas by listening, playing, or imagining the sound of carols during summer). Building a lexicon of music idioms to refer to many culturally important events (including foreign and exotic) leads to the acquisition of the most advanced musical function—“calling to action.” It supports the capacity to suggest affective states, characters, imagery, and attitudes by choosing and arranging suitable music structures and convincingly rendering them for the audience. This function is almost entirely based on cultural conventions and learning.

## Phylogeny Meets Ontogeny

This entire line of *ontogenetic* musical development finds a close match in *phylogenetic* development—after all, a cultural phenomenon can exist in no other way but through the successful transfer from one generation to another in quantities sufficient for its survival. The success of this transfer is largely determined by the psychophysiological limitations of a learning youngster and the ability of adult experts to cater to that person (see Nikolsky, 2020a, for thorough discussion). Hence, the infantile functions correspond to the musilanguage stage in the evolution of music and language. Both are characterized by the prevalence of personal and duetic settings—epitomized, respectively, in babbling and motherese. Presence of a diverse repertory of relatively well-structured signals, adopted as a standard to convey certain types of information, must have distinguished human musilanguage from animal communication. Longer altriciality and ever-growing capacity to accumulate knowledge must have promoted this diversification. The typical forms of preverbal interactions between infant and caregiver provide at least some idea of how the musilanguage systems might have been put into use by humans before the emergence of modern articulate speech and true human musicality (Harvey, 2017).

Differentiation of musical and verbal acquisition around the age of 3–5 years corresponds to the divergence of protomusic and protolanguage. Their mutual cutoff from the preceding musilanguage probably occurred due to discovery and appreciation of singing and metro-rhythm (likely discovered through the entrained knapping during collective manufacturing of stone tools). This stage can be characterized by crystallization of *protogenres*^11^—forms of musicking developed to accompany collective hunting and repelling of predators or personal caretaking, such as mothering and grooming. The phylogenetic equivalent of the formation of a mother-child “microcosm” (and its further expansion into a “macrocosm” of friends and acquaintances) would be the emergence of a family nucleus and significant reduction of aggression within it among early humans (followed by the expansion of this nucleus). The direct connection between the increase in attachment behaviors, so instrumental for the evolution of language and music, and hormonal effects of the peptide oxytocin on music-related activities has been thoroughly discussed by Harvey (2020).

The next phase of ontogenetic development—learning to express musical emotions and to use them to optimize one’s state—marks the onset of a new phylogenetic stage of “protomusic” turning into “music,” fueled by the emergence of *musical mode*. The latter can be defined as a social convention for combining certain types of musical sounds into sets for expression of a particular topic. Musical modes are inseparable from musical genres: in virtually every folk music culture, each basic genre (e.g., lullaby)^12^ supplies one or a few suitable characteristic musical modes (so that all applications of the same genre sound recognizably the same).

In case of timbre-oriented music, musical modes are timbral—they join not pitch-classes but “timbre-classes” (as in jaw harp music; see Nikolsky et al., 2020). Timbre-matching has been reported in mother-infant communication (Malloch, 2000, 2004), driven by the instinct to adjust one’s vocalizations to those of an interlocutor. The emergence of this capacity likely occurred in the late Paleolithic and marked the birth of timbral music from protomusic.

The next evolutionary advance was the conversion of timbre-classes into pitch-classes and transition from timbre- to pitch-orientation. Ontogenetically, this transition usually occurs at the age of 3–5 years through the practice of “objectivization” of pitch values in music, when salient pitch changes become associated with physical objects, qualities, and events based on the synaesthetic connections between melodic motion and physical motion as observed by children in their environment (see Nikolsky, 2022).^13^ The other important factors contributing to the emergence of pitch orientation are:

1. long chains of folk-style person-to-person transmission (see Nikolsky & Benítez-Burraco, 2022, Chap. 5),
2. spread of collective singing with the accompaniment of rhythmic and melodic musical instruments (Morley, 2013),
3. concentration of people in a confined space of caves that became the preferred form of shelter toward the end of the Paleolithic (the reverberation converts melodic intervals into harmonic intervals by prolonging the “tails” of preceding melodic tones; Nikolsky & Benítez-Burraco, 2022),
4. musicking at distances where listeners cannot discriminate between different timbre-classes, especially if the distance changes during the same session of musical communication (as in herding; see Nikolsky, 2020b).

The fifth phase of musical ontogenesis corresponds to the evolutionary stage when musical keys emerged from musical modes (first documented in ancient Greece; see Nikolsky, 2016c). The extensive use of keys within a particular music culture led to the formation of “tonality,” which came to replace “modality” (Nikolsky & Benítez-Burraco, 2022). In short, this stage is characterized by the adoption of standardized tuning, as defined by the practice of tuning musical instruments most important for a given musical culture. Musical keys “canonize” specific sets of pitch-classes, convenient for playing on the preferred musical instruments. Such sets become adopted for other musical instruments and vocals within the same music culture through the practice of mixed ensemble performance. With the advance of ensemble music and rise of formal music theory, a culture establishes an assortment of keys for conventional forms of expression across all the important genres, generating a “tonality”—a system of keys (i.e., a *set of sets* of pitch-classes). Western classical tonality constitutes just one particular case of tonality. Indian *raga*, Arabic *maqam*, Persian *dastgah*, or Chinese *yuye* each implement “tonality” in their own way, according to their cultural values.

If the musilanguage and protomusic stages are characterized by cross-cultural uniformity, since they rely mostly on the innate forms of encoding information into auditory signals (what we call *anthropophonic* and *onomatopoeic* intonation types), the tonality stage exhibits maximal cultural diversity and minimal universality of the musical expressive means. That is, the task of comprehending music created within a tonality system absolutely requires a listener to learn the conventions of the corresponding music culture. In contrast, comprehension of the earliest forms of tonal organization of music can rely on the biomusicological universalities and synesthetic environmental associations. The challenge of conducting a comparative study of music (synchronic and diachronic) is that each musical function, once established, remains accessible, supporting the higher-order functions, while becoming adjusted to the broader user-base. Functions, as well as music genres and traditions that rely on such genres, do *not* become replaced by newer functions, genres, and traditions, but accumulate, disappearing only after a prolonged absence of any use.^14^

For example, the foundation for “physical aid” function is prepared by the mother moving the infant’s limbs in concert with her motherese talking and singing. Embodied in this way, sound patterns are further explored by a child during sessions of solitary babbling, accompanied with spontaneous self-induced locomotion. The discovered correspondences between melodic and physical motion are further explored in singing that accompanies solitary playing with dolls, toys, and in drawing, where each character receives a dedicated musical pattern. As the child grows up, such games start involving playmates and including nursery rhymes, ditties, and popular songs, rearranged for each instance of application. As children learn the assortment of patterns of various musical movements, they can participate in work-songs and other music-based activities together with adults (which is exceedingly common in traditional societies). In modern urban culture, teenage children rapidly advance to the stage of mass consumption of music—they switch from active performance typical for earlier childhood to passive listening and learn to select music for background listening while doing something (e.g., during physical exercises). This way, the initially *personal* use of melodic motion ends up expanding to involve up to thousands of participants (e.g., a session of rhythmic gymnastics streamed over the internet) as the “physical aid” function passes through developmental rounds with a broadening user-base. Similar development must have taken place in the cultural evolution of music as human societies grew in size and complexity, and music was put into serving a greater number of users.

The most important take from the variability of musical functions and their cumulative nature is that any analysis and comparison of music should involve the entirety of relevant musical functions, their structural implementation, and the quantity of their users.

## The Formative Power of Cultural Transmission on Music and Language

To add a final piece to the evolutionary puzzle, we need to point out that cultural transmission per se exerts a formative power over music structures—just as verbal structures are shaped by transmission chains. Thus, Lumaca and Baggio (2017) experimentally demonstrated how transmission altered pitch and rhythm aspects of the transmitted pattern, resulting in *diatonization* of the initial model—in other words, *chromatic* semitones being systematically replaced by *diatonic* whole tones, thereby increasing music’s compliance to conventional keys. The formative power of transmission goes as far as to transform *ekmelic* intonations—gradual changes in pitch and indefinite pitch values (like pitch contours of spoken sentences)—into *emmelic* intonations (incremental changes in pitch with definite pitch values) at the end of a transmission chain (Verhoef, 2012; Verhoef et al., 2014).

Discretization and diatonization seem to occur because the transmitter tends to complicate a specific pattern in an attempt to increase its expressivity, whereas the receiver tends to simplify it for the sake of easier learning (Kirby et al., 2015). This trade-off eventually results in the increased compressibility of the encoding and the regularization of the variables. The longer the transmission chain, the stronger the effect. Iterated learning generates natural selection for optimal acoustic distinctiveness, supporting the transformation of non-combinatorial signals into combinatorial signals (Zuidema & de Boer, 2009). The same process is at work in linguistic and musical transmissions: each receiver intuitively strives to minimize entropy while learning a structure, which promotes compression of information and the emergence of compression regularities, thereby generating grammars (Tamariz & Kirby, 2016). Here, yet another peculiarity of transmission comes into play—each new learner tends to bring into uniformity those structures that just slightly differ (Smith & Wonnacott, 2010). This leads to crystallization of grammatical rules.

A number of scholars have denied that music has grammar, meaning, and compositionality. The reasons for this are numerous:

- confusion over the typology of music functions and uses,
- disregard for music structures and analysis of music form, common among Western ethnomusicologists,
- absence of a general definition of music and disinterest in coining it,
- demise of comparative ethnomusicology in the West after WWII for political reasons, and
- a pronounced Eurocentric bias among many Western cognitive scientists and developmental psychologists who hold Western classical music as the universal or ultimate model of tonal organization.

Nonetheless, what tells music apart from other auditory phenomena, we believe, is music’s overall orientation toward putting the listener in a specific premediated emotional state and keeping them in that state for an extended period of time—and doing this repeatedly, so the same type of sonic material becomes associated with a specific type of semantic content by means of public convention (see Nikolsky 2015a, 2020b; Nikolsky & Benítez-Burraco, 2022).^15^ We realize that the idea of tying the concept of music to emotion appears unattractive to many scholars with a background in classical music composition, performance, and music history, ever since Stravinsky and the post-WWII avant-garde won critical acclaim in Western academia and among prestigious cultural philanthropic organizations.^16^ However, any attempt to tweak the general definition of music in order to incorporate the latest short-lived (just a century long) development of just one music tradition (albeit a very important one) is methodologically wrong (generalizing on a sample size of one). We cannot name a single non-Western musical tradition that abstains from using musical emotions and musical genres (which usually serve to assign affective qualia to specific music structures, generating convention-based semiosis in music).^17^

Morphologically, music closely follows language in employing both combinatoriality and compositionality, although, as noted above, there is some controversy as to whether music syntax is processed in the same cortical regions as language syntax. Music combines many *meaningless* elementary units—pitch-, rhythm- and timbre-classes, metric beats, and voices in texture—to generate *meaningful* morpho-syntactic units, such as motifs, chords, rhythmic figures, metric groups, and textural components (e.g., accompaniment, counter-melody, pedal tone) that carry certain semantic values (sighing motif, sad chord, bouncing rhythmic figure, leisurely swaying ternary meter, stiffening pedal tone, etc.). These morpho-syntactic units are conjoined according to a set of rules that distinguish each musical tradition, enabling listeners to identify a tradition by ear (Nazaikinsky, 1982). For instance, in Gregorian plainchant, melodic leaps, regular meters, the so-called dotted (or “punctured”) rhythms, chords, and chromatic alterations are to be avoided altogether (Ferreira, 1997), whereas in Western military march music they are encouraged (Monelle, 2006). Mastering such traditions requires apprenticeship so a layperson can learn their compositional principles.

Historic ethnomusicology testifies to the fundamentality of compositional organization of music. Western, Arabic, Persian, Indian, and Chinese classical music traditions each feature hundreds if not thousands of treatises on music composition.^18^ Western compositional music theory is rooted in the ancient Greek theory of rhetoric, understood as the craft and science of bringing the audience into a specific emotional disposition (Bartel, 1997; Bonds, 1991; Harrison, 1990; Kallberg, 1988; Keller, 1973; Mabbett, 1990; Meier, 1990; Vickers, 1984; Zakharova, 1983).^19^ The musical implementation of rhetoric occurred initially through the liturgic practice of composing sermons and supporting the verses required for liturgy with music (Murphy, 1981), but by the eighteenth century the theory of musical rhetoric firmly held its ground in purely instrumental and secular forms of music (see Mattheson & Harriss, 1981). Other musical cultures featured their own pathways of developing musical rhetoric (see Dorchak 2016; López-Cano, 2020; Powers, 1980; Rink, 1989; Smith, 1971; Theodosopoulou, 2019), including such a recent development as composing music for advertising (Scott, 1990).

Chain transmissions inherently introduce and magnify cultural biases in music structures and combinatorial and compositional rules since different cultures favor different structural features in response to culturally dependent factors, such as popularity and social prestige. The same applies to the domain of speech (Verhoef et al., 2014). More generally, experiments involving artificial languages suggest that the cultural transmission of linguistic structures promotes compressible regularities, combinatorial rules, and compositionality (Kirby et al., 2015; Tamariz & Kirby, 2016). The analysis of sign languages spontaneously developed by isolated deaf populations also suggests that some basic properties of language (such as duality of pattern) are lacking at the beginning of transmission and emerge gradually as a result of increased interactions between signers (Dachkovsky et al., 2018; Sandler et al., 2005).

In the case of music, it is more difficult to identify “idiomatic” structures (i.e., music lexicons of specific music-user communities) and combinatorial rules (i.e., conventional music grammars). The reason for this might be the growing prevalence of “tree-like” transmission—in other words, chain-like passing of a music work from a person to a group (Nettl, 2005).^20^ “Tree-like” transmission tends to replace folk-style “linear,” person-to-person transmission as notation, formal music theory, and professional forms of public performance begin to obtain a greater share in a musical culture. Notation and theory substantially aid learning, thereby reducing the formative power of simplification in learning on the part of the listeners throughout the transmission chain.

The presence of an audience, in turn, incentivizes performers to intuitively amplify their expression in order to increase rhetorical control over the listeners. As a result, the innovation rate in exploring newer expressive means grows—structural patterns are modified more at each new act of transmission. Subsequently, the diversity of the emerging variants increases since each of the multiple listeners inevitably introduces slight variations in the learned music when they pass it on to new listeners. The compound effect of the tree-like transmission greatly exceeds that of linear transmission. Prevalence of linear transmission makes music cultures that remain primarily “personal” (e.g., Nenets or Nganasan) in their music usage to stand out as amazingly conservative in comparison to music cultures that primarily employ collective forms of performance and listening. The larger the number of the ensemble performers typical for a given tradition (e.g., orchestral music) and the size of its audience (e.g., concert hall, radio), the higher the innovation rate (Alekseyev, 1976, 1986, 1988). Naturally, the greater the discrepancy between synchronic and diachronic invariants of the same musical structure, the vaguer its structural and semantic characteristics and the weaker the combinatorial rules of its use. Language does not have this problem because, in everyday use, “person-to-person” distribution remains prevalent over “person-to-group” (for the discussion of harmonization versus individualization, see Harvey, 2017).

Music is more oriented to the expression, transmission, and *prolonged* experience of emotions, whereas language is more optimized for delivering *prompt* referential information. Therefore, oral verbal encoding is designed for quick peer-to-peer streaming, where information has to be constantly chunked by parsing the stream of sounds, identifying words in it, retrieving their meanings, interpreting phrases, and constructing the meaning in a cumulative way. All of this relies on clarity of phonemic and morphological contrasts, while prioritizing the processing speed and robust error-correction.

Conversely, music prioritizes continuity and *homogeneity* of the sounds within the same musical phrase. Music is designed to elicit particular affective states in the listener, allowing them to immerse themselves in the music and fully engage with the experience of those states. This requirement causes music to:

- slow down music’s transmission rates, giving music a meditative appearance,
- cause music to simultaneously engage multiple aspects of expression, each with its own proprietary “idiomatic” patterns (rhythmic, metric, melodic, harmonic, etc.), and
- ground music to iconic semiosis and synesthetic correspondences between the musical meaning and the acoustic attributes of music sounds.

This distinction between music and language is far from being clear-cut. Language also conveys emotional contents and is partially iconic—especially poetic speech, in which iconicity facilitates word learning and communication while systematicity facilitates category learning. Linguistic arbitrariness, iconicity, and systematicity interact in complex ways under the effects of cultural selection to reshape not only a language’s vocabulary but also its grammar, promoting compositionality and regularity (Dingemanse et al., 2015). Nevertheless, the differences between musical and linguistic oral transmissions are sufficient to make music functions form operational relations quite different from language functions. Notably, music functions rely on each other to such an extent that higher-order functions can hardly be fully operational without lower-order functions being effectively engaged.

Subsequently, the study of the evolution of music requires the consideration of all music functions in their systemic relations. Most disagreements between extant theories of the evolution of music seem to originate from the limitation of study to only a few functions, specific to the earliest or latest stages of evolutionary development, while ignoring the other functions. Moreover, both biological and cultural factors need to be considered on par and in their interaction.

## Human Self-Domestication and Language Evolution

In the next two sections we present a model of music evolution to account for musical functions and for biological and cultural factors formative for music structure and function. Our model is based on a recent account of human evolution, namely, the hypothesis of “human self-domestication” (HSD), which has been successfully applied to the characterization of the evolution of language in our species (Benítez-Burraco & Progovac, 2020; Thomas & Kirby, 2018). Because of the parallels between music and language discussed above, we expect this evolutionary model to be applicable to music.

The HSD hypothesis supports the view that the human phenotype is, to a large extent, the outcome of an evolutionary process similar to that of animal domestication. In nonhuman mammals, domestication initially involved selection for tameness and resulted in a set of distinctive traits—physical, cognitive, and behavioral—that usually co-occurred, forming the *domestication syndrome* (Wilkins et al., 2014; see Lord et al., 2020, and Sanchez-Villagra et al., 2019, for critical views). This might be due to the fact that tameness reduces the input to the neural crest, an embryonic structure that supports the ontogenetic development of numerous body parts (Wilkins et al., 2014; see Lord et al., 2020; Sánchez-Villagra et al., 2016). The HSD hypothesis builds on the findings of many domestication traits in humans, including smaller skulls/brains (compared with archaic humans), reduced hair, neotenic features (e.g., extended childhood and increased playing behavior), and, particularly, reduced levels of reactive aggression (Fukase et al., 2015; Leach, 2003; Plavcan, 2012; Shea, 1989; Somel et al., 2009; Stringer, 2016; Zollikofer & Ponce de León, 2010).

Diverse factors have been hypothesized to trigger HSD, including the rise of co-parenting, the advent of community living, changes in our foraging ecology, climate deterioration, and the colonization of new environments (Brooks & Yamamoto, 2021; Pisor & Surbeck, 2019; Spikins et al., 2021). All in all, these factors might have promoted a selection toward less reactive and more prosocial behaviors, thereby instilling in humans a constellation of physical, behavioral, and cognitive changes characteristic of domestication. Many human-specific traits, such as our enhanced social cognition, increased cooperation, and finally, advanced technology and sophisticated culture, are the products of domesticate-like adaptation (see Hare, 2017, for an overview). This collective cooperativity that extends beyond the familial gene pool does not necessarily equate to domestication, but it quite closely resembles its principal traits.

It seems to us that HSD presents a useful evolutionary framework for linguistic studies, especially for capturing those aspects of languages that are thought to emerge through a cultural mechanism. It is worth remembering that the earliest hominids, who had high levels of reactive aggression, practiced musilanguage rather than “language” and must have cultivated signals similar to animal communication. The latter simply could not support the “duality of patterning” (Hockett, 1960) and combinatoriality. Therefore, the “linguistic” component in musilanguage is harder to see than the “musical” component, although there is evidence that animal communication uses referential as well as motivational information, each coded differently (Manser, 2010). Indeed, animal communication comes much closer to human music than to human language due to its dedication to showing the signaller’s affective state (Fitch, 2006). There is neurophysiological evidence that “full language” must have crystallized later than “full music” because the acoustic characteristics of primate vocalizations are mainly determined by music-like features that serve as the foundation of verbal acquisition for human infants (Koelsch, 2009).

However, concluding from this that language evolved from music, as argued by Fitch (2010), seems a far stretch. The principal arguments against this scenario were summarized by Tallerman (2013):

1. Phonological systems do not evolve in isolation from semantics, as if they were “bare vocal sounds.” Consonants and vowels are linguistic entities, and phonological expansion derives from a growing vocabulary of words—not the other way around. It is the developing lexical system that brings to life phonological gestures (de Boer & Zuidema, 2010; Lindblom, 1998; Studdert-Kennedy, 2011; Zuidema & de Boer, 2009).
2. Despite greater similarity to animal vocalizations than human language, human song remains fundamentally different from animal vocalization. Animal-learned vocalizations lack transposability of intentions (i.e., repeated use of the same signal in different circumstances) and abstraction of the representation of an affective state, which are the landmarks of musical emotions. A single animal call is the basic unit of animal communication—produced instinctively in response to the actual stimulus present in the environment (Zuberbühler, 2017). And animal-learned vocalizations are limited to display of fitness (Naguib & Riebel, 2014), are season- and gender-specific (Slater, 2011), and relate to mating or territory-defending situations (Slater, 2001)—unlike human music.
3. Animal learned vocalizations (some ethologists and researchers of animal communication call them “animal songs”) have a critical period of acquisition, are learned holistically, and take months before an animal can deliver them (Hurford, 2012). In contrast, humans can learn songs at any life-stage, doing it incrementally and rather quickly. Evidently, human song-learning engages very different neuro-physiological mechanisms and constitutes not an extension but a *parallel* evolutionary development to animal song—as Fitch himself recognizes (Fitch, 2010:184).^21^
4. Finally, it is hard to explain how and why music-like aspects of hominin vocalizations would have reduced their musicality and given rise to consonants that are fundamentally “unmusical” and notably absent in animal communication (Kolinsky et al., 2009). The musicality of speech comes from prosody, and prosody comes from joining words into phrases. Musical phrases have nothing in common with linguistic phrases other than the misleading term “phrase” (Benjamin et al., 2015)—linguistic phrases are built around words and their categorical relations, whereas musical phrases are determined primarily by the breathing rate that characterizes different emotional states (greater excitement transpires in shorter phrases) and general release of tension (harmonic and melodic) toward the end of a phrase, which accompanies expiration (Alekseyev 1976).^22^

As we see it, the evolutionary continuity of animal communication and human music is superficial—human song and animal song constitute independent developments—and there is no reason to trace the origins of language from music. Under closer scrutiny, animal communication combines the semiotic characteristics of both human music *and* language (Manser, 2010):

- Animals use *referential* calls (i.e., they refer to specific attributes of the eliciting external stimuli to enable the receivers of these signals to react to these external stimuli) when encountering predators, discovering a food resource, and in agonistic social interactions,
- Animals use *motivational* calls (i.e., calls that display the emotional state of a caller, so the receivers react to this emotional state) in all other situations. Ontogenetically, acquisition of motivational calls precedes acquisition of referential calls and appears to be simpler in structure.

Musilanguage must have just inherited referential and motivational specialization from animal communication and advanced it to the next evolutionary stage—building the repertories of calls of both types and introducing some transposability of their use. In this process, each type obtained a set of characteristic structural features that allowed listeners to distinguish both types upon hearing them. Motivational calls probably resembled the repertory of infantile vocalizations during the first few months of life, categorized into negative cries of various sorts and positive cooing—all characterized by prolonged use, as with music (typically, as long as the emotional state lasts).

Referential musilanguage calls likely resembled the earliest attempts of an infant to point to specific things in a dialogic communication with a caretaker with the aid of gestures—shorter and more of turn-taking than the “monologic” motivational vocalizations. Such a “wordless” linguistic component is what Brown outlined in his 2017 amendment of the musilanguage theory with his new “prosodic scaffold” model (Brown, 2017). According to it, musilanguage conveyed primarily affect-related information in two principal ways:

1. through “affective prosody” (music-like) by means of anatomically available and innate impulse-driven modulations of pitch, loudness, and tempo—which remain global and holistic for the entirety of a call;
2. through “intonational prosody” (speech-like) by filling a prosodic scaffold with phoneme-like deictic utterances—employing both global and local mechanisms for conveying linguistic modality (e.g., question versus statement) and emphasis (stress, prominence, focus).

The speech-like way must have evolved from the music-like way through an ongoing adaptation of the reflex-based vocalizations in response to the most common environmental situations. Such vocalizations were probably reshaped by their chain transmission and natural selection for the most effective patterns of communication under the pressure of time—in other words, to successfully deliver signals as soon as possible, enabling live updates on critical changes in the environment. The demand of urgency probably pushed “intonational prosody” toward language, in contrast to “affective prosody,” focused on the caller’s expression rather than the task of keeping listeners up-to-date. Supported with hominin’s capacity for accumulation of knowledge, the newly forged intonational patterns were memorized and preserved (in contrast to animal communication), leading to the invention of consonants, formation of syllables, and eventual adoption of basic conventional words for the most common objects.^23^

With regard to HSD, musilanguage, protomusic, and protolanguage all fall out of its scope, since currently available data do not indicate the presence of a domesticated phenotype among extinct hominins, and the data coming from developmental psychology and ethnomusicology is applicable to *Homo sapiens* only. Extrapolating our conclusions on the factors at play (see Nikolsky & Benítez-Burraco, 2022, for details), it is plausible to expect that hominins who practiced protomusic and protolanguage, perhaps even musilanguage, had lower levels of reactive aggression than nonhuman primates. Some traits established for *Homo erectus* might be interpreted as promoting cooperative behaviors between closely related partners: hunting and gathering in groups, caring for injured and sick group members (Leroy et al., 2011), need for helpers during delivery due to large cranial size, caretaking assistance due to longer altriciality (Boaz & Ciochon, 2004), and migration to colder climates, where hardship of survival was likely to encourage mutual support in such activities as communally planned big game hunting, maintaining fire, and making clothes and huts—all suggestive of some form of communication between the participants (Mania & Mania, 2004). However, such arguments remain speculative until more conclusive archaeological evidence is uncovered.

For humans, HSD can account for the evolution of abilities and behaviors that enable the cumulative growth of linguistic complexity through already ongoing, multigenerational learning and use. This involves language teaching and practicing, promoted by a more prosocial and neotenic phenotype. In a series of related papers, Progovac and Benítez-Burraco (2019; Benítez-Burraco & Progovac, 2020, 2021) have developed a detailed model of how HSD might have contributed to the evolution of language (and of languages). At the time of the emergence of early humans, reactive aggression was still high, and consequently, communication through language must have been limited to single-word commands, threats, and exclamations, mostly aimed at conveying emotions. Patient and cooperative turn-taking, using long utterances, and conveying referential meanings, frequently observed in present-day interactions, were simply unattainable back then.

Increasing HSD supported stronger in-group networks, involving more diverse, frequent, and prolonged contacts between their members. Cooperative turn-taking must have become more common and elaborated, enabling the development of linguistic structures via cultural transmission. It is plausible to expect that single-word utterances were replaced by rudimentary two-slot grammars made of nouns and verbs to express predications. These earliest grammars might have been primarily used for creating colorful derogatory expressions (since emotional reactivity was still quite high), contributing to further increase in HSD, as these derogatory utterances helped replace physical reactive aggression with less-harmful verbal aggression.

The main reason for the positive feedback loop between reactive aggression and grammar is the functional connection and partial overlap of the brain mechanisms that support combinatoriality and control of reactive aggressivity. To give just one example, in learned aggressive actions (a form of controlled aggression), the prefrontal cortex regulates the activity of the hypothalamus (a component of the “core aggression circuit”) and the striatum (part of the “learned aggression circuit”; Lischinsky & Lin, 2020). But the striatum plays a key role in grammar processing as part of the procedural memory and, more generally, of the cortico-subcortical networks responsible for hierarchical processing (Teichmann et al., 2015). Evidence of this functional connection/partial overlap is the concurrence of the difficulties in processing structural aspects of language with the aggressive outbursts in clinical conditions, caused by striatal dysfunction (Rosenblatt & Leroi, 2000; Savage, 1997; Zgaljardic et al., 2003). Accordingly, from an evolutionary point of view, one can expect that reduced reactive aggression, resulting from increased HSD, demanded additional control of subcortical structures by the cortex, which also promoted cross-modality. In other words, the ability to combine information from different cognitive domains was pivotal for merging linguistic items (see Benítez-Burraco & Progovac, 2021, for a more detailed discussion).

Once HSD reached its peak at the end of the Upper Paleolithic (Cieri et al., 2014), behaviors conducive to the advance in linguistic complexity via cultural mechanisms proliferated: more frequent and diverse social contacts, longer learning periods, more frequent practicing, and so on. Such changes likely put in place the first hierarchical grammars that expressed transitivity. Languages with such grammars are called *esoteric*. These languages typically exhibit larger sound inventories and complex phonotactics, opaque morphologies (with more irregularities and morpho-phonological constraints), limited semantic transparency (abundant idioms and idiosyncratic speech), reduced compositionality, and less sophisticated syntactic devices. These features are common for languages spoken by isolated human groups, living in small, close-knit communities with high proportions of native speakers—a rough proxy for languages spoken by present-day hunter-gatherer societies.

The transition from the Upper Paleolithic to the Neolithic was accompanied by cardinal changes in social organization as a result of steady demographic growth and climatic changes. Growing social interactions brought to life extensive social networks, promoting trading and mating, while also unleashing intergroup hostilities over competition for limited natural resources. The necessity to regulate conflicts, convey decontextualized meanings, and exchange technological know-how with unrelated individuals favored the emergence of another type of language—*exoteric*. These languages typically feature expanded vocabularies and increased syntactic complexity (including greater reliance on recursion), as well as greater compositionality and enhanced semantic transparency—all advanced at the cost of simpler phonological inventories and sound combinations, and more regular morphologies. A proxy of such languages are those spoken by present-day agriculturalist societies, particularly state-governed autochthonous ones. Since these languages are also suitable for conscious planning, establishing alliances, conducting warfare, and, ultimately, supporting the emergence of cultural institutions related to war and peace, their emergence can be linked to the advent of *proactive* aggression that became more widespread during the transition from the Neolithic to the rise of first civilizations.

Our model of evolution of human languages under the effects of HSD can also explain modern pragmatics and linguistic modes of interaction. A reduction in reactive aggression is beneficial for cognitive and behavioral changes necessary for the emergence of rules of turn-taking and complex inferential abilities, both of which are cornerstones of our conversational abilities. On the cognitive side, the expansion of pair-bonding to nonreproductive relationships marked a crucial achievement in social organization. The potentiating of cross-modal thinking, instrumental for linguistic chunking, enabled conventions of figurative uses of language (e.g., metaphors and metonyms) and pragmatic inferencing. On the behavioral side, increased HSD favored prolonged face-to-face interactions, long-term cooperation, and consideration for others’ needs. Overall, these cognitive and behavioral changes enabled communication of more complex meanings by indirect means (see Benítez-Burraco et al., 2021 for a detailed view).

In general, this HSD model ties the evolution of language to changes in aggression management, both reactive and proactive, ultimately connecting specific linguistic structural features with the HSD-related behavioral and cognitive changes, based on their shared neurobiological substrate. At the same time, this model establishes a strong continuity between communication and cognitive abilities exhibited by other species, while also supporting cultural niche construction, cultural evolution, and gene-culture coevolution as key factors that accounted for the exclusiveness of language to human communication. Our contention here is that the same model can be also applied to human musicality, music types, and functions of music—not only because of the common origins of music and language, but mostly because of the common effects of changing levels of reactive and proactive aggression.

## Human Self-Domestication and the Evolution of Music

As noted, we find our HSD model of coevolution more parsimonious than those accounts that hypothesize different rationales and mechanisms for the evolution of music and of language. Our approach reconciles hypotheses about music evolution that have been presented as irreconcilable, such as the “social bonding hypothesis” (Savage et al., 2020) and “the credible signal hypothesis” (Mehr et al., 2021). Moreover, this model explains better than other models how different types of music diachronically emerged through a cultural mechanism—which was previously examinable mostly through memetic approaches (see Jan, 2018). Overall, we hypothesize that the gradual changes in the subtle balance between reactive and proactive aggression could help us understand the steady complexification of music, the emergence of its new functions, and the transformation of the old ones, as well as the past and present distribution of musical types and genres as reported by ethnomusicologists.

**Fig. 2:**
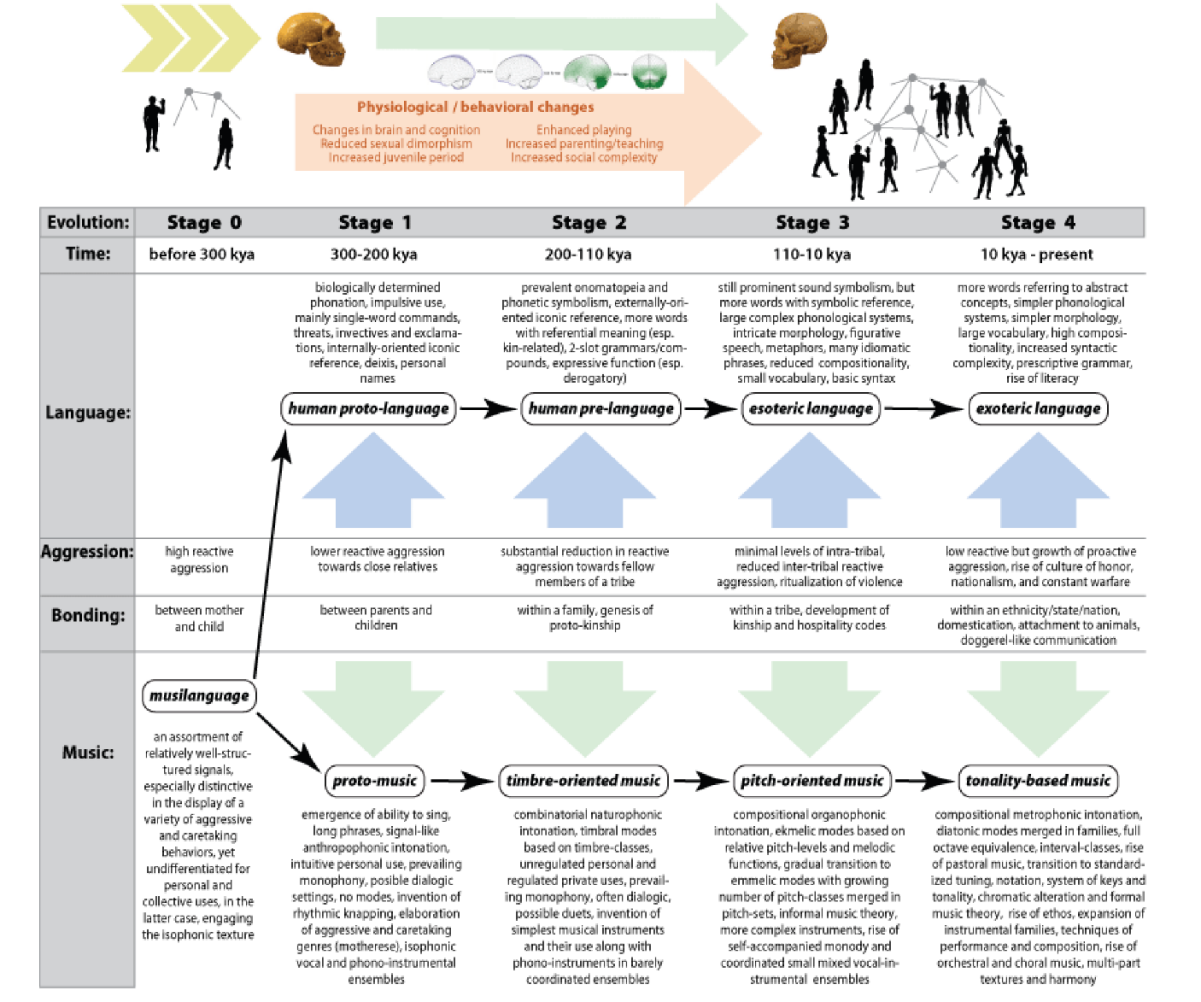
The timeline of the coevolution of music and language. The figure reflects the evolution of types of music vis-à-vis the evolution of types of languages in regard to the changes in human socialization patterns under the effects of increased HSD (reproduced from Nikolsky & Benítez-Burraco, 2022, Fig. 7)

Our model is summarized in Fig. 2, which presents music evolution vis-à-vis language evolution to highlight their common origins and their parallel evolutionary pathways under the effects of HSD, changes in paleoclimatic conditions, demographic changes, and relevant cognitive and behavioral innovations. We support the view that the evolution of music systems and languages can be conceived as two different products of the same biological/cultural processes, heavily influenced by the increased feedback loop between the reduction of reactive aggression and the sophistication of language and music structures and uses.

In brief, once a musilanguage emerged from the building blocks rooted in animal communication, cognition, and behavior, protomusic started to diverge from protolanguage, later evolving into timbre-based music, and thereafter, into pitch-based music, ultimately generating collective forms of music that can be found in many present-day societies. Still, these stages should not be viewed as a clear-cut “monolithic” order of things. Since environmental and social conditions instrumental for HSD are always in the process of transformation, HSD levels are prone to vary from one place to another, and from one human group to another (see, e.g., Gleeson & Kushnick, 2018, for sexual dimorphism under the HSD effects). Therefore, we expect significant historic and geographic overlaps between different evolutionary musical types globally. As the available ethnomusicological data suggest, the schemes of tonal organization that characterize different stages of evolution of musical structures tend to build on each other, retaining the previous formations. Even in music cultures of modern Western countries that are based on full-fledged tonality (the conventional key system of Western classical music), it is often possible to identify traces of the older methods of tonal organization (musical modes, including those that feature fewer than seven pitch-classes—i.e., five strata of tonal organization in traditional Lithuanian music; see Leisiö, 2002). Traces of earlier music usually survive in specific folk genres—most commonly, within the venerated epic and religious traditions.

It would be unrealistic to expect that each stage in our model started at the same time worldwide. This is in line with current evidence of modern human behavior having appeared in different regions at different points of time (Ashton & Davis, 2021). We reserve the possibility that other close hominins, particularly Neanderthals and Denisovans, will fit in the first stages of our model, if evidence of their human-like management of reactive aggression emerges. Below, we provide a more detailed description of our model.

## Summary of Our Four-Stage Model

Before the advent of our species, roughly 300,000 years ago,^24^ a musilanguage stage can be hypothesized for the hominin clade. The likely distinction between animal communication and this pre-human musilanguage was the presence of conventional acoustic forms of expression for conveying common emotional and deictic information between the members of the same social group (loud collective signals to fend off dangerous predators, individual grunting patterns to accompany caretaking activities, etc.). Such signals were probably not coordinated in pitch and time between multiple participants, featuring a jumbled “isophonic” texture (Nikolsky, 2018)—very much like the howling of a wolf pack. But unlike animal communication, musilanguage signals can be hypothesized to address specific group members, to vary in sonic patterns based on application, and to be passed on from one generation to another (see Nikolsky, 2020a). A communication system capable of enhancing sociality and altruistic behavior is critical to the promotion of cooperation at times of environmental stresses, so frequent throughout the Paleolithic. It is plausible that waves of mass hominin migration from Africa were enabled by the prosocial influence of the musical component in a musilanguage system.

Stage 1 in our model (*protomusic*) starts with the emergence of archaic, anatomically modern humans (AMHs) endowed with cognitive innovations—particularly, with a new neuronal workspace that entailed greater connectivity between distant brain regions and could overcome the limits of core knowledge systems, supporting basic forms of cross-modal thinking (Boeckx & Benítez-Burraco, 2014). Two innovations distinguish protomusic from both musilanguage and protolanguage. The first—the emergence of singing—was a likely outcome of an attempt to maximize the intensity of phonation in distant calls (Maclarnon & Hewitt, 2004) and in collective vocalizations designed to scare off predators or to ambush prey (Jordania, 2011, 2017). The second innovation—the accidentally discovered sounds of flintknapping—probably gave birth to the world’s earliest musical instrument, a pair of rocks hit or rubbed against each other in the manner of modern claves or guiro (Montagu, 2004). Rhythmic knapping is known to be a natural by-product of entrainment during collective manufacturing of stone tools (Zubrow & Blake, 2006). The latter was definitely used in prehistoric times (Boivin et al., 2007) and still survives in aboriginal societies in performance rituals, where it is ascribed magic properties (Duncan-Kemp, 1952).

Within this stage, some interaction between protomusic and protolanguage was likely to have occurred. Consider the case of lullabies and motherese, both of which can be related to our prolonged (in comparison with other primates) altriciality period and shortening of interbirth intervals that posed the need for collective caretaking of multiple children. It is quite possible that specific musical intonations that globally characterize lullabies (e.g., the descending leaps by about 300 cents—Fernald, 1992; Reigado et al., 2011) might have been cultivated within the motherese throughout the millennia of its application. Because we still lack precise knowledge of cognitive and behavioral features of earlier hominins (including their social life), we cannot rule out the possibility that Neanderthals (and, perhaps, Denisovans) also exhibited some sort of protomusic since they have been hypothesized to share with humans the basic capacity to sing (Mithen, 2005) and to have had some form of culture, particularly symbolic behavior (Mellars, 1996; D’Errico et al., 2003).

Around 200 kya, the long Riss Glaciation began, and climatic conditions became harsher. Frequent alternations of extreme cooling and warming caused significant fluctuations in sizes of social groups. Depopulation periods increased the value of cooperation in harsh environments, strengthening bonds and stimulating interpersonal communication. During subsequent periods of demographic growth, newly established patterns of communication were cultivated over larger territories and involved a larger number of people. The seesaw demographic alternations favored selection for increased prosociality and promoted personal and interpersonal uses of protomusical behaviors.

Two formats—solitary musicking to entertain oneself during prolonged solitary activities (the babbling model) and duetting of closely related persons (the motherese model) during the the times of depopulation—provided a fertile ground for the invention of “musical mode.” Bonded couples intuitively matched the sonic characteristics of their vocalizations, as observed in modern-day motherese, and solitary musicking gave an opportunity to explore the combinatorial capacities of the matched common patterns of expression. The resulting set of sounds that pleased the sensibilities of music-makers was thereafter conserved for future musicking, available for those who overheard such musicking. Tone-matching probably originated from mother-infant interaction, characterized by instinctive mutual imitation of the expressive vocal attributes (Malloch & Trevarthen, 2009) and fueled by oxytocin (Harvey, 2020). Much of this mimicking is confined to the domain of timbre, which makes it the most likely substrate for the earliest musical modes. A set of timbre-classes, selected and repeatedly used to express specific semantic contents, constitutes what can be called a “musical timbral mode” (Nikolsky et al., 2020), which is the type of music we hypothesize for Stage 2 in our model.

The “natural” (anatomy-driven) rules of binding acoustic properties of an auditory signal with emotional semantic content, typical for animal vocal communication, were ultimately replaced by “cultural” conventions that often violated the “natural” order of things (as is characteristic of present-day human music). Here, the peculiar institution of *personal song* must have been particularly instrumental (Nikolsky et al., 2020). In numerous music cultures of Indigenous hunter-gatherers of the extreme North, whose lifestyle comes the closest to that of early humans during the Quaternary glaciation, each person is assigned a song that indicates one’s place of origin, ethnicity, kin, age, occupation, and personality type (Nikolsky et al., 2020; Sheikin, 2002). The information conveyed in a personal song is crucial for avoidance of incest in marriages in lightly populated areas. Its honest use is protected by a widespread ancestor cult and by social conventions imperative for one’s survival in harsh environments.

All in all, personal song presents a likely model for a transitory stage between the animal-like protomusic and the full-fledged human music. Personal song resembles animal songs in marking territoriality and ancestrality while assisting mating (see Bradbury and Vehrencamp, 2011). But in sharp contrast to instinct-driven animal songs, parents in Indigenous societies actually “compose” personal songs for their newborns—they deliberately use tone-classes (entailing timbre, rhythmo-meter, and pitch contours) to represent the child’s temper that they observe during the first days of parenting. Ultimately, the coexistence of personal songs and timbre-oriented music traditions among numerous ethnicities of Siberia and the Russian Far East, as well as the inherent spatial limitation of timbral music (timbral modulations are practically inaudible beyond the distance of a few meters), make a timbre-oriented personal song a very likely candidate for the forms of music characterizing our Stage 2. The development of personal song is directly related to the ongoing reduction in reactive aggression since the circulation domain of one’s personal song is limited to one’s extended family and characterized by greater tolerance in comparison to relations with outsiders. Also, the ongoing everyday musicking by individual owners of a personal song was likely to promote greater emotional control, thereby contributing to the general reduction of interpersonal conflicts within a community. The evidence of such a mediative and regulatory role of music has been provided by numerous recent studies of the enhancing influence of music on the inhibitory control in children (Bolduc et al., 2021; Bugos et al., 2022; Hennessy et al., 2019; Joret et al., 2017; Moreno & Farzan, 2015).

Around 110 kya, the Riss-Würm Interglacial ended, and the climate deteriorated again, leading to the Last Glaciation, which lasted until 10 kya. This period, when HSD reached its peak (Cieri et al., 2014), and behavioral modernity spread over most parts of the world, we see as Stage 3 in our model. For music, the primary achievement toward the end of this stage was the emergence of cross-cultural pitch orientation, evident in the uncovering of more than a hundred “bone flutes” in caves, often in bundles, over a wide region from Germany to Spain, dated to 36–30 kya (Morley, 2013). Similarities in their construction (D’Errico et al., 2003) suggest the ongoing cultural interaction throughout 45–30 kya along the Danube corridor (Higham et al., 2012).

The rise of pitch orientation can be attributed to several factors. Between around 10 and 110 kya, caves with fire became common places for human occupation (Kempe, 1988). Cave reverberation is distortive for timbre-classes but resonant for pitch-classes (e.g., it makes familiar voices unrecognizable but amplifies a pitch value). Reflections from the walls make pitch changes more salient due to the prolonged decay of each sustained pitch level. Inhabited Paleolithic caves usually resonate at a specific frequency, about 110 Hz (Devereux, 2006), and contain stalactites usable as lithophones—in some caves they produce sophisticated scales (Dams, 1985). Both resonance and lithophones might have provided the reference pitch for singing. The most resonant locations in such caves often contain paintings, dated from 35 kya onward (Díaz-Andreu & García, 2012). The same affiliation characterizes “sounding rocks,” some of which contain marks of hitting, indicative of their ritual musical usage (Morley, 2013). Most Paleolithic “bone flutes” were uncovered in caves (Morley, 2013), testifying to the pitch orientation of European Paleolithic cave-dwellers.

Another pitch-inducing factor is the intuitive tuning-in that occurs, when numerous singers try to sing the same melody: they tend to resolve sustained inharmonious combinations of tones (Zarate et al., 2010) into harmonically “perfect” intervals of unison, octave, fifth, and fourth (Tallmadge, 1984). All things considered, cave singing had the power to direct singers’ attention to the fundamental frequency and harmonicity, while promoting timbral uniformity in pitch changes. Together with the above-mentioned tendency of chain transmission to discretize pitch, these conditions were likely to convert earlier timbral modes into pitch-sets, thereby widening the collective use of music and promoting the reduction in reactive aggression. In turn, the self-domestication features promoted extensive prosociality, favorable for communal cave living and collective use of music.

The advent of the Holocene marks the final stage in our model, roughly 10 kya, when population growth resulted in prolonged intergroup contacts, extensive social networks for trade and intermarriage, and, in many cases, escalated conflicts between larger human groups. A new type of aggression—proactive—became widespread. All of these promoted a new type of music that entailed standardized intervallic typologies and tuning, as well as prescriptive rules for combining pitch-classes. Standardization of pitch- and interval-classes and pitch- and interval-sets inevitably reduces the diversity of musical modes, necessitating the institution of formal music training and introducing the notion of musical error (Nikolsky, 2016a). Music becomes professionalized and regulated by political or religious authorities. Rather free and loose usage of a multitude of musical modes that characterizes all-inclusive musicking in folk family and village traditions gives way to restrictive (“correct”) implementation of just a handful of musical keys, often supported by some sort of musical notation. Such transformation is documented in the history of ancient Babylonian (Dumbrill, 2005) and Greek (West, 1992) music systems.

Standardization of keys boosted the development of orchestral and choral music, invention of instrumental families, and the genesis of cyclic music forms that contained contrasting movements—complexities that were inaccessible before the standardization (Nikolsky, 2016b). Music, performed and auditioned en masse in service of the state or/and religion, became a political weapon in hostilities between countries, consolidating citizens across kins, clans, and castes against the supposed negative influence of neighboring cultures. Political use of music and language, where language unites communities by conveying ideas and reasons for their support while music backs the language by instilling the appropriate emotional states, has culminated in the twentieth century, comprising official propaganda in the majority of the world’s nation-states. For this reason, we periodize this fourth stage as continuing until the present.

## Conclusion

In this paper, we have outlined our model of the coevolution of music and language under the influence of aggression management throughout human evolution. Enabled by the reduction in reactive aggression—due to a number of paleo-environmental factors—music and language started as undifferentiated forms of emotional and referential signaling within musilanguage. Initially, they abided by the principles of animal communication, relying on the single-signal “monologic” display of the signaler’s affective state and the deictic reference to something observable to the signaler. Growing control of aggression within the basic family units promoted development and intergenerational transmission of patterns of communication, eventually forming two autonomous systems.

- Protomusic specialized in regulating the emotional states of individuals in their solitary activities and everyday interactions.
- Protolanguage specialized in timely delivery of referential information (including live streaming) and directed and coordinated important collective activities.

Capacity of music to promote empathy and bonding favored the formation and transmission of lexical and grammatical conventions instrumental for the complexification of language. Crystallization of musical timbral modes marked the bifurcation of music and speech.

- Music focused on the aesthetic appreciation of sonic attributes, evolving toward the selection of holistic idiomatic patterns whose acoustic properties were suitable for evoking specific emotional states common to a given lifestyle and provided easy integration of these patterns into a continuous stream.
- Language focused on effective encoding of important referential information, evolving toward the selection of contrasting, easy-to-process phonemes, the combination of which could supply enough words to refer to the surrounding objects and frequently occurring events.

Hence, language headed toward *symbolic* semiosis, driven by the need to quickly update information, in contrast to music heading to *iconic* semiosis, to satisfy the need to secure emotional contagion by means of prolonged exposure to a specific musical emotion.

Increased cooperation and social interaction favored the emergence of pitch-oriented music, which became effective at long-distance communication to a large number of people. Subsequently, pitch orientation turned into a tool of social mediation, forging formats of collective performance that distinguished music from language to an even greater extent. Speakers took turns, whereas singers sang together. At this point, music counterbalanced language along the axis of opposition of “me” versus “us.” Language supported individual awareness, bringing to light differences between individual interlocutors, whereas music carried the opposite effect of emphasizing what was in common between multiple performers.

In the long run, language promoted individualization and analysis, offset by music that promoted integration and synthesis. Music compensated for the negative social and psychological effects of language use (e.g., propensity of individualization to lead to intergroup conflicts), while language compensated for the potential negative side effects of music (e.g., suppression of individual interests in favor of the interests of an entire social group). The antithesis and mutual compensation of music and language were further intensified as both reached their exoteric stages. Music became the means of inspiring masses to feel a certain way (most commonly, patriotic, family-bound, and religious), whereas language became the instrument of reasoning, frequently counterposed to “feelings.” Music and language developed an antinomy of “heart” versus “mind.” Their dichotomy still fuels our cultural life today.

Overall, we have argued for a gradual coevolution of different types of music and of languages as the structure of human groups became more complex and diversified as a result of changing the balance between reactive and proactive forms of aggression. If early stages in the evolution of music and language were characterized by the curbing of reactive aggression, later stages became associated with the rise of and increase in proactive aggression. Our model provides a unified view of the evolution of language and music under the effects of changes in human cognition and behavior, which can and should be tested by subsequent studies.

## Electronic Supplementary Material

Below is the link to the electronic supplementary material.

Supplementary Material 1

## Data Availability

Not applicable.

## Footnotes

1 A striking example can be found in the shift in perspective of Bruno Nettl, a prominent Western ethnomusicologist. In 2000 he advocated the need for the comparative study of cross-cultural universalities in the world’s music cultures (Nettl, 2000) and then just ten years later he denied the validity and possibility of such studies (Nettl, 2010).

2 A serious miss in Western ethnomusicological publications that claim fundamental non-universality of music is that their analyses of cultural diversity zooms into the *between-culture variations* without much consideration for the *within-culture variations* that can significantly exceed the between-culture variations—quite similar to genetic diversity (Rzeszutek et al., 2012).

3 Here we briefly address the controversy on this issue between cognitive scientists and Western ethnomusicologists. Although the concepts of consonance and dissonance have been forged within the Western civilization, their dichotomy is based on the biologically rooted mechanisms of auditory perception, present among not only humans but at least some animal species (Chiandetti & Vallortigara, 2011; Hulse et al., 1995; Izumi, 2000; Koda et al., 2013; Tramo et al., 2001; Watanabe et al., 2005). Consonant and dissonant combinations of tones are distinguished by means of detection of acoustic roughness and fusion of tones (Terhardt, 1974a, b)—detectable soon after birth (Trainor et al., 2002; Zentner & Kagan, 1998), even for children of deaf parents (Masataka, 2006). See Nikolsky, 2022, for discussion.

4 By cultural invention we mean a new set of behavior adopted by a group of people and passed on to others within that group as well as outside of it.

5 This approach follows the “central neoformation” model, proposed by Vygotsky to describe and explain the general cognitive development (ontogenetic and phylogenetic) and further elaborated by his school: Alexander Luria, Aleksey Leontyev, Piotr Galperin, Daniil Elkonin, and Aleksander Zaporozhets, who headed a lab that researched music perception (Zaporozhets, 1985) (see the discussion in Nikolsky, 2022). According to this model (van der Veer & Valsiner, 1991; Vygotsky, 1987, 2013), each developmental period is forged by the necessity to master a new cognitive function that is imperative for handling critical environmental challenges. Vygotsky called it “central function” because, once established, it remains stable throughout the entire period, laying the foundation for the neoformation of the next stage. This way, the entire line of development becomes cumulative and modular. Each progressive period is characterized by the increase of cultural diversity in implementation of each central neoformation.

6 Thus, the “physical aid” function relies on the presence of rhythmic idioms embedded in a metric grid and sustained in a tempo range suitable for accompanying specific physical activities (e.g., walking for marches or lulling for lullabies). Once forged, such idioms start conserving the compositional arrangement of music that supports the same character of movement—e.g., all march-like music within the same musical culture shares the same metro-rhythmic design within the same tempo range, whether this music belongs to a genre of march or is a part of some other genre, such as symphony, opera, ballet, suite, song, sonata, or concerto.

7 For example, “emotional regulation” requires the functions of “emotional communication,” “interpersonal bonding,” and “physical aid” to be already established. To successfully choose music to change or “lock in” a specific emotional state, one must know that music can communicate musical emotions, that these emotions can bring participants to share the same emotional state and thereby bond together, and that the presence of specific structural patterns in music can match or mismatch certain activities. In turn, the “emotional regulation” function must be available in order for “compliance to norms” to emerge.

8 Many more functions have been identified in the literature on music. However, we were interested only in those functions whose existence was experimentally confirmed, reported in numerous music cultures, secured by a well-established tradition of production and/or consumption, and characterized by objectively recognizable structural features. We did our best to get rid of redundancies and minor variations in functionality by grouping similar functions into a single type according to the common mental operation(s) they require. So, our list presents 14 basic operational types to illustrate this novel method of inquiry into the evolution of music. Other researchers are welcome to improve it.

9 It is difficult to define the operational dependencies for each of the musical functions established in the literature. Arguments for their tentative order in Fig. 1 are presented in Nikolsky & Benítez-Burraco 2022. The ultimate ordering requires thorough experimental research. The connection of operational functions with specific musical genres, outlined in the above-mentioned paper, and with those musical structures that characterize such genres will enable researchers to test exactly how various types of listeners perceive music that specializes in each of the musical functions.

10 Attaining expertise in Western music cultures is usually confined to performance (e.g., composers, improvisers, and interpreters of preexisting music) but sometimes occurs in applications related to listening and appreciation of music (music critics, reviewers, competition jurors, programmers, DJs, radio hosts, sound designers, etc.). In any case, connoisseurship in music enables one to monetize their music skills one way or another. However, this opportunity is available to relatively few, specially gifted individuals and demands much time, effort, and, usually, some luck.

11 The earliest evolutionary forms of musicking that occur during such activities as motherese, grunting during grooming, humming during leisure time, or personal songs should not be considered “musical genres.” Musical genre is a specific type of music distinguished by similarity of musical structures, semantic content, usage, and manner of expression within a community of music-users (see Fabbri et al., 1982; Kallberg, 1988; Korobova, 2007; Lobanova, 2013; Nazaikinsky, 2013; Samson, 2001; Samuels, 2004; Sokhor, 1968, 1971; Tsaryova, 1976; Tsukkerman, 1964). Thus, lullabies can be identified by their ongoing repetitions of brief motivic formulas that contain soft, descending seconds and minor thirds in slow tempo, applicable to lulling. Most lullabies share such features and therefore can be recognized even when they are arranged for different performance settings, such as a symphonic orchestra. Equally recognizable are the semantic characteristics of lullabies—they are distinguished by general relaxation of the listeners, probably due to the influence of the music structures associated with gentle rocking at a regular sustained pace. This relaxing effect was found even in infants who responded to unfamiliar foreign lullabies (Bainbridge et al., 2021). Musical genres are extremely important for the generation of musical idioms. However, motherese or personal songs fall short of constituting a genre—their typology is too diffused and individualized to canonize a specific set of expressive means capable of characterizing a particular topic. For this reason, we prefer to qualify the structural and semantic uniformity of specific applications of protomusic as protogenres.

12 A musical genre can be considered “basic” if it cannot encompass another genre and is directly used by the majority of population within a music culture in folk transmission (Korobova, 2007; Lobanova, 2013; Nazaikinsky, 2013; Tsukkerman, 1964). Genres that “house” smaller genres (e.g., opera incorporates such genres as overture, entr’acte, recitative, aria, duet, trio, song, ballet, etc.) can be considered “complex.” Usually, they emerge in societies that use music notation, formal music theory, and professionalized forms of music.

13 Objectivization of music and the resulting qualitative change in discrimination of pitch from timbre-based to frequency-based musicking was demonstrated in series of experiments conducted by Zaporozhets and his research group (Endovitskaya, 1964; Lisina, 1966; Mukhina & Lisina, 1966; Repina, 1966; Zaporozhets, 1985) and by Leontyev (2009), as well as in the earlier research by Teplov (1947). Their model of gradual transformation of the timbre-oriented “verbal hearing” into the pitch-oriented “musical hearing” was confirmed in numerous methodological studies of early music education (which was obligatory in the USSR, and therefore studied on a sample pool of millions of people). Such studies have been systematically conducted and reviewed from the late 1920s onward (see Nikolsky, 2022).

14 The ontogenetic model for this is known as “attrition of music skills” (see Nikolsky, 2020b). An equivalent phylogenetic model can be conceived along the same lines.

15 It is worth noting that music allows an individual to experience a specific emotional state and share it with others in a collective performance, thereby supporting emotional interaction even though each participant may have very different outlooks and goals—what Ian Cross (e.g., 2009) regarded as provision of “safe space” expression for each individual.

16 The modernistic and post-modernistic Western anti-emotional stance should be regarded as a peculiar manneristic episode in the millennia-long history of the world’s music (spanning from at least ca. 40 kya). And even within this chronologically and geographically narrow, uniquely Western historical period, anti-emotionalism is fractional: all commercially viable forms of music (that is, satisfying a public demand) still keep engaging musical emotions. Even Western avant-garde music included styles clearly affiliated with deliberate emotional expression and traditional genres (e.g., pre-WWII, Bartok and Berg as opposed to Stravinsky, and post-WWII, Nono, Lutoslawski, and Schnittke as opposed to Boulez, Xenaxis, and Stockhausen).

17 On the other hand, attempts to omit the issue of musical emotions and define music in a most general way as “a creative play with sound” (e.g., Brandt et al., 2022) seem insufficient—not testable by falsification. Many forms of poetry, including traditional folk epic and lyric genres, also feature what can be called “creative play” with phonetic (i.e., timbral), rhythmo-metric, dynamic, and prosodic aspects of linguistic expression, all of which are shared with music. Furthermore, if poetry can be argued to constitute an intentionally “musicalized” use of language, some sounds that by design clearly have nothing to do with music satisfy its definition as creative play. At the extreme, car manufacturers design engine noises to excite or please the driver while distinguishing between different operational modes (starting the engine, speeding up, changing gears, etc.). For example, see: https://www.volkswagenag.com/en/news/stories/2019/03/volkswagen-sound-design.html. Despite clearly falling off the scope of music (according to the public convention), such sound engineering also satisfies the “creative play” definition of music.

18 The compositionality of music becomes evident whenever the same tune is used in different musical traditions—the very same motifs and rhythms receive different arrangements according to different compositional principles. Such are the compositions by a Polish prisoner of war, Wojciech Bobowski (1610–1675), also known as Ali Ufki, from his treatise *Mecmua-yi saz ü söz* [Collection of Instrumental and Vocal Pieces], rearranged according to the homophonic principles by Dimitrius Cantemir (1683–1723), a Moldavian prince, in his treatise *Kitāb-i ‘ilmu ‘l mūzīkī ‘ala Vechi’l-Hurufat* [Book on the Science of Music].

19 All standard music forms commonly used today in one way or another descend from the theory of music rhetoric as it has been elaborated in the sixteenth to eighteenth centuries, before Adolf Marx standardized the most common schemes of arranging themes and their development in the beginning of the nineteenth century. Thus, the treatment of rhetorical canons—*prooemium*, *divisio*, *confirmatio*, *confutatio*, *peroratio*—in the domain of music became embedded in the so-called sonata form (Bonds, 1991; Marx & Burnham, 1997; Moyer, 1969).

20 Here we note the similarity between the transmission of specific music structures and genetic transmission in light of the general similarity between variation in genetics and variations in music styles (Brown et al., 2013; Le Bomin et al., 2016; Pamjav et al., 2012).

21 For this reason, the distinction between animal calls and songs has nothing to do with the distinction between human calls (e.g., the genre of forest-calls) and songs. Both distinctions emerged at different times, determined by different factors, and followed different evolutionary trajectories. A fundamental reorganization of expressive means in human musical communication in comparison with animal communication probably took place during the Paleolithic, resulting in the complete reversal of semantic values for a number of acoustic attributes of typical animal vocalizations (see Nikolsky, 2020b).

22 Although some researchers equate musical and linguistic syntax, the parallels between pitch and interval classes and sets, chordal typology, and key structure of music on the one hand and, correspondingly, phonological, morphological, and morphonological organizations of language are limited to only the latest stage of evolution of music, specifically as it is implemented in the Western classical music tradition (see Nikolsky & Benitez-Burraco, 2022). The majority of known non-Western traditional musical systems do not subscribe to the principles of Western tonality. For such musical systems, similarities between musical and linguistic syntax are confined to the elementary, “surface” level of tonal organization—what music theorists qualify as “motivic” rather than “phrasal” level of compositionality.

23 It is not a trivial task to explain the brain divergence of processing architectures for music and language. Recently, Dehaene and colleagues (2022) have argued that music and language, as well as several other human-distinctive abilities, such as mathematics, might depend on parallel circuits endowed with similar computational principles, which the authors construed as internal languages of thought, akin to computer languages. These languages are claimed to recursively compute small sets of symbols—the authors call them “primitives”—whose nature varies from one domain to another (merge and antisymmetry for language, but pitch and rhythm for music). These mental programs which encode nested repetitions with variations contrast with the simpler computational models that can capture nonhuman primate data. Interestingly for our concerns here, Dehaene and colleagues have hypothesized that some mutation(s) in the human lineage might have granted compositionality to many, possibly all, human cortical circuits. This circumstance would account for the fact that although these circuits process different types of “primitives” and differ in their styles (e.g., pitch and rhythm for music, merge and antisymmetry for language), they exhibit similar computational properties (particularly, the ability to process symbolic items recursively).

24 Here and onward, the dates we mention are approximate. There is considerable controversy around the exact timing of the emergence of the human species, as well as its place (one single place or multiregional) and its abruptness or graduality, particularly in light of the extensive admixture with other hominins (see Meneganzin & Bernardi, 2023; Neubauer et al., 2018; Scerri et al., 2019, among many others, for interesting discussions).

## Declarations

### Publication History

A previous, less-elaborate version of this manuscript has been posted as a preprint to PsyArXiv: https://psyarxiv.com/xfbqn/.

## References

[CR1] Abecasis, D., Brochard, R., Granot, R. Y., & Drake, C. (2005). Differential brain response to metrical accents in isochronous auditory sequences. *Music Perception*, *22*(3), 549–562. https://doi.org/10/b6zw26

[CR2] Alekseyev, E. Ye. (1976). *Problems in the genesis of musical mode (on the example of Yakut folksong): Analysis [Проблемы формирования лада (на материале якутской народной песни): Исследование]*. Muzyka.

[CR3] Alekseyev, E. Ye. (1986). *Musical intonation in the earliest forms of folklore. The aspect of pitch [Раннефольклорное интонирование: Звуковысотный аспект]*. Soviet Composer. http://eduard.alekseyev.org/rfi/index.html

[CR4] Alekseyev, E. Ye. (1988). *Folklore in the context of modern culture: Thoughts on the future of folk song [Фольклор в контексте современной культуры: Рассуждения о судьбах народной песни]*. Soviet Composer. http://eduard.alekseyev.org/fic/index.html

[CR5] Altenmüller E, Kopiez R, Grewe O, Bader R (2013). Strong emotions in music: Are they an evolutionary adaptation?. Sound—perception—performance.

[CR6] Alworth LC, Buerkle SC (2013). The effects of music on animal physiology, behavior and welfare. Lab Animal.

[CR7] Argstatter H (2016). Perception of basic emotions in music: Culture-specific or multicultural?. Psychology of Music.

[CR8] Arom, S. (2006). The Aksak rhythm: Structural aspects versus cultural dimensions. In M. Baroni, A. R. Addessi, R. Caterina, & M. Costa (Eds.), *Proceedings of the 9th International Conference of Music Perception and Cognition* (pp. 1882–1883). University of Bologna.

[CR9] Asano R (2022). The evolution of hierarchical structure building capacity for language and music: A bottom-up perspective. Primates.

[CR10] Ashton, N. M., & Davis, R. J. (2021). Cultural mosaics, social structure and identity: The Acheulean threshold in Europe. *Journal of Human Evolution*, 156. 10.1016/j.jhevol.2021.103011. 10.1016/j.jhevol.2021.103011 34102521

[CR11] Bainbridge CM, Bertolo M, Youngers J, Atwood S, Yurdum L, Simson J, Lopez K, Xing F, Martin A, Mehr SA (2021). Infants relax in response to unfamiliar foreign lullabies. Nature Human Behaviour.

[CR12] Balkwill, L. L., & Thompson, W. F. (1999). A cross-cultural investigation of the perception of emotion in music: Psychophysical and cultural cues. *Music Perception: An Interdisciplinary Journal*, *17*(1), 43–64. https://doi.org/10/gmnfqc

[CR13] Balzano GJ (1980). The group-theoretic description of 12-fold and microtonal pitch systems. Computer Music Journal.

[CR14] Bartel, D. (1997). *Musica poetica: Musical-rhetorical figures in german baroque music*. University of Nebraska Press.

[CR15] Baruch C, Drake C (1997). Tempo discrimination in infants. Infant Behavior and Development.

[CR16] Belfi, A. M., Karlan, B., & Tranel, D. (2015). Music evokes vivid autobiographical memories. *Memory*, *8211*(August), 1–11. https://doi.org/10/ghf3x2 10.1080/09658211.2015.1061012 26259098

[CR17] Beliayev, V. M. (1990). *Viktor Mikhailovich Beliayev [Виктор Михайлович Беляев]*. Soviet Composer.

[CR18] Bendixen, A., Háden, G. P., Németh, R., Farkas, D., Török, M., & Winkler, I. (2015). Newborn infants detect cues of concurrent sound segregation. *Developmental Neuroscience*, *37*(2), 172–181. https://doi.org/10/f68v3h 10.1159/000370237 25721916

[CR19] Benítez-Burraco A, Progovac L (2020). A four-stage model for language evolution under the effects of human self-domestication. Language & Communication.

[CR20] Benítez-Burraco, A., & Progovac, L. (2021). Language evolution: Examining the link between cross-modality and aggression through the lens of disorders. *Philosophical Transactions of the Royal Society B: Biological Sciences, 376*, 20200188. https://doi.org/10/gns57p 10.1098/rstb.2020.0188 PMC8059641 33745319

[CR21] Benítez-Burraco A, Ferretti F, Progovac L (2021). Human self-domestication and the evolution of pragmatics. Cognitive Science.

[CR22] Benjamin, T., Horvit, M. M., & Nelson, R. (2015). *Techniques and materials of music: From the common practice period through the twentieth century*. Cengage Learning.

[CR23] Bergeson TR, Trehub SE (2006). Infants’ perception of rhythmic patterns. Music Perception.

[CR24] Berry, J. W., Segall, M. H., Dasen, P. R., & Poortinga, J. W. (2002). *Cross-cultural psychology: Research and applications*. Cambridge University Press.

[CR25] Besson M, Schön D (2001). Comparison between language and music. Annals of the New York Academy of Sciences.

[CR26] Bidelman, G. M., & Krishnan, A. (2009). Neural correlates of consonance, dissonance, and the hierarchy of musical pitch in the human brainstem. *The Journal of Neuroscience*, *29*(42), 13165–13171. https://doi.org/10/fvn6sm 10.1523/JNEUROSCI.3900-09.2009 PMC2804402 19846704

[CR27] Bispham, J. (2018). The human faculty for music: What’s special about it? PhD thesis, University of Cambridge. 10.17863/CAM.31835

[CR28] Blacking J (1977). Can musical universals be heard?. The World of Music.

[CR29] Boaz, N. T., & Ciochon, R. L. (2004). *Dragon Bone Hill: An ice-age saga of Homo erectus*. Oxford University Press.

[CR30] Boeckx C, Benítez-Burraco A (2014). The shape of the human language-ready brain. Frontiers in Psychology.

[CR31] Boer, D., & Fischer, R. (2012). Towards a holistic model of functions of music listening across cultures: A culturally decentred qualitative approach. *Psychology of Music*, *40*(2), 179–200. https://doi.org/10/dxwn8v

[CR32] Boivin, N., Brumm, A., Lewis, H., Robinson, D., & Korisettar, R. (2007). Sensual, material, and technological understanding: exploring prehistoric soundscapes in South India. *Journal of the Royal Anthropological Institute 13*(2), 267–94. https://doi.org/10/dpghp2

[CR33] Bolduc J, Gosselin N, Chevrette T, Peretz I (2021). The impact of music training on inhibition control, phonological processing, and motor skills in kindergarteners: A randomized control trial. Early Child Development and Care.

[CR34] Bonds, M. E. (1991). *Wordless rhetoric: Musical form and the metaphor of the oration*. Harvard University Press.

[CR35] Bradbury JW, Lee Vehrencamp S (2011). Principles of animal communication.

[CR36] Brandl, R. (2008). New considerations of diaphony in Southeast Europe. In A. Ahmedaja, & G. Haid (Eds.), *European voices, vol. 1: Multipart singing in the Balkans and the Mediterranean* (pp. 281–297). Böhlau Verlag.

[CR37] Brandt, A., Gebrian, M., & Slevc, L. R. (2022). *Music and language: Milestones of development*. PsyArXiv. 10.31234/osf.io/57a9w

[CR38] Bregman, A. S. (1994). *Auditory scene analysis: The perceptual organization of sound*. MIT Press.

[CR39] Brooks J, Yamamoto S (2021). The founder sociality hypothesis. Ecology and Evolution.

[CR40] Brown S, Brown S, Merker B, Wallin NL (2000). The “musilanguage” model of language evolution. The origins of music.

[CR41] Brown, S. (2005). How does music work?” Toward a pragmatics of musical communication. In U. Volgsten, & S. Brown (Eds.), *Music and manipulation: On the social uses and social control of music* (pp. 1–27). Berghahn Books.

[CR42] Brown, S. (2017). A joint prosodic origin of language and music. *Frontiers in Psychology*, *8*, 1894. https://doi.org/10/gchkg7 10.3389/fpsyg.2017.01894 PMC5666296 29163276

[CR43] Brown, S., & Jordania, J. (2013). Universals in the world’s musics. *Psychology of Music*, *41*(2), 229–248. https://doi.org/10/bhnxdh

[CR44] Brown S, Martinez MJ, Parsons LM (2006). Music and language side by side in the brain: A PET study of the generation of melodies and sentences. The European Journal of Neuroscience.

[CR45] Brown, S., Savage, P. E., Ko, A. M., Stoneking, M., Ko, Y. C., Loo, J. H., & Trejaut, J. A. (2013). Correlations in the population structure of music, genes and language. *Proceedings of the Royal Society B: Biological Sciences*, *281*(1774), 20132072. 10.1098/rspb.2013.2072 10.1098/rspb.2013.2072 PMC3843827 24225453

[CR46] Bugos JA, DeMarie D, Stokes C, Power P (2022). Multimodal music training enhances executive functions in children: Results of a randomized controlled trial. Annals of the New York Academy of Sciences.

[CR47] Cazden, N. (1959). Musical intervals and simple number ratios. *Journal of Research in Music Education*, *7*(2), 197–220. https://doi.org/10/cttptc

[CR48] Cazden N (1972). The systemic reference of musical consonance response. International Review of the Aesthetics and Sociology of Music.

[CR49] Cazden, N. (1980). The definition of consonance and dissonance. *International Review of the Aesthetics and Sociology of Music*, *11*(2), 123–168. https://doi.org/10/ccqc4r

[CR50] Chen, X., Affourtit, J., Ryskin, R., Regev, T. I., Norman-Haignere, S., Jouravlev, O., Malik-Moraleda, S., Kean, H., Varley, R., & Fedorenko, E. (2021). The human language system does not support music processing. bioRxiv. 10.1101/2021.06.01.446439 10.1093/cercor/bhad087 PMC10505454 37005063

[CR51] Chiandetti, C., & Vallortigara, G. (2011). Chicks like consonant music. *Psychological Science*, *22*(10), 1270–1273. https://doi.org/10/fv76f8 10.1177/0956797611418244 21934134

[CR52] Cieri, R. L., Churchill, S. E., Franciscus, R. G., Tan, J., & Hare, B. (2014). Craniofacial feminization, social tolerance, and the origins of behavioral modernity. *Current Anthropology*, *55*(4), 419–443. https://doi.org/10/gcx737

[CR53] Clarke E, DeNora T, Vuoskoski J (2015). Music, empathy and cultural understanding. Physics of Life Reviews.

[CR55] Clayton, M. R. L. (2000). *Time in indian music: Rhythm, Metre, and form in north indian rag performance*. Oxford University Press.

[CR54] Clayton, M. (2016). The social and personal functions of music in cross-cultural perspective. In S. Hallam, I. Cross, & M. Thaut (Eds.), *The Oxford handbook of music psychology* (second ed., pp. 47–59). Oxford University Press.

[CR56] Collier GL, Collier JL (2007). Studies of tempo using a double timing paradigm. Music Perception: An Interdisciplinary Journal.

[CR57] Cook, N. D. (2002). *Tone of voice and mind: The connections between intonation, emotion, cognition and consciousness*. John Benjamins.

[CR58] Corballis, M. C. (2020). Crossing the Rubicon: behaviorism, language, and evolutionary continuity. *Frontiers in Psychology, 11*, 653. https://doi.org/10/gmnfzr 10.3389/fpsyg.2020.00653 PMC7186390 32373020

[CR59] Cowley, S. J., & Kuhle, A. (2020). The rise of languaging. *Biosystems, 198*, 104264. https://doi.org/10/gmnfzs 10.1016/j.biosystems.2020.104264 33068672

[CR60] Cross I (2009). The evolutionary nature of musical meaning. Musicae Scientiae.

[CR61] Cross I, Fitch WT, Aboitiz F, Iriki A, Jarvis ED, Lewis J, Arbib MA (2013). Culture and evolution. Language, music, and the brain: A mysterious relationship.

[CR62] D’Errico, F., Henshilwood, C., Lawson, G., Vanhaeren, M., Tillier, A. M., Soressi, M. (2003). Archaeological evidence for the emergence of language, symbolism, and music — An alternative multidisciplinary perspective. *Journal of World Prehistory 17*, 1–70. https://doi.org/10/b7tq3n

[CR63] Dachkovsky, S., Stamp, R., & Sandler, W. (2018). Constructing complexity in a young sign language. *Frontiers in Psychology, 9*, 2202. https://doi.org/10/gnt2xz 10.3389/fpsyg.2018.02202 PMC6306080 30618892

[CR64] Dalla Bella S, Peretz I, Rousseau L, Gosselin N (2001). A developmental study of the affective value of tempo and mode in music. Cognition.

[CR65] Dams, L. (1985). Palaeolithic lithophones: descriptions and comparisons. *Oxford Journal of Archaeology, 4*(1), 31–46. https://doi.org/10/d8xr5s

[CR66] Dasen, P. R. (2012). Emics and etic in cross-cultural psychology: Towards a convergence in the study of cognitive styles. In T. M. S. Tchombe, A. B. Nsamenang, & H. K., & M. Fülöp (Eds.), *Proceedings of the 4th Africa Region Conference of the IACCP, University of Buea, Cameroun, Aug. 1–8, 2009* (pp. 55–73). University of Buea.

[CR67] de Boer B, Ravignani A (2021). Joint origins of speech and music: Testing evolutionary hypotheses on modern humans. Semiotica.

[CR68] de Boer B, Zuidema W (2010). Multi-agent simulations of the evolution of combinatorial phonology. Adaptive Behavior.

[CR69] Dehaene S, Al Roumi F, Lakretz Y, Planton S, Sablé-Meyer M (2022). Symbols and mental programs: A hypothesis about human singularity. Trends in Cognitive Sciences.

[CR70] Devereux P, Scarre C, Lawson G (2006). Ears and years: Aspects of acoustics and intentionality in antiquity. Archaeoacoustics.

[CR71] Díaz-Andreu, M., & García, B. (2012). Acoustics and Levantine rock art: auditory perceptions in La Valltorta gorge (Spain). *Journal of Archaeological Science 39*(12), 3591–99. https://doi.org/10/gmnfhn

[CR72] Dingemanse, M., Blasi, D. E., Lupyan, G., Christiansen, M. H., & Monaghan, P. (2015). Arbitrariness, iconicity, and systematicity in language. *Trends in Cognitive Science, 19*(10):603–615. doi: 10.1016/j.tics.2015.07.013. PMID: 26412098. 10.1016/j.tics.2015.07.013 26412098

[CR73] Dissanayake, E. (2005). Ritual and ritualization: Musical means of conveying and shaping emotion in humans and other animals. In S. Brown, & U. Volgsten (Eds.), *Music and manipulation: On the social uses and social control of music* (pp. 31–56). Berghahn Books.

[CR74] Dorchak, G. (2016). *The aurality of rhetoric: A critical hermeneutic of Cape Breton’s rhetorical music community* PhD dissertation, University of Massachusetts, Amherst. 10.7275/7946678.0

[CR75] Drake C (1998). Psychological processes involved in the temporal organization of complex auditory sequences: Universal and acquired processes. Music Perception.

[CR76] Drake C, Bertrand D (2001). The quest for universals in temporal processing in music. Annals of the New York Academy of Sciences.

[CR77] Dumbrill R (2005). The archaeomusicology of the ancient Near East.

[CR78] Dunbar, R. I. M. (2012a). Bridging the bonding gap: the transition from primates to humans. *Philosophical Transactions of the Royal Society B: Biological Sciences, 367*, 1837–46. https://doi.org/10/f34tg7 10.1098/rstb.2011.0217 PMC3367699 22641822

[CR79] Dunbar, R. I. M. (2012b). On the evolutionary function of song and dance. In Bannan N, ed. *Music, language, and human evolution* (pp. 201–14). Oxford University Press. 10.1093/acprof:osobl/9780199227341.003.0008.

[CR80] Duncan-Kemp AM (1952). Where strange paths go down.

[CR81] Eerola, T., & Vuoskoski, J. K. (2013). A review of music and emotion studies: Approaches, emotion models, and stimuli. *Music Perception*, *30*(3), 307–340. https://doi.org/10/gmnfn9

[CR82] Egermann, H., Fernando, N., Chuen, L., & McAdams, S. (2015). Music induces universal emotion-related psychophysiological responses: Comparing Canadian listeners to Congolese Pygmies. *Frontiers in Psychology*, *5*. 10.3389/fpsyg.2014.01341 10.3389/fpsyg.2014.01341 PMC4286616 25620935

[CR83] Ellis MC (1992). Tempo perception and performance of elementary students, grades 3–6. Journal of Research in Music Education.

[CR84] Endovitskaya, T. V. (1964). Development of sensation and perception in the preschool age children [Развитие ощущения и восприятия у детей дошкольного возраста]. In A. V. Zaporozhets, & D. B. Elkonin (Eds.), *Psychology of preschool age children [Психология детей дошкольного возраста]* (pp. 13–71). Prosvesheniye.

[CR85] Fabbri, F., Tagg, P., & Horn, D. (1982). A theory of musical genres: Two applications. In D. Horn, & P. Tagg (Eds.), *Popular music perspectives* (pp. 52–81). IASPM, Göteborg and Exeter.

[CR86] Fenk-Oczlon, G. (2017). What vowels can tell us about the evolution of music. *Frontiers in Psychology*, *8*, 1581. https://doi.org/10/gbxtvj 10.3389/fpsyg.2017.01581 PMC5614962 29018371

[CR87] Fernald, A. (1992). Meaningful melodies in mothers’ speech to infants. In H. Papousek, U. Jurgens, & M. Papousek (Eds.), *Nonverbal vocal communication comparative and developmental approaches* (pp. 262–282). Cambridge University Press.

[CR88] Ferreira, M. P. R. (1997). *Music at Cluny: The tradition of Gregorian chant for the Proper of the Mass—Melodic variants and microtonal nuances*. PhD dissertation, Princeton University.

[CR89] Filippi P, Hoeschele M, Spierings M, Bowling DL (2019). Temporal modulation in speech, music, and animal vocal communication: Evidence of conserved function. Annals of the New York Academy of Sciences.

[CR90] Fitch, W. T. (2006). The biology and evolution of music: A comparative perspective. *Cognition*, *100*(1), 173–215. https://doi.org/10/d84vv8 10.1016/j.cognition.2005.11.009 16412411

[CR91] Fitch, W. T. (2010). *The evolution of language*. Cambridge University Press.

[CR92] Fitch, W. T. (2012). The biology and evolution of rhythm: Unravelling a paradox. *Language and music as cognitive systems* (pp. 73–95). Oxford University Press. 10.1093/acprof:oso/9780199553426.003.0009.

[CR93] Fitch, W. T. (2017). Cultural evolution: Lab-cultured musical universals. *Nature Human Behaviour*, *1*(1), 1–2. https://doi.org/10/gmxp2t

[CR94] Fraisse, P. (1982). Rhythm and tempo. In D. Deutsch (Ed.), *Psychology of music* (pp. 149–180). Academic Press.

[CR95] Friberg, A., & Sundberg, J. (1999). Does music performance allude to locomotion? A model of final ritardandi derived from measurements of stopping runners. *The Journal of the Acoustical Society of America*, *105*(3), 1469–1484. https://doi.org/10/b4bqxz

[CR97] Fritz, T. H., Sammler, D., & Koelsch, S. (2006). How far is music universal? An intercultural comparison. In M. Baroni, A. R. Addessi, R. Caterina, & M. Costa (Eds.), *9th International Conference on Music Perception & Cognition, Bologna, Italy* (p. 88). Bononia University Press.

[CR96] Fritz, T. H., Jentschke, S., Gosselin, N., Sammler, D., Peretz, I., Turner, R., Friederici, A. D., & Koelsch, S. (2009). Universal recognition of three basic emotions in music. *Current Biology*, *19*(7), 573–576. https://doi.org/10/dfmhjh 10.1016/j.cub.2009.02.058 19303300

[CR98] Fukase, H., Kondo, O., & Ishida, H. (2015). Size and placement of developing anterior teeth in immature Neanderthal mandibles from Dederiyeh Cave, Syria: Implications for emergence of the modern human chin. *American Journal of Physical Anthropology, 156*, 482–8. https://doi.org/10/f67qhh 10.1002/ajpa.22665 25388672

[CR99] Gabrielsson, A., & Juslin, P. N. (2003). Emotional expression in music. In R. J. Davidson, K. R. Scherer, & H. H. Goldsmith (Eds.), *Handbook of affective sciences* (pp. 503–534). Oxford University Press.

[CR100] Gill, K. Z., & Purves, D. (2009). A biological rationale for musical scales. *PLoS ONE*, *4*(12). https://doi.org/10/c8snbg 10.1371/journal.pone.0008144 PMC2779864 19997506

[CR101] Gleeson BT, Kushnick G (2018). Female status, food security, and stature sexual dimorphism: Testing mate choice as a mechanism in human self-domestication. American Journal of Physical Anthropology.

[CR102] Gourlay KA (1984). The non-universality of music and the universality of non-music. The World of Music.

[CR103] Granot, R. (2017). Music, pleasure, and social affiliation: Hormones and neurotransmitters. In R. Ashley, & R. Timmers (Eds.), *The Routledge companion to music cognition* (pp. 101–112). Routledge.

[CR104] Grauer, V. A. (1996). Toward a unified theory of the arts.Music Theory Online, *2*(6). https://mtosmt.org/issues/mto.96.2.6/mto.96.2.6.grauer.html

[CR105] Greenfield PM, Keller H, Fuligni A, Maynard A (2003). Cultural pathways through universal development. Annual Review of Psychology.

[CR106] Hare, B. (2017). Survival of the friendliest: Homo sapiens evolved via selection for prosociality. *Annual Review of Psychology, 68*, 155–86. https://doi.org/10/gdngfz 10.1146/annurev-psych-010416-044201 27732802

[CR107] Harrison, D. (1990). Rhetoric and fugue: An analytical application. *Music Theory Spectrum*, *12*(1), 1–42. https://doi.org/10/gm22pc

[CR108] Harvey, A. R. (2017). *Music, evolution, and the harmony of souls*. Oxford University Press. 10.1093/acprof:oso/9780198786856.001.0001.

[CR109] Harvey, A. R. (2018). Music and the meeting of human minds. *Frontiers in Psychology, 9*. https://doi.org/10/gdkwx6 10.3389/fpsyg.2018.00762 PMC5964593 29867703

[CR110] Harvey AR (2020). Links between the neurobiology of oxytocin and human musicality. Frontiers in Human Neuroscience.

[CR111] Haspelmath, M. (2020). Human linguisticality and the building blocks of languages. *Frontiers in Psychology, 10*, 3056. https://doi.org/10/ggjtqd 10.3389/fpsyg.2019.03056 PMC7006236 32082208

[CR112] Hefer, M., Weintraub, Z., & Cohen, V. (2009). Musical cognition at birth: A qualitative study. *Early Child Development and Care*, *179*(6), 769–783. https://doi.org/10/fdknf7

[CR113] Heffner, C. C., & Slevc, L. R. (2015). Prosodic structure as a parallel to musical structure. *Frontiers in Psychology*, *6*, 1962. 10.3389/fpsyg.2015.01962 10.3389/fpsyg.2015.01962 PMC4687474 26733930

[CR114] Hennessy SL, Sachs ME, Ilari B, Habibi A (2019). Effects of music training on inhibitory control and associated neural networks in school-aged children: A longitudinal study. Frontiers in Neuroscience.

[CR115] Higham, T., Basell, L., Jacobi, R., Wood, R., Bronk Ramsey, C., & Conard, N. J. (2012). Testing models for the beginnings of the Aurignacian and the advent of figurative art and music: the radiocarbon chronology of Geißenklösterle. *Journal of Human Evolution, 62*(6), 664–76. https://doi.org/10/f32kgd 10.1016/j.jhevol.2012.03.003 22575323

[CR116] Hockett CF (1960). The origin of speech. Scientific American.

[CR117] Honing H (2019). The origins of musicality.

[CR118] Honingh A, Bod R (2011). In search of universal properties of musical scales. Journal of New Music Research.

[CR119] Hood M (1977). Universal attributes in music. The World of Music.

[CR120] Hulse, S. H., Bernard, D. J., & Braaten, R. F. (1995). Auditory discrimination of chord-based spectral structures by European starlings (*Sturnus vulgaris*). *Journal of Experimental Psychology: General*, *124*(4), 409–423. https://doi.org/10/bwgs5j

[CR121] Hurford, J. R. (2012). *Language in the light of evolution: The origins of grammar* (2 vol.). Oxford University Press.

[CR122] Iyer, V. S. (1998). *Microstructures of feel, macrostructures of sound: Embodied cognition in West African and African-American musics*. PhD dissertation, University of California, Berkeley.

[CR123] Izumi, A. (2000). Japanese monkeys perceive sensory consonance of chords. *Journal of the Acoustical Society of America*, *108*(6), 3073–3078. https://doi.org/10/ftqh4d 10.1121/1.1323461 11144600

[CR124] Jackendoff, R. (2009). Parallels and nonparallels between language and music. *Music Perception, 26*, 195–204. https://doi.org/10/fk7m2x

[CR125] Jackendoff R, Lerdahl F (2006). The capacity for music: What is it, and what’s special about it?. Cognition.

[CR127] Jacoby, N., Undurraga, E. A., McPherson, M. J., Valdés, J., Ossandón, T., & McDermott, J. H. (2019). Universal and non-universal features of musical pitch perception revealed by singing. *Current Biology*, *29*(19), 3229–3243.e12. https://doi.org/10/ggbvj3 10.1016/j.cub.2019.08.020 PMC9907018 31543451

[CR126] Jacoby, N., Polak, R., Grahn, J., Cameron, D., Lee, K. M., Godoy, R., Undurraga, E. A., Huanca, T., Thalwitzer, T., Doumbia, N., Goldberg, D., Margulis, E., Wong, P. C. M., Jure, L., Rocamora, M., Fujii, S., Savage, P. E., Ajimi, J., Konno, R., & McDermott, J. H. (2021). Universality and cross-cultural variation in mental representations of music revealed by global comparison of rhythm priors. PsyArXiv. 10.31234/osf.io/b879v

[CR128] Jan, S. (2018). “The two brothers”: reconciling perceptual-cognitive and statistical models of musical evolution. *Frontiers in Psychology, 9*, 344. https://doi.org/10/gf7sxs 10.3389/fpsyg.2018.00344 PMC5893830 29670551

[CR129] Janata, P., Tomic, S. T., & Rakowski, S. K. (2007). Characterisation of music-evoked autobiographical memories. *Memory*, *15*(8), 845–860. https://doi.org/10/cfscj3 10.1080/09658210701734593 17965981

[CR130] Johnson EK, White KS (2020). Developmental sociolinguistics: Children’s acquisition of language variation. Wiley Interdisciplinary Reviews: Cognitive Science.

[CR131] Johnson-Laird, P. N., & Oatley, K. (2010). Emotions, music, and literature. In M. Lewis, J. M. Haviland-Jones, & L. F. Barrett (Eds.), *Handbook of emotions* (pp. 102–113). The Guilford Press.

[CR132] Jordania J (2011). Why do people sing? Music in human evolution.

[CR133] Jordania J (2017). A new model of human evolution: How predators shaped human morphology and behaviour.

[CR134] Joret ME, Germeys F, Gidron Y (2017). Cognitive inhibitory control in children following early childhood music education. Musicae Scientiae.

[CR135] Juslin, P. N. (2005). From mimesis to catharsis: Expression, perception, and induction of emotion in music. In D. Miell, R. MacDonald, & D. J. Hargreaves (Eds.), *Musical communication* (pp. 85–116). Oxford University Press.

[CR136] Juslin, P. N. (2011). Music and emotion: Seven questions, seven answers. In I. Deliège, & J. Davidson (Eds.), *Music and the mind* (pp. 113–138). Oxford University Press. http://www.oxfordscholarship.com/view/10.1093/acprof:osobl/9780199581566.001.0001/acprof-9780199581566-chapter-7.

[CR137] Juslin, P. N. (2013). From everyday emotions to aesthetic emotions: Towards a unified theory of musical emotions. *Physics of Life Reviews*, *10*(3), 235–266. https://doi.org/10/f233sd 10.1016/j.plrev.2013.05.008 23769678

[CR138] Juslin, P. N., & Laukka, P. (2003). Communication of emotions in vocal expression and music performance: Different channels, same code? *Psychological Bulletin*, *129*(5), 770–814. https://doi.org/10/ff6wbc 10.1037/0033-2909.129.5.770 12956543

[CR139] Justus T, Hutsler JJ (2005). Fundamental issues in the evolutionary psychology of music: Assessing innateness and domain specificity. Music Perception.

[CR140] Kallberg J (1988). The rhetoric of genre: Chopin’s Nocturne in G Minor. 19th-Century Music.

[CR141] Karl G, Robinson J (2015). Yet again, “between absolute and programme music. The British Journal of Aesthetics.

[CR142] Keller, H. (1973). *Phrasing and articulation: A contribution to a rhetoric of music, with 152 musical examples*. W.W. Norton.

[CR143] Kempe DRC (1988). Living underground: A history of cave and cliff dwelling.

[CR144] Kidd E, Donnelly S, Christiansen MH (2018). Individual differences in language acquisition and processing. Trends in Cognitive Sciences.

[CR145] Kirby, S., Tamariz, M., Cornish, H., & Smith, K. (2015). Compression and communication in the cultural evolution of linguistic structure. *Cognition*, *141*, 87–102. https://doi.org/10/f7jcnn 10.1016/j.cognition.2015.03.016 25966840

[CR146] Koda, H., Basile, M., Olivier, M., Remeuf, K., Nagumo, S., Blois-Heulin, C., & Lemasso, A. (2013). Validation of an auditory sensory reinforcement paradigm: Campbell’s monkeys (*Cercopithecus campbelli*) do not prefer consonant over dissonant sounds. *Journal of Comparative Psychology*, *127*(3), 265–271. https://doi.org/10/f48kmd 10.1037/a0031237 23566027

[CR147] Koelsch, S. (2009). Neural substrates of processing syntax and semantics in music. In *Music that works: Contributions of biology, neurophysiology, psychology, sociology, medicine and musicology* (pp. 143–153). Springer Vienna. 10.1007/978-3-211-75121-3_9

[CR148] Kolinski, M. (1978). The structure of music: Diversification versus constraint. *Ethnomusicology*, *22*(2), 229–244. https://doi.org/10/dw5wzx

[CR149] Kolinsky, R., Lidji, P., Peretz, I., Besson, M., & Morais, J. (2009). Processing interactions between phonology and melody: Vowels sing but consonants speak. *Cognition*, *112*(1), 1–20. https://doi.org/10/bmvx62 10.1016/j.cognition.2009.02.014 19409537

[CR150] Korobova, A. G. (2007). *The theory of genres in the science of music: History and contemporaneity [Теория жанров в музыкальной науке: История и современность]*. Moscow State Tchaikovsky Conservatory.

[CR151] Korsakova-Kreyn, M. (2013). Proportions in Motion. In J.-L. Leroy (Ed.), *Topicality of Musical Universals / Actualité des Universaux musicaux* (pp. 6–11). Éditions des Archives Contemporaines.

[CR152] Krumhansl, C. L. (2002). Music: A Link Between Cognition and Emotion. *Current Directions in Psychological Science*, *11*, 45–50. https://doi.org/10/bcm3fn

[CR153] Kühl, O. (2011). The semiotic gesture. In E. King, & A. Gritten (Eds.), *New perspectives on music and gesture*. Routledge.

[CR154] Kwoun SJ (2009). An examination of cue redundancy theory in cross-cultural decoding of emotions in music. Journal of Music Therapy.

[CR155] Larson, S. (1997). The problem of prolongation in “tonal” music: Terminology, perception, and expressive meaning. *Journal of Music Theory*, *41*, 101. https://doi.org/10/fp2r2t

[CR156] Larson, S. (2012). *Musical forces: Motion, metaphor, and meaning in music*. Indiana University Press.

[CR157] Larson, S., & McAdams, S. (2004). Musical forces and melodic expectations: Comparing computer models and experimental results. *Music Perception*, *21*(4), 457–498. https://doi.org/10/cgh69p

[CR158] Larson, S., & Vanhandel, L. (2005). Measuring musical forces. *Music Perception*, *23*(2), 119–136. https://doi.org/10/ffw4zh

[CR159] Laukka P, Eerola T, Thingujam NS, Yamasaki T, Beller G (2013). Universal and culture-specific factors in the recognition and performance of musical affect expressions. Emotion.

[CR160] Le Bomin S, Lecointre G, Heyer E (2016). The evolution of musical diversity: The key role of vertical transmission. PloS one.

[CR161] Leach, H. M. (2003). Human domestication reconsidered. *Current Anthropology, 44*, 349–68. https://doi.org/10/b6rsxr

[CR601] Leisiö, T. 2002. On old-Lithuanian modalities: A hypothesis on five stylistic strata based on Proto-Indo-European pentatonic roots. In *Ethnic relations and musical folklore*, (pp. 22–51). Vilnius: Lietuvos Muzikos Akademija.

[CR162] Leman, M. (2009). Music, gesture, and the formation of embodied meaning. In M. Leman, & R. I. Godøy (Eds.), *Musical gestures* (pp. 138–165). Routledge.

[CR163] Leontyev AN, Cole M, Kipylova M (2009). The development of mind: Selected works of Aleksei Nikolaevich Leontyev.

[CR164] Leroy, S. A. G., Arpe, K., & Mikolajewicz, U. (2011). Vegetation context and climatic limits of the Early Pleistocene hominin dispersal in Europe. *Quaternary Science Reviews*, *30*(11–12), 1448–1463. https://doi.org/10/dkr9jc

[CR165] Levitin, D. J. (2019). *The world in six songs: How the musical brain created human nature* (2nd ed.). Penguin Books.

[CR166] Levitin, D. J., & Cook, P. R. (1996). Memory for musical tempo: Additional evidence that auditory memory is absolute. *Perception and Psychophysics*, *58*(6), 927–935. https://doi.org/10/dsvv7f 10.3758/bf03205494 8768187

[CR167] Lindblom, B. (1998). Systemic constraints and adaptive change in the formation of sound structure. In J. R. Hurford, M. Studdert-Kennedy, & C. Knight (Eds.), *Approaches to the evolution of language: Social and cognitive bases* (pp. 242–264). Cambridge University Press.

[CR168] Lischinsky JE, Lin D (2020). Neural mechanisms of aggression across species. Nature Neuroscience.

[CR169] Lisina, M. I. (1966). Development of the cognitive capacity in children during their first half a year of life [Развитие познавательной деятельности детей первого полугодия жизни]. In A. V. Zaporozhets & M. I. Lisina (Eds.), *Development of Perception in Early and Preschool Childhood. [Развитие восприятия в раннем и дошкольном детстве]* (pp. 16–48). Prosvesheniye.

[CR170] List, G. (1971). On the non-universality of musical perspectives. *Ethnomusicology*, *15*(3), 399. https://doi.org/10/ft4xbb

[CR171] List G (1984). Concerning the concept of the universal and music. The World of Music.

[CR172] Liszkowski, U., Brown, P., Callaghan, T., Takada, A., & de Vos, C. (2012). A prelinguistic gestural universal of human communication. *Cognitive Science*, *36*(4), 698–713. https://doi.org/10/f3xjjm 10.1111/j.1551-6709.2011.01228.x 22303868

[CR173] Lobanova, M. (2013). *Musical style and genre: History and modernity*. Routledge.

[CR174] Lomax A (1977). Universals in song. The World of Music.

[CR175] London, J. (2004). *Hearing in time: Psychological aspects of musical meter*. Oxford University Press.

[CR176] López-Cano, R. (2020). *La música cuenta. Retórica, narratividad, dramaturgia, cuerpo y afectos* ESMUC. http://rlopezcano.blogspot.com/2020/04/la-musica-cuenta.html

[CR177] Lord, K. A., Larson, G., Coppinger, R. P., & Karlsson, E. K. (2020). The history of farm foxes undermines the animal domestication syndrome. *Trends in Ecology and Evolution, 35*, 125–36. https://doi.org/10/ggfwhj 10.1016/j.tree.2019.10.011 31810775

[CR178] Lots, I. S., & Stone, L. (2008). Perception of musical consonance and dissonance: An outcome of neural synchronization. *Journal of the Royal Society Interface*, *5*(29), 1429–1434. https://doi.org/10/d9vp9s 10.1098/rsif.2008.0143 PMC2607353 18547910

[CR179] Lumaca M, Baggio G (2017). Cultural transmission and evolution of melodic structures in multi-generational signaling games. Artificial Life.

[CR180] Mabbett, M. (1990). Music and rhetoric: Style and communication in Western and non-Western musics. RMA Conference 7–9 April, 1989. *Early Music*, *XVIII*(2), 349. 10.1093/em/XVIII.2.349

[CR181] Maclarnon, A., & Hewitt, G. (2004). Increased breathing control: another factor in the evolution of human language. *Evolutionary Anthropology, 13*(5), 181–97. https://doi.org/10/bgpnct

[CR183] Malloch, S. (2000). Mothers and infants and communicative musicality. *Musicae Scientiae*, *3*(1 suppl), 29–57. https://doi.org/10/gg5vnm

[CR184] Malloch, S. (2004). An exploration of timbre analysis: The game of sound in two performances of *Jeux Vénitiens*. *Musicae Scientiae*, *8*(1), 53–81. https://doi.org/10/gmnfq8

[CR182] Malloch, S., & Trevarthen, C. (2009). *Communicative musicality: Exploring the basis of human companionship*. Oxford University Press.

[CR185] Mania, D., & Mania, U. (2004). The natural and socio-cultural environment of *Homo erectus* at Bilzingsleben, Germany. In C. Gamble (Ed.), *The hominid individual in context* (pp. 98–114). Routledge.

[CR186] Manser, M. B. (2010). The generation of functionally referential and motivational vocal signals in mammals. In S. M. Brudzynski (Ed.), *Handbook of Behavioral Neuroscience* (Vol. 19, pp. 477–486). Elsevier. 10.1016/B978-0-12-374593-4.00043-7

[CR187] Marx, A. B., & Burnham, S. G. (1997). *Musical form in the age of Beethoven: Selected writings on theory and method*. Cambridge University Press.

[CR188] Masataka, N. (2006). Preference for consonance over dissonance by hearing newborns of deaf parents and of hearing parents. *Developmental Science*, *9*(1), 46–50. https://doi.org/10/cjcjd3 10.1111/j.1467-7687.2005.00462.x 16445395

[CR189] Mattheson J, Harriss EC, Harriss EC (1981). Johann Mattheson’s Der vollkommene Capellmeister: A revised translation with critical commentary.

[CR190] Mazel, L. (1952). *On melody [О мелодии]*. Gos Muz Izdat [State Musical Publishing].

[CR191] McAdams S (1989). Psychological constraints on form-bearing dimensions in music. Contemporary Music Review.

[CR192] McAuley, J. D. (2010). Tempo and rhythm. In J. M. Riess, R. Fay, & A. Popper (Eds.), *Springer Handbook of Auditory Research* (pp. 165–199). Springer. 10.1007/978-1-4419-6114-3_6

[CR193] McBride, J. M., Passmore, S., & Tlusty, T. (2022). Convergent evolution in a large cross-cultural database of musical scales. arXiv. 10.48550/arXiv.2108.00842 10.1371/journal.pone.0284851 PMC10718441 38091315

[CR194] McPherson MJ, Dolan SE, Durango A, Ossandon T, Valdés J, Undurraga EA, Jacoby N, Godoy RA, McDermott JH (2020). Perceptual fusion of musical notes by native Amazonians suggests universal representations of musical intervals. Nature Communications.

[CR195] Mehr SA, Krasnow MM, Bryant GA, Hagen EH (2021). Origins of music in credible signaling. Behavioral and Brain Sciences.

[CR196] Meier, B. (1990). Rhetorical aspects of the Renaissance modes. *Journal of the Royal Musical Association*, *115*(2), 182–190. https://doi.org/10/bfkh4f

[CR197] Mellars P, Mellars P, Gibson KR (1996). Symbolism, language, and the neanderthal mind. Modelling the early human mind.

[CR198] Meneganzin A, Bernardi M (2023). Were neanderthals and *Homo sapiens* “good species”?. Quaternary Science Reviews.

[CR199] Merker B (2000). Synchronous chorusing and human origins. Musicae Scientiae.

[CR200] Messner, G. F. (2006). Multipart vocal tradition in eastern Flores (Indonesia), Bulgaria and Manus Province. In R. Tsurtsumia (Ed.), *Proceedings: The Third International Symposium on Traditional Polyphony: 25–29 September, 2006, Tbilisi, Georgia*.

[CR201] Messner, G. F. (2013). *Do they sound like bells or like howling wolves? Interferential diaphony in Bistritsa: An investigation into a multi-part singing tradition in a middle-western bulgarian village*. Peter Lang.

[CR202] Miller, D. G. (2000). *Registers in singing: Empirical and systematic studies in the theory of the singing voice*. University of Groningen.

[CR203] Mithen, S. J. (2005). *The singing neanderthals: The origins of music, language, mind, and body*. Harvard University Press.

[CR204] Mohn, C., Argstatter, H., & Wilker, F. W. (2010). Perception of six basic emotions in music. *Psychology of Music*, *39*(4), 503–517. https://doi.org/10/b8d3td

[CR205] Monaghan, P., Shillcock, R. C., Christiansen, M. H., & Kirby, S. (2014). How arbitrary is language? *Philosophical Transactions of the Royal Society of London B: Biological Sciences*, *369*(1651), 20130299. https://doi.org/10/f6p6g7 10.1098/rstb.2013.0299 PMC4123678 25092667

[CR206] Monahan, C. B. (1993). Parallels between pitch and time and how they go together. In T. J. Tighe, & W. J. Dowling (Eds.), *Psychology and music: The understanding of melody and rhythm* (pp. 121–154). Erlbaum.

[CR207] Monelle, R. (2006). *The musical topic: Hunt, military and pastoral*. Indiana University Press.

[CR208] Montagu J (2004). How old is music?. The Galpin Society Journal.

[CR209] Moreno S, Farzan F (2015). Music training and inhibitory control: A multidimensional model. Annals of the New York Academy of Sciences.

[CR210] Morley I (2013). The prehistory of music: Human evolution, archaeology, and the origins of musicality.

[CR211] Moyer, B. P. V. (1969). *Concepts of musical form in the nineteenth century with special reference to A. B. Marx and the sonata form*. Stanford University.

[CR212] Mukhina, T. K., & Lisina, M. I. (1966). The dependency of age and individual achievements in discrimination of pitch from the type of activity in preschool age children [Зависимость возрастных и индивидуальных показателей звуковысотного дифференцирования от характера деятельности детей в пред. In A. V. Zaporozhets & M. I. Lisina (Eds.), *Development of Perception in Early and Preschool Childhood [Развитие восприятия в раннем и дошкольном детстве]* (pp. 49–73). Prosvesheniye.

[CR213] Murphy, J. J. (1981). *Rhetoric in the Middle Ages: A history of rhetorical theory from Saint Augustine to the Renaissance*. University of California Press.

[CR214] Naguib, M., & Riebel, K. (2014). Singing in space and time: The biology of birdsong. In G. Witzany (Ed.), *Biocommunication of animals* (pp. 233–247). Springer Netherlands. 10.1007/978-94-007-7414-8_13

[CR215] Nattiez, J. J. (2012). Is the search for universals incompatible with the study of cultural specificity? *Human and Social Studies*, *1*(1), 67–94. https://doi.org/10/gmnfnk

[CR216] Nazaikinsky, Y. V. (1977). Interconnection between the intervallic-based and degree-based representation of music in the development of a musical ear [Взаимосвязи интервальных и ступеневых представлений в развитии музыкального слуха]. In A. Agazhanov (Ed.), *Development of musical hearing [Воспитание музыкального слуха]* (Vol. 1, pp. 25–77). Muzyka.

[CR217] Nazaikinsky, Y. V. (1982). *The logic of musical composition [Логика музыкальной композиции]*. Muzyka.

[CR218] Nazaikinsky, Y. V. (1988). *The sonic world of music [Звуковой мир музыки]*. Muzyka.

[CR219] Nazaikinsky, Y. V. (2013). *Style and genre in music [Стиль и жанр в музыке]*. Tbilisi State Conservatoire.

[CR220] Nettl, B. (2000). An ethnomusicologist contemplates universals in musical sound and musical culture. In N. L. Wallin, B. Merker, & S. Brown (Eds.), *The origins of music* (pp. 463–472). MIT Press.

[CR221] Nettl, B. (2005). *The study of ethnomusicology: Thirty-one issues and concepts*. University of Illinois Press.

[CR222] Nettl, B. (2010). *Nettl’s elephant: On the history of ethnomusicology*. University of Illinois Press.

[CR223] Neubauer S, Hublin JJ, Gunz P (2018). The evolution of modern human brain shape. Science Advances.

[CR224] Nieminen, S., Istók, E., Brattico, E., Tervaniemi, M., & Huotilainen, M. (2011). The development of aesthetic responses to music and their underlying neural and psychological mechanisms. *Cortex*, *47*(9), 1138–1146. https://doi.org/10/cczqbc 10.1016/j.cortex.2011.05.008 21665202

[CR225] Nikolsky, A. (2015a). ¿Cómo funciona la emoción musical? [How can emotion be the meaning of a musical work?]. In Teresa. Cascudo (Ed.), *Música y cuerpo: Estudios musicológicos* (pp. 241–262). Calanda Ediciones Musicales. 10.13140/RG.2.1.2737.0008

[CR226] Nikolsky, A. (2015b). Evolution of tonal organization in music mirrors symbolic representation of perceptual reality. Part 1: Prehistoric. *Frontiers in Psychology*, *6*(1405). https://doi.org/10/f7wvp8 10.3389/fpsyg.2015.01405 PMC4607869 26528193

[CR227] Nikolsky, A. (2016a). The commonalities between melodic line, geometric line, and environmental topography in traditional cultures of northern Siberia: “Landscape aesthetics” as a model of musical genesis. *Frontiers in Psychology*, *7*. https://doi.org/10/gmnfz2

[CR228] Nikolsky, A. (2016b). Evolution of tonal organization in music optimizes neural mechanisms in symbolic encoding of perceptual reality. Part 2: Ancient to seventeenth century. *Frontiers in Psychology*, *7*, 211. https://doi.org/10/gmdd4n 10.3389/fpsyg.2016.00211 PMC4813086 27065893

[CR229] Nikolsky, A. (2016c). Chromatic alteration as expression of aesthetic emotion: from the ancient doctrine of ethos to the emergence of the notion of musical error. *Frontiers in Psychology*, *7*. https://doi.org/10/gmnfz3

[CR230] Nikolsky, A. (2018). General typology of music texture in the evolutionary earliest forms of music. Commentary on ‘The “Musilanguage” model of language evolution. *Frontiers in Psychology*, *9*, 75. https://doi.org/10/gmnfrt 10.3389/fpsyg.2018.00075 PMC5834477 29536987

[CR231] Nikolsky, A. (2020a). Emergence of the distinction between “verbal” and “musical” in early childhood development. In N. Masataka (Ed.), *the origins of language revisited: differentiation from music and the emergence of neurodiversity and autism* (pp. 139–216). Springer Nature. 10.1007/978-981-15-4250-3_7

[CR232] Nikolsky, A. (2020b). The pastoral origin of semiotically functional tonal organization of music. *Frontiers in Psychology*, *11*(June), 1358. https://doi.org/10/gmnfx2 10.3389/fpsyg.2020.01358 PMC7396614 32848961

[CR233] Nikolsky, A. (2022). Music cognition from birth to adolescence: A structuralist approach. [Monograph]. https://psyarxiv.com. 10.31234/osf.io/dkpsj

[CR235] Nikolsky A, Benítez-Burraco A (2022). Human aggression and music evolution: A model. PsyArXiv.

[CR234] Nikolsky, A., Alekseyev, E. Y., Alekseev, I. Y., & Dyakonova, V. E. (2020). The overlooked tradition of “personal music” and its place in the evolution of music. *Frontiers in Psychology, 10*, 3051. https://doi.org/10/gmnfww 10.3389/fpsyg.2019.03051 PMC7040865 32132941

[CR236] Nketia JHK (1984). Universal perspectives in ethnomusicology. The World of Music.

[CR238] Pamjav H, Juhász Z, Zalán A, Németh E, Damdin B (2012). A comparative phylogenetic study of genetics and folk music. Molecular Genetics and Genomics.

[CR239] Panksepp, J., & Trevarthen, C. (2009). The neuroscience of emotion in music. In S. Malloch, & C. Trevarthen (Eds.), *Communicative musicality: Exploring the basis of human companionship* (pp. 105–146). Oxford University Press.

[CR240] Parncutt, R. (2016). Prenatal development and the phylogeny and ontogeny of musical behavior. In S. Hallam, I. Cross, & M. Thaut (Eds.), *Oxford handbook of music psychology* (pp. 371–386). Oxford University Press. 10.1093/oxfordhb/9780198722946.013.11.

[CR241] Patel AD (2003). Rhythm in language and music: Parallels and differences. Annals of the New York Academy of Sciences.

[CR242] Pereira, A. S., Kavanagh, E., Hobaiter, C., Slocombe, K. E., & Lameira, A. R. (2020). Chimpanzee lip-smacks confirm primate continuity for speech-rhythm evolution. *Biology Letters, 16*, 20200232. https://doi.org/10/ggxrjd 10.1098/rsbl.2020.0232 PMC7280036 32453963

[CR243] Peretz, I. (2013). Towards a neurobiology of musical emotions. In P. N. Juslin & J. A. Sloboda (Eds.), *Handbook of Music and Emotion: Theory, Research, Applications* (pp. 99–126). Oxford Uuniversity Press. 10.1093/acprof:oso/9780199230143.003.0005

[CR244] Perlovsky, L. (2012). Cognitive function, origin, and evolution of musical emotions. *Musicae Scientiae*, *16*(2), 185–199. https://doi.org/10/gfsdgr

[CR245] Perlovsky, L. (2014). The cognitive function of music, part II. *Interdisciplinary Science Reviews*, *39*(2), 162–186. https://doi.org/10/gmnfjj

[CR246] Perlovsky L (2017). Music, passion, and cognitive function.

[CR247] Perrone-Capano C, Volpicelli F, di Porzio U (2017). Biological bases of human musicality. Reviews in the Neurosciences.

[CR248] Pisor, A. C., & Surbeck, M. (2019). The evolution of intergroup tolerance in nonhuman primates and humans. *Evolutionary Anthropology, 28*, 210–23. https://doi.org/10/gmnf2d 10.1002/evan.21793 31386248

[CR249] Plavcan, J. M. (2012). Sexual size dimorphism, canine dimorphism, and male-male competition in primates: Where do humans fit in? *Human Nature, 23*, 45–67. https://doi.org/10/ggwbpr 10.1007/s12110-012-9130-3 22388772

[CR250] Potter, D. D., Fenwick, M., Abecasis, D., & Brochard, R. (2009). Perceiving rhythm where none exists: Event-related potential (ERP) correlates of subjective accenting. *Cortex*, *45*(1), 103–109. https://doi.org/10/d3jcrd 10.1016/j.cortex.2008.01.004 19027894

[CR251] Powers, H. S. (1980). Language models and musical analysis. *Ethnomusicology*, *24*(1), 1–60. https://doi.org/10/cjr8z7

[CR252] Pressing J (1983). Cognitive isomorphisms in pitch and rhythm in world music: West Africa, the Balkans, Thailand, and western tonality. Studies in Music.

[CR253] Progovac, L., & Benítez-Burraco, A. (2019). From physical aggression to verbal behavior: Language evolution and self-domestication feedback loop. *Frontiers in Psychology, 10*, 2807. https://doi.org/10/gnbvgg 10.3389/fpsyg.2019.02807 PMC6930236 31920850

[CR254] Rags, Y. N. (1980). *Garbuzov N.A. - Musician, researcher and pedagogue [Гарбузов Н.А. - Музыкант, исследователь, педагог]*. Muzyka.

[CR255] Ravignani, A., Delgado, T., & Kirby, S. (2016). Musical evolution in the lab exhibits rhythmic universals. *Nature Human Behaviour*, *1*(1), 1–7. https://doi.org/10/gfr9gz

[CR256] Reigado J, Rocha A, Rodrigues H (2011). Vocalizations of infants (9–11 months old) in response to musical and linguistic stimuli. International Journal of Music Education.

[CR257] Repina, T. A. (1966). On the problem of the mechanisms of objectivitization of child’s pitch distinctions [К вопросу о механизмах явления «опредмечивания» в звуковысотном различении ребенка]. In A. V. Zaporozhets & M. I. Lisina (Eds.), *Development of Perception in Early and Preschool Childhood. [Развитие восприятия в раннем и дошкольном детстве]* (pp. 98–141). Prosvesheniye.

[CR258] Reybrouck M, Vuust P, Brattico E (2018). Brain connectivity networks and the aesthetic experience of music. Brain Sciences.

[CR259] Rink, J. (1989). Conference report: “Music and Rhetoric: Style and Communication in Western and Non-Western Musics.” Royal Musical Association, Wellington Hall, London, 7–9 April 1989. *Music Analysis*, *8*(3), 359–364. https://www.jstor.org/stable/854298

[CR260] Rodman, R., & Rodman, R. W. (2010). *Tuning in: American narrative television music*. Oxford University Press.

[CR261] Rohrmeier, M., & Rebuschat, P. (2012). Implicit learning and acquisition of music. *Topics in Cognitive Science, 4*, 525–53. https://doi.org/10/gfkfd5 10.1111/j.1756-8765.2012.01223.x 23060126

[CR262] Rohrmeier M, Zuidema W, Wiggins GA, Scharff C (2015). Principles of structure building in music, language and animal song. Philosophical Transactions of the Royal Society B: Biological Sciences.

[CR263] Rosenblatt A, Leroi I (2000). Neuropsychiatry of Huntington’s disease and other basal ganglia disorders. Psychosomatics.

[CR264] Rothfarb, L. A. (1988). *Ernst Kurth as theorist and analyst*. University of Pennsylvania Press.

[CR265] Rzeszutek, T., Savage, P. E., & Brown, S. (2012). The structure of cross-cultural musical diversity. *Proceedings of the Royal Society B: Biological Sciences*, *279*(1733), 1606–1612. 10.1098/rspb.2011.1750 10.1098/rspb.2011.1750 PMC3282333 22072606

[CR266] Salimpoor, V. N., & Zatorre, R. J. (2013). Neural interactions that give rise to musical pleasure. *Psychology of Aesthetics, Creativity, and the Arts*, *7*(1), 62–75. https://doi.org/10/f4q7cj

[CR267] Samson, J. (2001). Genre. In S. Sadie, & J. Tyrrel (Eds.), *The new Grove dictionary of music and musicians*. Macmillan. 10.1093/gmo/9781561592630.article.40599.

[CR268] Samuels, R. (2004). *Mahler’s sixth symphony: A study in musical semiotics*. Cambridge University Press.

[CR270] Sánchez-Villagra, M. R., & van Schaik, C. P. (2019). Evaluating the self-domestication hypothesis of human evolution. *Evolutionary Anthropology, 28*, 133–43. https://doi.org/10/gh3x99 10.1002/evan.21777 30938920

[CR269] Sánchez-Villagra, M. R., Geiger, M., & Schneider, R. A. (2016). The taming of the neural crest: a developmental perspective on the origins of morphological covariation in domesticated mammals. *Royal Society Open Science, 3*, 160107. https://doi.org/10/gcx732 10.1098/rsos.160107 PMC4929905 27429770

[CR271] Sandler, W., Meir, I., Padden, C., & Aronoff, M. (2005). The emergence of grammar: Systematic structure in a new language. *Proceedings of the National Academy of Sciences, 102*, 2661–5. https://doi.org/10/bbpkzw 10.1073/pnas.0405448102 PMC548320 15699343

[CR272] Savage CR (1997). Neuropsychology of subcortical dementias. The Psychiatric Clinics of North America.

[CR274] Savage, P. E., Brown, S., Sakai, E., & Currie, T. E. (2015). Statistical universals reveal the structures and functions of human music. *Proceedings of the National Academy of Sciences*, *112*(29), 8987–8992. https://doi.org/10/f7j74k 10.1073/pnas.1414495112 PMC4517223 26124105

[CR273] Savage PE, Loui P, Tarr B, Schachner A, Glowacki L, Mithen S (2020). Music as a coevolved system for social bonding. Behavioral and Brain Sciences.

[CR275] Scerri EML, Chikhi L, Thomas MG (2019). Beyond multiregional and simple out-of-Africa models of human evolution. Nature Ecology & Evolution.

[CR276] Schäfer, T., & Sedlmeier, P. (2009). From the functions of music to music preference. *Psychology of Music*, *37*(3), 279–300. https://doi.org/10/ch3pkt

[CR277] Schäfer, T., Tipandjan, A., & Sedlmeier, P. (2012). The functions of music and their relationship to music preference in India and Germany. *International Journal of Psychology*, *47*(5), 370–380. https://doi.org/10/gms2vc 10.1080/00207594.2012.688133 22721000

[CR278] Schellenberg, G., & Trehub, S. E. (1996). Natural musical intervals: Evidence from infant listeners. *Psychological Science*, *7*(5), 272–277. https://doi.org/10/d8nq4n

[CR279] Schiavio A, van der Schyff D, Cespedes-Guevara J, Reybrouck M (2017). Enacting musical emotions, sense-making, dynamic systems, and the embodied mind. Phenomenology and the Cognitive Sciences.

[CR280] Schubert, E. (2009). The fundamental function of music. *Musicae Scientiae*, *13*(2_suppl), 63–81. https://doi.org/10/bsjp6j

[CR281] Schulkin, J. (2013). *Reflections on the musical mind: An evolutionary perspective*. Princeton University Press.

[CR282] Schwartz, D. A., Howe, C. Q., & Purves, D. (2003). The statistical structure of human speech sounds predicts musical universals. *The Journal of Neuroscience*, *23*(18), 7160–7168. https://doi.org/10/ggc8gg 10.1523/JNEUROSCI.23-18-07160.2003 PMC6740660 12904476

[CR283] Scott LM (1990). Understanding jingles and needledrop: A rhetorical approach to music in advertising. Journal of Consumer Research.

[CR284] Sethares, W. A. (2005). *Tuning, timbre, spectrum, scale*. Springer Science & Business Media.

[CR285] Shea, B. T. (1989). Heterochrony in human evolution: The case for neoteny reconsidered. *American Journal of Physical Anthropology, 32*, 69–101. https://doi.org/10/bj5rr6

[CR286] Sheikin, Y. I. (2002). *The history of music culture of Siberian ethnicities: A comparative historical investigation [История музыкальной культуры народов Сибири: Сравнительно-историческое исследование]*. Eastern Literature, Russian Academy of Science.

[CR287] Shepard RN (2010). One cognitive psychologist’s quest for the structural grounds of music cognition. Empirical Musicology Review.

[CR288] Sievers, B., Polansky, L., Casey, M., & Wheatley, T. (2013). Music and movement share a dynamic structure that supports universal expressions of emotion. *Proceedings of the National Academy of Sciences*, *110*(1), 70–75. https://doi.org/10/f4kn7b 10.1073/pnas.1209023110 PMC3538264 23248314

[CR289] Slater, P. (2001). Birdsong repertoires: Their origin and use. In S. Brown, B. Merker, & N. L. Wallin (Eds.), *The origins of music* (pp. 49–63). MIT Press.

[CR290] Slater, P. (2011). Bird song and language. In K. R. Gibson, & M. Tallerman (Eds.), *The Oxford Handbook of Language Evolution*. Oxford University Press. 10.1093/oxfordhb/9780199541119.013.0008.

[CR291] Slevc LR (2012). Language and music: Sound, structure, and meaning. Wiley Interdisciplinary Reviews: Cognitive Science.

[CR292] Smith AL (1971). Markings of an african concept of rhetoric. Today’s Speech.

[CR294] Smith LD, Williams RN (1999). Children’s artistic responses to musical intervals. The American Journal of Psychology.

[CR293] Smith K, Wonnacott E (2010). Eliminating unpredictable variation through iterated learning. Cognition.

[CR295] Snowdon CT (2021). Animal signals, music and emotional well-being. Animals.

[CR296] Sokhor, A. (1968). *The aesthetic nature of genre in music [Эстетическая природа жанра в музыке]*. Muzyka.

[CR297] Sokhor, A. (1971). Theory of musical genres: Goals and perspectives [Теория музыкальных жанров: Задачи и перспективы]. In A. Sokhor & Y. Kholopov (Eds.), *Theoretical problems of musical forms and genres [Теоретические проблемы музыкальных форм и жанров]* (pp. 292–309). Muzyka.

[CR298] Somel, M., Franz, H., Yan, Z., Lorenc, A., Guo, S., Giger, T. (2009). Transcriptional neoteny in the human brain. *Proceedings of the National Academy of Sciences, 106*, 5743–8. https://doi.org/10/dgw9kf 10.1073/pnas.0900544106 PMC2659716 19307592

[CR299] Spikins, P., French, J. C., John-Wood, S., & Dytham, C. (2021). Theoretical and methodological approaches to ecological changes, social behaviour and human intergroup tolerance 300,000 to 30,000 BP. *Journal of Archaeological Method and Theory, 28*, 53–75. https://doi.org/10/gmnf2g 10.1007/s10816-020-09503-5 PMC7891228 33679119

[CR300] Stefanics, G., Háden, G. P., Sziller, I., Balázs, L., Beke, A., & Winkler, I. (2009). Newborn infants process pitch intervals. *Clinical Neurophysiology*, *120*(2), 304–308. https://doi.org/10/cfxvqw 10.1016/j.clinph.2008.11.020 19131275

[CR301] Stefanija L (2007). Functions of music: A Survey of Research Vocabularies. Muzikos Funkcijos: Tyrimų Terminologijos Apžvalga (Lithuanian).

[CR302] Stevens, C., & Byron, T. P. (2009). Universals in music processing. *Oxford handbook of music psychology* (pp. 14–23). Oxford University Press.

[CR303] Stewart L, von Kriegstein K, Warren JD, Griffiths TD (2006). Music and the brain: Disorders of musical listening. Brain: A Journal of Neurology.

[CR304] Stringer, C. (2016). The origin and evolution of *Homo sapiens*. *Philosophical Transactions of the Royal Society B: Biological Sciences, 371*, 20150237. https://doi.org/10/gfsqs5 10.1098/rstb.2015.0237 PMC4920294 27298468

[CR305] Studdert-Kennedy, M. (2011). The emergence of phonetic form. In K. R. Gibson, & M. Tallerman (Eds.), *The Oxford Handbook of Language Evolution* (pp. 417–422). Oxford University Press. 10.1093/oxfordhb/9780199541119.013.0045.

[CR306] Sun Y, Lu X, Ho HT, Johnson BW, Sammler D, Thompson WF (2018). Syntactic processing in music and language: Parallel abnormalities observed in congenital amusia. NeuroImage Clinical.

[CR307] Supičič I (1983). Aesthetics of music—particularity and universality. The World of Music.

[CR308] Tagg, P. (2012). *Music’s meaning: A modern musicology for non-musos*. Mass Media’s Scholar’s Press.

[CR309] Tallerman, M. (2013). Join the dots: A musical interlude in the evolution of language? *Journal of Linguistics*, *49*(02), 455–487. https://doi.org/10/gmnfn5

[CR310] Tallmadge, W. H. (1984). Folk organum: A study of origins. *American Music, 2*(3), 47–65. https://doi.org/10/drqvrr

[CR311] Tamariz, M., & Kirby, S. (2016). The cultural evolution of language. *Current Opinion in Psychology, 8*, 37–43. https://doi.org/10/ggjk28 10.1016/j.copsyc.2015.09.003 29506800

[CR312] Tamm, M. (2019). Introduction: Juri Lotman’s semiotic theory of history and cultural memory. In M. Tamm (Ed.), *Juri Lotman—culture, memory and history: Essays in cultural semiotics* (pp. 1–26). Springer. 10.1007/978-3-030-14710-5_1

[CR313] Tarasti, E. (1998). From aesthetics to ethics: Semiotic observations on the moral aspects of art, especially music. In J. Jadacki & W. Strawińsky (Eds.), *In the world of signs* (pp. 363–373). Brill. 10.1163/9789004457621_039

[CR314] Teichmann M, Rosso C, Martini JB, Bloch I, Brugières P, Duffau H, Lehéricy S, Bachoud-Lévi AC (2015). A cortical-subcortical syntax pathway linking Broca’s area and the striatum. Human Brain Mapping.

[CR315] Temperley D (2009). In defense of introspectionism: A response to DeBellis. Music Perception.

[CR316] Tenney, J. (1988). *A history of consonance and dissonance*. Excelsior.

[CR317] Teplov, B. (1947). *The psychology of musical abilities [Психология музыкальных способностей]*. Academy of Pedagogical Sciences of Russia.

[CR318] Terhardt E (1974). On the perception of periodic sound fluctuations (roughness). Acustica.

[CR319] Terhardt, E. (1974b). Pitch, consonance, and harmony. *The Journal of the Acoustical Society of America*, *55*(5), 1061–1069. https://doi.org/10/fks3b7 10.1121/1.1914648 4833699

[CR320] Terhardt, E. (1984). The concept of musical consonance: A link between music and psychoacoustics. *Music Perception: An Interdisciplinary Journal*, *1*(c), 276–295. https://doi.org/10/gmnfhv

[CR321] Theodosopoulou I (2019). Semiotic approaches to “traditional music,” musical/poetic structures, and ethnographic research. Semiotica.

[CR322] Thomas J, Kirby S (2018). Self-domestication and the evolution of language. Biology& Philosophy.

[CR323] Tillmann B, Albouy P, Caclin A (2015). Congenital amusias. Handbook of clinical neurology.

[CR324] Tiulin, Y. N. (1937). *The doctrine of harmony [Учение о гармонии]*. Muzyka.

[CR325] Tomlinson G (2015). A million years of music: The emergence of human modernity.

[CR326] Trainor, L. J. (2010). The emotional origins of music. *Physics of Life Reviews*, *7*(1), 44–45. https://doi.org/10/d7h7kx 10.1016/j.plrev.2010.01.010 20374924

[CR327] Trainor, L. J., Tsang, C. D., & Cheung, V. H. W. (2002). Preference for sensory consonance in 2- and 4-month-old infants. *Music Perception*, *20*(2), 187–194. https://doi.org/10/fdbrvb

[CR328] Trainor LJ, Wu L, Tsang CD (2004). Long-term memory for music: Infants remember tempo and timbre. Developmental Science.

[CR329] Tramo, M., Cariani, P., Delgutte, B., & Braida, L. D. (2001). Neurobiological foundations for the theory of harmony in western tonal music. *Annals of the New York Academy of Sciences*, *930*(1), 92–116. https://doi.org/10/cb5g5d 10.1111/j.1749-6632.2001.tb05727.x 11458869

[CR330] Trehub, S. E., Unyk, A. M., & Trainor, L. J. (1993). Maternal singing in cross-cultural perspective. *Infant Behavior and Development*, *16*(3), 285–295. https://doi.org/10/fgtqfs

[CR331] Trevarthen, C. (2002). Origins of musical identity: Evidence from infancy for musical social awareness. In R. MacDonald, D. J. Hargreaves, & D. Miell (Eds.), *Musical identities* (pp. 21–38). Oxford University Press.

[CR332] Trevarthen, C. (2009). Human biochronology: On the source and functions of “musicality. In R. Haas, & V. Brandes (Eds.), *Music that works: Contributions of biology, neurophysiology, psychology, sociology, medicine and musicology* (pp. 221–265). Springer.

[CR333] Tsaryova, Y. M. (1976). Music genre [Музыкальный жанр]. In Y. V. Keldysh (Ed.), *Musical Encyclopedia [Музыкальная энциклопедия]* (Vol. 2, pp. 383–388). Soviet Encyclopedia [Советская энциклопедия].

[CR334] Tsukkerman, V. (1964). *Musical genres and the basics of musical form [Музыкальные жанры и основы музыкальной формы]*. Muzyka.

[CR335] van der Veer, R., & Valsiner, J. (1991). *Understanding Vygotsky: A quest for synthesis*. Blackwell.

[CR336] van Dijck J (2006). Record and hold: Popular music between personal and collective memory. Critical Studies in Media Communication.

[CR337] van Goethem A, Sloboda J (2011). The functions of music for affect regulation. Musicae Scientiae.

[CR237] van Noorden, L. (1975). *Temporal coherence in the perception of tone sequences*. PhD thesis, Institute for Perceptual Research, Eindhoven.

[CR338] van Noorden L, Moelants D (1999). Resonance in the perception of musical pulse. Journal of New Music Research.

[CR339] Verhoef T (2012). The origins of duality of patterning in artificial whistled languages. Language & Cognition.

[CR340] Verhoef, T., & Ravignani, A. (2021). Melodic universals emerge or are sustained through cultural evolution. *Frontiers in Psychology*, *12*, 668300. https://doi.org/10/gmw3kq 10.3389/fpsyg.2021.668300 PMC8365168 34408694

[CR341] Verhoef, T., Kirby, S., & de Boer, B. (2014). Emergence of combinatorial structure and economy through iterated learning with continuous acoustic signals. *Journal of Phonetics, 43*, 57–68. https://doi.org/10/gcpb5f

[CR342] Vickers B (1984). Figures of rhetoric/Figures of music?. Rhetorica: A Journal of the History of Rhetoric.

[CR343] Vuust P, Roepstorff A (2008). Listen up! Polyrhythms in brain and music. Cognitive Semiotics.

[CR344] Vuust P, Heggli OA, Friston KJ, Kringelbach ML (2022). Music in the brain. Nature Reviews Neuroscience.

[CR345] Vygotsky, L. S. (1987). In R. W. Rieber, & A. S. Carton (Eds.), *The collected works of L.S. Vygotsky, vol. 5: Child psychology*. Plenum Press.

[CR346] Vygotsky, L. S. (2013). Studies on the history of behavior: Ape, primitive, and child. In V. I. Golod, & J. E. Knox (Eds.), *Studies on the history of behavior*. Psychology Press. 10.4324/9780203772683.

[CR347] Wallin NL, Merker B, Brown S (2000). The origins of music.

[CR348] Watanabe, S. (2008). How animals perceive music: Comparative study of discriminative and reinforcing properties of music for infrahuman animals. In *CARLS series of advanced study of logic and sensibility* (Vol. 2, pp. 5–16). Centre for Advanced Research on Logic and Sensibility (CARLS). Global Centers of Excellence Program, Keio University. https://www.semanticscholar.org/paper/Title-How-animals-perceive-music-%3A-comparative-of-Watanabe/ec235ad723ba688cccda490079df593cea9a3737

[CR349] Watanabe, S., Uozumi, M., & Tanaka, N. (2005). Discrimination of consonance and dissonance in Java sparrows. *Behavioural Processes*, 70, 203–208. 10.1016/j.beproc.2005.06.001 10.1016/j.beproc.2005.06.001 16043306

[CR350] West, M. L. (1992). *Ancient greek music*. Oxford University Press.

[CR351] Wilkins, A. S., Wrangham, R. W., & Fitch, W. T. (2014). The “domestication syndrome” in mammals: a unified explanation based on neural crest cell behavior and genetics. *Genetics, 197*, 795–808. https://doi.org/10/f6bjcb 10.1534/genetics.114.165423 PMC4096361 25024034

[CR352] Will, U. (2004). Oral memory in Australian Aboriginal song performance and the Parry-Kirk debate: A cognitive ethnomusicological perspective. In E. Hickmann & R. Eichmann (Eds.), *Music-Archaeological sources: Finds, oral transmission, written evidence* (pp. 161–179). Papers from the 3rd Symposium of the International Study Group on Music Archaeology, June 2002.

[CR353] Wong PCM, Roy AK, Hellmuth Margulis E (2009). Bimusicalism: The implicit dual enculturation of cognitive and affective systems. Music Perception.

[CR354] Yurdum, L., Singh, M., Glowacki, L., Vardy, T., Atkinson, Q., Hilton, C. B., Sauter, D., Krasnow, M., & Mehr, S. (2022). Cultural invariance in musical communication. In J. Culbertson & A. Perfors (Eds.), *Proceedings of the Annual Meeting of the Cognitive Science Society* (Vol. 44, pp. 326–333). Cognitive Science Society. https://escholarship.org/uc/item/7hc3762n

[CR355] Zakharova, O. I. (1983). *Rhetoric and Western European music XVII-the first half of the XVIII century: Principles, methods [Риторика и западноевропейская музыка XVII – первой половины XVIII века: Принципы, приемы]*. Muzyka. https://philpapers.org/rec/ZAKRIZ

[CR356] Zaporozhets, A. V. (1985). *Selected Works on Psychology [Избранные психологические труды]* (V. V. Davydova & V. P. Zinchenko, Eds.; Vol. 1). Pedagogika.

[CR357] Zarate, J. M., Wood, S., & Zatorre, R. J. (2010). Neural networks involved in voluntary and involuntary vocal pitch regulation in experienced singers. *Neuropsychologia, 48*(2), 607–18. https://doi.org/10/dzt2nj 10.1016/j.neuropsychologia.2009.10.025 19896958

[CR358] Zentner, M., & Kagan, J. (1998). Infants’ perception of consonance and dissonance in music. *Infant Behavior and Development*, *21*(3), 483–492. https://doi.org/10/c6mtnp

[CR359] Zgaljardic DJ, Borod JC, Foldi NS, Mattis P (2003). A review of the cognitive and behavioral sequelae of Parkinson’s disease: Relationship to frontostriatal circuitry. Cognitive and Behavioral Neurology.

[CR360] Zollikofer, C. P. E., & Ponce de León, M. S. (2010). The evolution of hominin ontogenies. *Seminars in Cell & Developmental Biology, 21*, 441–52. https://doi.org/10/bxxjhp 10.1016/j.semcdb.2009.10.012 19900572

[CR361] Zuberbühler, K. (2017). The primate roots of human language. In R. M. Quam, M. Rosa, & J. L. Arsuaga (Eds.), *Primate hearing and communication* (pp. 175–200). Springer. 10.1007/978-3-319-59478-1_7

[CR362] Zubrow EBW, Blake EC, Scarre C, Lawson G (2006). The origin of music and rhythm. Archaeoacoustics.

[CR363] Zuidema, W., & de Boer, B. (2009). The evolution of combinatorial phonology. *Journal of Phonetics*, *37*(2), 125–144. https://doi.org/10/c5xx45

