# Supplementary material for: The (Co)Evolution of Language and Music Under Human Self-Domestication
Source: Hum Nat. 2023 Apr 25;34(2):229–75. doi: 10.1007/s12110-023-09447-1 (PMC10354115; doi:10.1007/s12110-023-09447-1)
Supplement: Supplementary file 1 — Supplementary Material 1 [file 12110_2023_9447_MOESM1_ESM.pdf]

Electronic Supplementary Material (ESM) for

The (Co)Evolution of Language and Music under Human Self-Domestication

Antonio Benítez-Burraco* and Aleksey Nikolsky

e-mail: abenitez8@us.es

Published in *Human Nature* 34(2), 2023, https://doi.org/10.1007/s12110-023-09447-1

# Key musicological terms for research on the evolution of music

This document contains definitions and brief explanations of the musical terms that are most important for studying the evolution of music and comparative analysis of different music systems. The explanations are aimed at those researchers who do not have a strong background in music theory. All the terms are grouped into a few sections marked by headings. The musicological information and psychoacoustic information are combined to identify the main physical and perceptual substrates of each of the defined musicological concepts. The key references are provided for those interested to find out more.

## Contents

Principal aspects of expression in music ........ 1
Elementary building blocks of music ........ 6
Means of integration of musical elements ........ 7
Complexity of tonal organization ........ 16
Melodic typology ........ 24
Textural organization ........ 29
Bibliography ........ 34

## Principal aspects of expression in music

***Melody*** is the main, nearly universal, expressive aspect of pitch, formed by the changes of the fundamental frequency (FF) between the consecutive musical tones that share the same registral position and timbral characteristics. These tones become integrated into a coherent melodic line, if the intervallic size of frequency changes stays within the Temporal Coherence Boundary (Noorden, 1975) and abides by the voicing rule of following a leap by steps in the opposite direction (Huron, 2001). Otherwise, we hear a few melodies as a result of segregation of the audio stream (Cambouropoulos, 2008). Melody in pitch-oriented music cultures is usually projected as a "horizontal" axis that reflects music's unveiling in time. The main reason for this must be the consecutive "linear" combinatorial organization of melody (see ***Motif***, below), of which creators and perceivers of melody are aware. Those music cultures where combinatorial melodic organization is weak (e.g., in timbre-oriented traditions of Nenets or Nganasans) do not project music as a horizontal axis (see Nikolsky, 2016d).

***Harmony*** is another important expressive aspect of pitch, formed by the consecutive changes in the number and the numerical order of harmonics between the harmonic series of the concurrently sounding tones. In essence, harmony represents the ongoing comparison of the periodic part of the harmonic spectrum, taken as a series of “slices” on strong metric times (usually, beats). The number of harmonics in the harmonic series of each of the concurrently sounding tones that coincide—i.e., share the same frequency values or small-integer ratios (½ and ⅔)—determine the extent of fusion of these tones or the lack of it (Benson, 2007). Maximal fusion occurs when the FFs are 1200 cents apart, and minimal fusion—at 100 cents or below. Changes between different levels of fusion are perceived as harmonic progressions, where low fusion is characterized by the experience of harmonic tension, whereas high fusion—by relaxation (Parncutt, 1989). Harmony is usually projected as a “vertical” axis of pitch due to its power to integrate the concurrent sounds. This association is nearly universal amongst music cultures that have developed multi-part traditions.

***Texture*** is a specific scheme of hierarchic arrangement (melodic “horizontal” as well as harmonic “vertical”) of all tones within a musical work in a way that is different from purely melodic, harmonic, and rhythmic organization. Elements and components of texture are determined by the number of parts, their functional relations (e.g., accompaniment or counterpoint), and the presence of conventional structural components (e.g., so-called Alberti bass) in harmonic/melodic arrangement (Huron, 1989). Music genres in polyphonic and homophonic music tend to cultivate characteristic textures through their application (Nazaikinsky, 2013). Thus, the genre of march is characterized by the texture consisting of the energetic melody in the highest register and the accompaniment by massive homogenous chords. In contrast, the genre of barcarole is characterized by the lazy ongoing wave-like melodic figuration in the accompaniment to the song-like melody. Each texture breaks into a number of “stream segments” at its surface level of perception (Cambouropoulos, 2010), forming discrete components—”textural cells,” used as bricks in constructing a texture by vertical and horizontal grouping of various complexity, functionality, and hierarchic relations (Nazaikinsky, 1972). Such “textural cells” are ascribed specific semantic values by means of public conventions (Kholopova, 2002). For instance, strictly chordal accompaniment is associated with the expression of determination, obsession, or strength—in contrast to the delicacy and sensuality of the Alberti bass. The typology of textures varies along three axes: density (a.k.a. number of parts and/or voices), frequency range (Benward & Saker, 2009), and functions of parts (Berry, 1987).

***Rhythm*** is the main expressive aspect of the time domain, formed by the relative duration of each of the consecutive tones in a textural layer—quantized according to a certain division ratio (2, 3, etc.). This quantization (rounding to the closest normative rhythmic value) occurs involuntarily in both production and perception of rhythm (Honing, 2002). Rhythm is central to sequential grouping of tones. Shorter tones become grouped with the longer tones, so that the latter terminate a group (Jones, 2016). Rhythmic changes reflect how short or long the tones are in relation to each other, intuitively correlated with the help of a meter (see below).

***Meter*** is another important expressive aspect of time in music, often confused with rhythm due to the influence of the prosodic theories of versification in poetry (Kharlap, 1978). In music, meter is the number of unstressed beats grouped together with a stressed beat before the occurrence of the next stressed beat, thereby generating metric grouping. The latter is distinguished from rhythmic grouping by its regular and uniform sound. All beats are metrically equal, forming a continuous pulse that is retained within a music work or its sizable section, generating the music movement of a certain character—unlike rhythmic values that as a rule form divisions and therefore differ, generating diversity and contrasts. Technically speaking, a metric group acts like a container that is filled up by a variety of rhythms. Meter and rhythm oppose each other not only as “form” versus “content,” but also by their perceptory input. Metric pulse is automatically generated according to the perceptual and statistical prevalence of rhythms

identified in a musical movement (Large & Snyder, 2009). Beats become grouped based on the perceived periodicity of stresses generated by longer and louder tones as well as changes in melodic direction and harmony (Jones, 2016). An important distinction between meter and rhythm is that the former is generated involuntarily in the brain even at the absence of tones (filling up the pauses), while the latter is triggered by actual physical stimuli—both are processed by different parts of the brain (Geiser et al., 2009).

***Tempo*** is yet another important temporal aspect of music, deceptively "obvious," yet often misunderstood due to its dependency on meter and rhythm: fast rhythms in slow tempo can be confused with the metric pulse in fast tempo. Technically, tempo is the average pace of beat within a sizable span of musical movement. Tempo is always averaged because even in the music movement that appears to be steady in pace, tempo as a rule keeps fluctuating within ±10% of its mean value. Climaxes (see below) usually trigger noticeable acceleration of tempo, whereas cadences—decelerations, which are perceived as normal (i.e., their absence is usually seen as a lack of human feel, mechanicity, or immature performance) (Fabian, 2014). In addition to the actual speed, tempo is determined by a specific character of musical movement, such as rushing, comfortable, or lagging (London, 2004). The importance of this characterization is that every music composition is conceived in a specific tempo that becomes "absolute"—i.e., the optimal pace for a given music piece (Levitin, 1994). For each music culture, there is an assortment of most common tempi—e.g., for Western classical music it is a 12-tempi system (Nazaikinsky 1972), where a tempo, optimal for a given music piece, is defined as a range of bmp (beat per minute) values within which the "feel" for that tempo remains the same (Garbuzov, 1950). The ability to infer the optimal tempo for a specific configuration of other aspects of expression is considered one of the highest merits in performance skills in Western classical music.

***Articulation*** in music is the manner and the extent of attaching or detaching successive tones within the same part of the texture or within a voice of a part (Keller, 1973). Technically speaking, articulation slightly modifies an exact rhythmic value of a tone while keeping its metric value intact: e.g., the articulation of *staccato* shortens the nominal rhythmic value (akin to the sound production of mandolin or xylophone), whereas *legato* sustains it to the full nominal value, making sure that both successive tones are indeed connected (Jerkert, 2003). Some instruments, such as piano or guitar, can perform *legatissimo* by making the end of one tone overlap the onset of the following tone—thereby generating a momentary harmonic dyad in a monophonic line. Performance practice puts in place diverse styles of rendition of tones in melody: e.g., staccatissimo, marcato, mezzo staccato, non legato, portato, tenuto, legatissimo (each indicated by notation signs or/and verbal indications) for Western classical music (Keller, 1973). Articulating music in these ways generates grouping (e.g., a "legato-staccato" group)—in addition to rhythmic, melodic, and textural grouping. The same articulation grouping can run through an entire musical composition (e.g., Chopin's Etude op.25 No.9 in E Major features an articulation figure "legato-legato-staccato-staccato"). Once established for the expressive performance of melody, articulation becomes applicable to the accompaniment: its melodic figurations, double-notes (see below) and chords. The typology of articulation styles and generated groups varies along 3 axes: connectedness, discreteness and compactness (Braudo, 1961).

***Phrasing*** in music complements articulation in joining or breaking successions of tones, but does it at a higher hierarchical level—in relation to successive groups of tones (Keller, 1973). Phrasing specializes in subdivision of a melodic line in a way similar to how the flow of words in language is segmented in logically coherent sentences (Chew, 2001). The principal difference from language is that musical phrases are determined by the alternation of harmonic, melodic, and rhythmic tension with relaxation, thereby conjoining both in a single syntactic unit. The ultimate relaxation that completely overrides tension marks a "cadence" (see below) at the end of the phrasal unit. The cadence has to be emphasized by the injection of a pause in excess of the available metric time (a.k.a. "***caesura***") in order to break the metric regularity of a musical movement (Tiulin, 1969). In Western music, phrasing is sometimes marked in a music score

by slurs, but more often it is left at the discretion of the performer and used to evaluate their performance skills.

***Music form*** is a complex integrative aspect of expression in music, like texture, closely related to the latter as well as to the phrasing. Typically, changes from one type of texture to another, combined with the end of a phrase, mark the end of one section in a music form and the beginning of another section (similar to paragraph breaks in written speech) (Tiulin, 1969). However, music form reflects one more dimension of music that has no analogs in speech—music form is built by changes of ***musical themes***. The latter can be defined as a musical idea that is fixed in musical structures, characterized by considerable completeness, salience, and originality—allowing one to recognize these structures upon hearing them again—and often used continuously or reused within the same musical work (Mazel, 1979). The notion of "musical theme" is often incorrectly described as peculiar to the Western classical music. In reality, many non-Western music traditions recognize thematic organization. For instance, in Carnatic tradition, a musical composition is often segmented in 3 sections (*Pallavi*, *Anupallavi* and *Caranam*) based on the treatment of a musical theme (Agrawal, 2018). Although thematic analysis evolved within Western classical music, it should be applied to any form of music (Val'kova, 1992). The concept of *thema* (Gr.'proposition') emerged in rhetoric theory, and therefore suits any application where the listener needs to remember a particular expression essential for a musical work (Drabkin, 2001e). Thematic repetition, variation, contrast, or recapitulation (i.e., the return of a theme after some other material), recognizable by some salient feature(s) (Réti, 1951), usually of melody and harmony, but sometimes of rhythm or texture—constitute a universal trait of music (Val'kova, 1992). In cases, where "theme" lacks clear structural completion (e.g., in many preludes) or cannot be reduced to melody (e.g., chord progressions of jazz "themes"), we can talk of "thematic material"—i.e., the complex of musical structures employed towards making a given musical work—disregarding their completeness and salience (Mazel, 1979). Then, music form can be defined as changes of the thematic material within a music work, where the simplest case is an exact reproduction of the same material (AA), and the most complex is the so-called unveiling form based on the ongoing contrast (ABCDE…).

***Dynamics*** is perhaps the most obvious aspect of expression in music, formed by changes of amplitude between consecutive or/and concurrent tones within the musical texture. A specific tone or textural element can be marked by a relative increase or decrease in intensity. What is often overlooked is that gradations of dynamics (e.g., fortissimo, mezzo forte, forte, mezzo piano, piano, pianissimo for Western classical music) (Berndt & Hähnel, 2010) constitute structurally stable conventions adopted within a musical culture (Thiemel, 2001)—although highly variable in reproductions of the same music by different performers, but quite stable per person (Garbuzov, 1955). Music practice generates dynamic distinctions that generally correspond to the extent of affective intensity of the music (Dean et al., 2011).

***Register*** is one of the expressive aspects of timbre that is the most important for tonal organization of music, whether it is timbre- (e.g., Jaw Harp) or pitch-oriented. Although the scientific research of musical register has remained highly controversial since its introduction by Manuel Garcia in 1830, recent technological advances led to the establishment of some consensus (Henrich, 2006). Generally, vocal register is defined as perceptually distinct regions of vocal quality that can be maintained over some ranges of pitch and loudness (Titze, 2000). In distinction from linguistic register, musical register is a phonation frequency range in which all tones are perceived as being produced in a similar way and which possess a similar voice timbre (Sundberg, 1987). Instrumental registers follow the vocal music paradigm. Each musical instrument, capable of producing a range of pitches comparable to a human voice, possesses a number of registers defined by noticeable changes in tonal quality between the timbrally homogenous groups of tones (Drabkin, 2001d). Not to be confused with the parameter of pitch (i.e., the dimension of higher-lower), registers differ not only in relative height but in timbral coloration as well (e.g., darker-lighter, thicker-thinner, etc.). For instance, the entire range of clarinet breaks into 4 registers: somber *chalumeau* (E4-E5), dull *transition* (E5-B5), bright *clarino* (B5-C7), and piercing *altissimo* (C7-A7) (

Miller, 2014). Three-four registers are typical for most types of singing voices (Titze, 1988) and musical instruments (Patterson et al., 2010). However, different musical cultures adopt different attitudes to registers—some smoothen registral contrasts while others increase these contrasts (Yemelyanov, 2000). Registral organization is crucial for the genesis of musical modes (see below): as a rule, a musical mode is made of tones that belong to the same register.

***Ambitus*** is the entire range of sounds producible by a human voice or a musical instrument or/and the entire range of sounds engaged in a musical composition or its part (instrumental or vocal). Technically speaking, ambitus is a sum of all registers in a particular sound source, encompassing all the pitches available for production of music.

***Instrumentation*** is the principal expressive aspect of timbre in the practice of music composition. Instrumentation can be defined as selection of a type of musical instrument and vocals (contrary to the etymology of the word "instrumentation") that are most suitable for a specific expression, as well as the art of combining these instruments or/and vocals together, and distributing the components of musical texture between them (Kreitner et al., 2001). Although today, "instrumentation" and "orchestration" are used indiscriminately, traditionally, orchestration was associated with scoring for orchestra, whereas instrumentation—with scoring for instruments solo and for small-scale ensembles. The discipline of instrumentation emerged first, accompanying the rise of ensemble music during the late Middle Ages, and orchestration followed as large ensembles became more common in the performance practice of the 17th century. Instrumentation and orchestration rules were coined after the standardization of string orchestra, wind band, choir, and finally, "double" and "triple" orchestra (Spitzer & Zaslaw, 2004). The main challenge of instrumentation is that such a *multidimensional* aspect of expression as timbre (McAdams & Giordano, 2016) makes it very difficult to predict and control the outcome of mixing various instruments and vocals, further complicated by the interaction of registers, dynamics, articulation, harmony, and melodic motion. Timbres of individual instruments and vocals can blend into a new timbre (e.g., the mix of clarinet and oboe), remain discrete yet complement each other (violin and flute), or repel each other (harp and horn), depending on such factors as similarity and synchrony of spectral centroids and attacks (Sandell, 1995) or salience of individual harmonics (Nazaikinsky & Rags, 1964). The simplest case of instrumentation is sustaining a single timbre throughout the entire piece of music (solo). The most complex is the combined use of orchestra and choir that features multiple foreground and background layers, changing over time, with contrasts between tutti, soli, as well as between orchestral and choral groups (Banshchikov, 1997). Each type of musical instrument and vocals constitutes a specific tone color in a palette of the arranger (Meyer, 2009). Certain combinations of instruments (string trio, wind quintet, orchestra) and vocals (duet, quartet, choir) form stable settings, used to create music of certain semantic content (e.g., march or waltz).

The distinction between register and instrumentation is that register is bound to pitch and underlies instrumentation (every instrument and vocal breaks into registers). Yet another distinction is that register plays a formative role in music based on timbre or indefinite pitch (e.g., ekmelic). There, the building elements are defined in regards to their position within a register as "degrees" of a musical mode rather than "pitch-classes." The aspect of instrumentation completely misses this formative modal function. Combinations of timbral colors of different instruments do not form specific musical modes. On the other hand, instrumental timbres often blend, forming new composite colors (for example, clarinet + oboe). There is nothing remotely similar in the domain of register—registers don't blend. It can be generalized that register fundamentally opposes instrumentation: register is based on timbral similarity, whereas instrumentation—on timbral contrasts.

# Elementary building blocks of music

***Pitch-class*** (a.k.a. "degree" in a scale) is a specific pitch value that is systematically used within the same musical composition for making melodies and harmonies. The term "class" reflects the presence of some kind of mental model of a pitch level to be used over again and again. In practice, performers do deviate slightly from such a model occasionally throughout a music work due to the expressive requirements of specific melodic or harmonic contexts. These deviations do not exceed a semitone (100 cents), usually remaining within a quarter-tone. Deviations about the size of a semitone are usually recognized as *chromatic alterations* (see below). The presence of frequent fluctuations in pitch greater than a semitone indicate that the music under question is based on indiscrete pitch—so-called *ekmelic* (see below). Musicologists order pitch-classes that are used within a music work in a musical ***scale***—a ladder-like succession of ascending (or descending) pitch values. Once represented in this way, each pitch-class receives a number that reflects its numerical position in a scale from the bottom up—which is called a "***degree***." The latter is less informative than "pitch-class" since it reflects the membership in a scale, whereas "pitch-class"—the membership in a musical mode, the notion of which contains much more information than that of "scale" (see below).

***Timbre-class*** is an equivalent of a pitch-class in composition of the timbre-oriented music (e.g., Jaw Harp music). Timbre-class can be defined as a specific timbral coloration with unclear or inconsistent pitch value (it does not sustain throughout a music work), which is used together with some other timbre-classes. For instance, in Jaw Harp music, one timbre-class can be articulated on the vowel "O", whereas the other ones, on "E," "I," and "A." As a rule, timbre-classes that are used together in a single music work share some common timbral characteristics—in other words, they match each other in some timbral respect (e.g., for Jaw Harp, this can be a buzzing sound quality).

***Interval-class*** is an elementary unit of harmonic organization in pitch-oriented music, formed by the synchronous engagement of 2 discrete pitch values. In music cultures that do not use multi-part textures, interval-classes are often formed between the consecutive melodic tones. Such melodic interval-classes differ in their typology and semantic qualia from harmonic interval-classes (Nikolsky, 2015a). Consistent usage of pitch-classes usually generates interval-classes. By the same token, a set of pitch-classes generates a set of interval-classes—every pair of the constituent pitch-classes obtains a corresponding interval-class, the sum of which defines the intervallic structure of this set (e.g., the C Major set contains 7 pitch-classes: C-D-E-F-G-A-B and 54 interval-classes: 7 unisons and octaves, 5 major 2nds and minor 7ths, 2 minor 2nds and major 7ths, 3 major 3rds and minor 6ths, 4 minor 3rds and major 6ths, and 6 4ths and 5ths). However, interval-classes are autonomous from pitch-classes, because different pitch-classes in the same set can form the same interval-class (e.g., in the set of C Major, 5 different pairs of pitch-classes: C-D, D-E, F-G, G-A, and A-B—all share the same interval-class of the major second).

***Rhythm-class*** is a specific rhythmic value adopted as a reference for duration of pitch- or timbre-classes within a music work. Rhythmic values can fluctuate in their actual duration in the so-called expressive timing (Todd, 1985) that generally tends to emphasize phrasing and exaggerate rhythmic contrasts by prolonging anchored tones while shortening tones in passages, ornaments, or short tones in rhythmic groups that contain contrasting rhythmic values (Repp, 1990). The extent of expressive timing depends on the choice of tempo (Repp, 1995). Listeners expect to hear music expressively timed (Repp, 1998) and become accustomed to this by mere exposure to a given style of music (Honing & Ladinig, 2009). Somehow, expressive timing actually helps categorization of rhythm-classes.

***Tone-class*** is a general notion of an elementary structural unit of music that incorporates pitch-, timbre-, interval-, and rhythm-classes across all types of music. This notion is useful to describe a model of musical sound that is adopted as a standard by a specific music culture.

# Means of integration of musical elements

***Musical mode*** is a set of musical tone-classes selected by music-users according to some mutual concordance of these tone-classes in relation to one or more aspects of music expression (e.g., timbre, pitch, articulation). This is the most general definition of a musical mode, designed to cover all types of music, including instrumental music that does not use instruments capable of producing multiple pitches and melodies (e.g., African talking drums) as well as vocal music that does not use discrete pitch changes (e.g., solitary rapping to one's own finger snaps). In essence, any musical mode by definition involves some kind of *harmonization* of sounds—i.e., a limited set of pitch-, timbre-, interval-, or rhythm-classes are chosen for their mutual compatibility in some respect so that they would generate harmonious progressions of sounds. In this sense, a melodic pitch-set can employ harmonious relations despite the general opposition of melody to harmony (see above).

For music based on discrete pitches, musical mode can be defined as a set of pitch-classes that match each other in their intervallic relations for the desired expression and thereby support generation of melodies for this expression. Usually, the constituent pitch-classes of a mode form systematic relations, where one (or more) classes subordinate the other classes, and at least some pitch-classes abide by the combination rules that regulate construction of melody (Powers et al., 2001). The generative power of musical mode is especially pronounced in folk music, where each mode usually can be recognized by its specific modal intonations and motifs. This prompted Beliayev to come up with the most laconic definition for all varieties of modes in pitch-oriented music: "musical mode is the generalization of certain types of melodic motion in relation to the intervallic structure of these types" (Beliayev, 1990).

Pitch-oriented "musical mode" should be distinguished from "scale": the latter simplistically reduces a musical mode to pitch values alone, disregarding the melodic and harmonic rules for generation of music as well as the hierarchical relations between the member pitch-classes. A great caution should be taken not to mistake mode for scale: e.g., the very same scale—G-A-B-C-D-E—can represent a hexatonic G-major mode or an incomplete A-minor key, where the low "G" would act as the VII degree to the tonic "A" (Hornbostel, 1913). Zemtsovsky calls these superficially congruent structures "homonymous" and warns of the need to analyze the underlying music and take in consideration its modal syntax (Zemtsovsky, 1974).

***Ekmelic mode*** is a peculiar type of tonal organization, characterized by the exclusively melodic *monophonic* (see below) arrangement of the *indefinite-in-pitch* tones according to their registral position and melodic functions, and employing the *variable-in-size* (stretchable) interval-classes to make melodies (Nikolsky, 2015a). The term "ekmelic" (Gr. 'unsuitable for melody') originates from the ancient Greek music theory (treatises by Cleonides, Gaudentius, and Ptolemy), where it referred to disharmonious and unclear sounds—in opposition to the term "*emmelic*" (Gr. 'suitable for melody') that was employed by Aristoxenus and his school in reference to sounds with distinct intervallic structure (Lippman, 1964). Ekmelic intervallic typology was theorized by Kholopov as a peculiar type of gliding intonation and "dirty tones" deliberately and consistently used in certain styles and genres of music (Kholopov, 1988). Even entire music cultures of northeastern Eurasia (e.g., Nenets, Nganasan—see (Nikolsky, 2016g)) are known to use exclusively ekmelic tonal organization (Alekseyev, 1986).

Ekmelic modes, as a rule, employ no more than 4 degrees—defined by their melodic functionality, frequency of use, consistency in tuning, relative duration, dynamic stress, and position within a register (Alekseyev, 1976). Technically speaking, "degrees" of ekmelic modes do not constitute "pitch-classes" since they are defined not by any specific pitch value but by their order within a register and melodic function. It seems that all forms of musicalized speech (e.g., "*parlando*" recitative, poetic declamation, recitation of religious canonic texts, or solo rapping) abide by the principles of ekmelic tonal organization (Nikolsky, 2015a).

***Khasmatonal mode*** is yet another type of tonal organization that uses registers rather than pitch values to define its "degrees." The term "khasmatonal" (Gr. *khasma*—'hollow') was introduced by Wiora to refer to

such melodies in which one tone broke registrally apart from the rest of the tones—separated with a systemically unclosed gap at its every occurrence (Wiora, 1959). This term has been adopted for the musicological analysis of those indigenous folk melodies that combine indefinite pitch with salient leaps (500 cents or larger), accompanied with pronounced timbral modulations and/or expressive use of several contrasting registers (Alekseyev, 1986).

Leaps and timbral contrasts tell khasmatonal organization from ekmelic: the former is recognized by abrupt transitions from one register to another, combined with melodic leap (Alekseyev, 1986)—whereas the latter is based on melodic consonance: ekmelic melodies stay within the same register and most of the time engage steps (Nikolsky, 2015a). It can be said that ekmelic mode is "consonant" (see below) by design versus khasmatonal mode that features a "dissonant" component. In both cases, "degrees" of a mode are established by their registral position and the successive order of a tone in question in a melodic contour of the consistently repeated melodic formula (many, if not most, ekmelic and khasmatonal melodies are strictly formulaic).

***Timbral mode*** is a musical mode that integrates a number of timbre-classes based on their concordance in one or more aspects of timbre (Nikolsky & Benítez-Burraco, 2022). Timbre is a complex multi-modal aspect of expression, usually experienced in synaesthetic terms: visual, haptic, and kinesthetic (Zacharakis et al., 2014). Timbre modulates along at least 4 axes: hue, texture, brightness, and sharpness (Ivanchenko, 2001). Attempts to establish common dimensions of timbre by means of the semantic differential technique (Osgood et al., 1957) established 4 orthogonal factors: full-empty, dull-sharp, colorful-colorless and compact-diffused (von Bismarck, 1974). The verbal attribution here should not be dismissed as inaccurate: it was experimentally found adequate in revealing correspondences between the numerical rating of dissimilarities of the sounds and the verbal descriptions of their similarities and differences (Sa.k.a.ta et al., 1996).

The same perceptual mechanisms that were identified in relation to the perception of harmonic organization in tonality (Lerdahl & Jackendoff, 1985) apply to perception of timbre, since roughness makes an elementary timbral attribute that is based on the sensation of rapid fluctuations in the amplitude envelope, quite similar to the oscillations of a frequency wave (McAdams & Giordano, 2016). Like succession of pitches, timbral changes can generate the impression of fluctuation between tension and relaxation (Clarke, 2001; Nazaikinsky & Rags, 1964; Paraskeva & McAdams, 1997; Volodin, 1972) and might even present hierarchical tonal organization—which is the common foundation for all musical modes (Bolger & Griffith, 2005; Lerdahl, 1987; Volodin, 1970).

At present, timbral modes are cultivated in the instrumental music for such instruments as Jaw Harp, musical bow, and bullroarers—widespread over the world—as well as in such local traditions as *sukute* (struck and blown tubes) of Solomon Islands. In vocal music, timbral modes serve as the principal means of tonal organization of rasping songs of Chukchi, Eskimos, Koryaks, Itelmens, Evens, Yukaghirs, Nganasans, and northeastern Yakuts (Sheikin, 2002), as well as Inuits and Ainu (Nattiez, 1983), and rasping-whistling songs of Altaians, Kha.k.a.ss, Tuvans, and Shor people (Sheikin, 2002). Obviously, rasping cannot be reduced to pitch contours and, in its essence, it manifests a deliberate "anti-pitch" orientation. Hence, the task of reproducing a specific rasping style must by definition exclude the pitch aspect. In addition to rasping and whistling, the non-pitch timbral performance devices include clicking with the tongue, wheezing, sizzling, whispering, and yelling (Sheikin, 2002). "Stylized" talking (deliberate mannerism in recitative) should be added here, such as in Yakut *chabyrgakh*—"tongue-twister"—which uses a selection of phonemes to build "melodized" prosodic formulas, repeated in accelerated fashion with a comic effect (Alekseyev, 1976).

Linguistic phonemes, conjoined into syllables, are often treated as "timbre-classes" in music traditions of Siberian and Far Eastern indigenous people. Thus, Nivkhi use a specific assortment of meaningless syllables (a.k.a. vocables) to produce *za.k.a.nga* and *koka chnyr* (Jaw harps) and *tiatia chkharsh* (drum-log) music (Mamcheva, 2012). Many indigenous Jaw Harp traditions are distinguished by the repretories of syllables used in a manner of "tone-classes" to construct music—often these repertories differ from typical syllables of the native languages of Jaw Harp performers (Zagretdinov,

1997). Indigenous musicians report that when conceiving Jaw Harp music, they think in terms of what they call "articulation scales" (Nikolsky et al., 2017). Rasping traditions also employ specific vocables as units for construction of a song, the assortment of which distinguishes one tradition from another (e.g., Chukchi from Koryak).

***Rhythmic mode*** is a type of tonal organization peculiar to rhythm-based varieties of music, such as *Kumi-daiko* ("drum-circle") in Japan or African "talking drum" traditions. A glimpse into this organization is provided by a well-documented theory of modal rhythm in Western tradition, where the value of each duration was determined by its position within a rhythmic group (a.k.a. 'modus'), comprised of a patterned succession of long and short values (Roesner, 2001). By no means, modal rhythm is unique to Western music: quite similar modal implementation is documented in treatises on Arabic (Touma, 1996), Persian (Farhat, 2004) and Indian (Clayton, 2000) classical traditions—to such extent of similarity that one tradition could be confused with another (Ferreira, 2015).

Rhythmic proportions are most commonly estimated in terms of binary or ternary divisions that produce a set of standard durations—usually 3-5 divisions. One or two of them serve as a primary rhythmic unit to build the music movement, thereby forming a "metric grid" that is employed to round up the actual duration of a tone to the closest valid rhythmic value (Large & Kolen, 1994). This is what seems to underlay the phenomenon of modal rhythm. Just as pitch-classes are allowed to follow or not follow one another, or require an alteration for ascending or descending motion, rhythm-classes are restricted to certain ratios and ordering patterns that both are alterable in a certain way: e.g., a dotted "long-short" rhythm can be "over-dotted" in a suitable context (Fabian & Schubert, 2008).

***Double-note*** (a.k.a. "harmonic interval," "vertical dyad," "bichord," or "double-stop") is the simplest case of integrating 2 discrete pitch values into a single harmonic percept—provided, both occur synchronously, are separated by the interval no greater than octave, and belong to the same register (i.e., there are no timbral contrasts between both tones). Ear training specialists know perhaps better than any researcher that each of such double-notes is recognized by the unique "color" of its interval-class. Thus, a 5th sounds empty, cold and stable, whereas a 4th—empty, cold, but unstable, whereas a 3rd—stable but filled and somewhat warm—such characteristics come very handy in teaching young children, so that teachers usually present each new interval-class as a creature, whose look fits the qualia of the sound of that interval (Borovik, 2006). In the methodological literature on ear training in Russia, this peculiar attribute of harmonic intervals is called "fonizm intervala"—which can be translated as "intervallic sonority," and implies a particular coloration of the synchronous combination of tones, determined by the interaction of their partials (Nazaikinsky, 1977). There is some experimental evidence that trained and untrained listeners discriminate intervals by their qualia, and their judgements are influenced by register (Costa et al., 2000). Each harmonic interval seems to be attributed some emotional meaning (Oelmann & Laeng, 2009).

***Chord*** (from Lat. *accordo*—'agree') is a simultaneous combination of 3 or more different pitch-classes, separated by the intervals no greater than an octave and timbrally similar. The notion of "chord" reflects the integrative power of harmonic fusion, when acoustically consonant (see below) intervals make the entire complex of concurrently sounding tones appear as a single auditory unit (Parncutt, 1989). This fusion is responsible for the integration effect, so that a chord obtains its own harmonic qualia, making it qualitatively more than a mere sum of qualia of the constituent harmonic intervals (Cook, 2009).

In Western music, the discovery of chords occurred throughout the 14-16th centuries, at first triads (chords consisting of two 3rds), and thereafter other structures (Rîpă, 2010). At first, chords were theoreticized as a collection of intervals—until Johann Walther defined "chord" as a single structural unit in 1732 (Kon, 1973). The signature of such understanding is the emergence of "inversion of chords"—i.e., the practice of transposing just one of the chordal tones an octave higher or lower and considering the resultant structure functionally and semantically the same as the original (ibid.). Once chords were established as discrete entities in harmonic organization, music practice has forged a diverse typology of

chords: triads, 7th-, 9th-, and 11th-chords, as well as 4th-chords (Kholopov, 1988). The latter are exceedingly common in hypermodal and pentatonic music (see below). The use of chords by no means is limited to the Western classical tradition. Quite a number of indigenous African traditions have developed homophonic (see below) organization and adopted chords as structural units—although Kubik qualifies them as "timbre-harmonic clusters" (Kubik, 1999).

***Consonance*** is a psychoacoustic experience of good match between a few musical tone-classes, experienced as pleasure and relaxation. Consonance is usually opposed to dissonance (see below). Most importantly, the dialectic relation between consonance and dissonance serves to generate anticipation that the dissonant combination of tones will be replaced by the consonant combination—thereby supporting the grouping of dissonant and consonant sounds in a single syntactic unit. Hence, the perceptual opposition of consonance and dissonance is paramount for melodic and harmonic integration of musical sounds.

***Harmonic consonance***. For Western classical music, since the time of Pythagoras, consonance has been defined as a harmonic "natural law" that smaller ratios (1/2, 2/3, 3/4) between the FFs of the concurrently sounding tones necessarily produce concordant sonance (Benson, 2007). The perceptual substrate of this harmonic concordance is the psycho-acoustic phenomenon of fusion of tones (Stumpf, 1911). However, the correspondence between small integer ratios and the experience of consonance is not that straight-forward, and depends on register, dynamics, and timbre amongst other things. Thus, later research established that under certain conditions smaller-ratio combinations of tones can produce dissonant impression (e.g., an interval of a 3rd in a very low register, as played by a double-bass, sounds rough and unpleasant).

In practice, consonances are identified *negatively*—as the absence of dissonance (see below), where the dissonance is associated with the difficulty of grasping a dissonant relation versus the ease of detecting a consonant relation (Terhardt, 1974). The experience of consonance is rooted in the anatomical properties of the auditory nervous system and depends on periodic oscillation of harmonically consonant sounds (Tramo et al., 2001). In essence, nervous pathways in the brain resonate to acoustic consonance, which greatly facilitates its perception. For this reason, infants, brain-damaged individuals, and even non-human animals have been found to discriminate acoustic consonance from dissonance.

One caveat here is that not all music cultures adhere to the Pythagorean correspondence of smaller ratios to euphony. Quite a number of indigenous folk traditions reverse the correspondence and hold rough sonorities for euphony (Brandl, 2008)—sometimes even featuring parallel melodic motion in major and minor 2nds, considering them pleasant like the sound of bells (Messner, 1989). Despite this confusion between acoustic and aesthetic evaluations of consonance (Cazden, 1958), the distinction between acoustic harmonic consonance and dissonance seems to be universal. It is just that different cultural traditions assign different aesthetic values to the universal experiences (Vassilakis, 2005) or do not assign them at all, if consonant/dissonant relations are deemed unimportant for a given music culture (McDermott et al., 2016).

***Melodic consonance***. The experience of consonance is not limited to harmony. Register, loudness, timbre, melodic contour, and reverberation are known to make euphonic impressions and therefore can be adopted as "consonant" by a given musical culture (Cazden, 1980). One type of consonance that, like harmonic consonance (yet autonomous from it), seems to constitute a biologically rooted universality is melodic consonance. Yury Tiulin was first to note a paradox that a harmonic minor 2nd is a harsh dissonance, while a melodic minor 2nd is pleasant to the ear (Tiulin, 1937). This explains the aesthetic constraints posed by quite a number of music traditions, such as plainchant that cross-culturally forbids melodic leaps and restricts a melodic line to stepwise motion only.

The euphonic impression originates from the same source as harmonic consonance—the processing ease—but owes to a different perceptual mechanism. Melodic leap retains the "trace" of a previous pitch level in the listener's memory (Larson, 1997) due to the segmentation effect of trespassing

the Temporal Coherence Boundary (Noorden, 1975). In contrast, melodic step "erases" such a "trace," thereby releasing short memory of the need to cache previous tones (Larson, 1997). Komar termed this peculiar effect "melodic displacement" of the resolving tone by the resolved tone (Komar, 1971)—in accordance (unknowingly) with Tiulin, who qualified this as a melodic resolution that supports a harmonic resolution in multi-part music at the phrasal ends in cadences (see below). It was experimentally confirmed that this melodic displacement works best for resetting the "pitch integration window" (Plack & Watkinson, 2010).

The euphonizing effect of both harmonic and melodic consonances must be responsible for their common binding in cadences. Both of them have been shown to be detectable by newborns (Masata.k.a., 2006; Stefanics et al., 2009) and might occur automatically or near-automatically (Sussman, 2005; Sussman et al., 2007). "Melodic step" also finds a nearly universal synaesthetic equivalent in "locomotive step" that is definitely easier than leap and therefore associated with comfort and pleasure.

***Dissonance*** is a psychoacoustic experience of mismatch between a few musical tone-classes, related to the experience of displeasure and tension, which inherently generates a desire for some continuation, in hope for the negative experience to be changed by some positive one. Hence, dissonance tends to propel the music movement towards a coming consonance. The event of a consonance replacing a dissonance is called a "resolution" and can occur in harmonic as well as melodic domains (Nikolsky, 2015a).

***Harmonic dissonance***. For Western classical music, dissonance has been traditionally defined as large-ratio proportions between the FFs (Benson, 2007). The perceptual substrate of experiencing a harmonic dissonance is the psycho-acoustic phenomenon of roughness of tones (Helmholtz, 1877). Audition of dissonance evokes aperiodic firing of neurons that are engaged in representation of the partials of each of the concurrent tones, further complicated by the interference between those partials that are too close to each other (Tramo et al. 2001). For this reason, processing dissonance is difficult and less desirable than consonance.

However, priming of acoustic dissonance to the aesthetically pleasing experience can cause habituation to the negative aspects of pitch ambiguity in those music cultures, where musical instruments that generate rich aperiodic spectra, such as gongs and xylophones, occupy an important place and serve as referents for tuning in the practice of ensemble playing (Schneider, 2013). This could explain the origin of the deliberately rough, so-called diaphonic (from Gr. *diaphōnos*—'discordant'—as opposed to *symphōnia*—i.e., 'concordant') music (Brandl, 2008). Similarly, an acoustic consonance can be primed to aesthetic dissonance: e.g., in Western polyphony prior to the 15th century, the interval of a 3rd was considered dissonant and required resolution (Knighton & Fallows, 1997).

***Melodic dissonance***. The Temporal Coherence Boundary (Noorden, 1975) determines which melodic intervals are experienced as consonance and which as dissonance—as a function of tempo: the slower the tempo, the smaller the threshold (300 cents presenting the lowest limit). Melodic leaps, as a rule, require an effort to detect whether they occur within the same audio stream or mark the segmentation and the onset of a new stream (Bregman, 1994). This task makes all leaps by definition "complex"—associating them with melodic unease and tension (Rags, 1980). If melodic steps generally tend to bind melodic motion, leaps always obstruct its fluidity, and the largest leaps have the power to disturb and disrupt the integrity of a melody (Tiulin, 1937). In this sense, leaps constitute melodic dissonance. The wider the leap, the more disruptive it is—since the leaped tone requires a longer duration (thereby halting melodic motion) and the subsequent reversal in the direction of a melodic contour (or its freezing on about the same pitch level), which obstructs the melody (Huron, 2001).

The requirement of avoiding two successive leaps in the same direction and the rule of following a leap by steps in the opposite direction or repetitions of the same pitch act as a form of melodic resolution of tension. The leap-size dependency in the extent of melodic dissonance synesthetically matches the locomotive experience. Greater leaps require greater preparation, greater momentum, and extra time for "landing"—both for melodic motion and for physical locomotion (Larson, 2012).

Consistent leaps without melodic resolution break the melodic stream in two in a phenomenon known as "implied polyphony" in musicology (Bukofzer, 2008) and "segregation of audio stream" in psychoacoustics (Bregman & McAdams, 1979). Thus, most Baroque compositions for an unaccompanied flute solo employ such implied polyphony. The ongoing zigzagging motion, especially in fast tempo, imposes a constant strain on the listener, calling for the prolonged melodic dissonance to be systemically "resolved" by dividing a single monophonic dissonant melody into two imaginary consonant streams (where the peaks of the ongoing leaps are interpreted as one melodic line, whereas their troughs, as another line). The phenomenon of implied polyphony is by no means limited to Western classical music (Burnett, 1980).

***Cadence*** is the conclusion to a musical phrase (see below) that usually involves a particular harmonic and/or melodic formula and serves as the primary means of syntactic organization in music—often presenting different gradations of "finality" (Rockstro et al., 2001). Harmonically, cadence is characterized by the high harmonic fusion that comes to replace low fusion or roughness, which is combined with the increase in "tonal gravity" (see below). Such a change is experienced as tonal *stability* that follows *instability*. Melodically, cadence is often (more so in pre-Romantic Western and folk music) marked by the descending steps terminating leap(s) or replacing the ascending steps (the very word "cadence" originates from Lat. *cadere*—'fall') in order to reach the "finalis." The latter, in modal theory, refers to the most stable tone in a musical mode. In a key of a music culture that cultivates the tonality, such a final tone is called "tonic." Rhythmically, melodic cadence usually brings longer rhythmic values to replace shorter values (Mazel, 1952). Thematically, cadential progressions contrast the preceding thematic material by engaging fewer motifs and pitch classes in a formulaic arrangement (Quinn & Mavromatis, 2011). All these four aspects of cadence combined support the experience of tonal *resolution*—the replacement of tension by relaxation. The latter generates the "anchoring" effect by means of grouping the unstable dissonant tones with the subsequent stable consonant tones (Bharucha, 1984).

***Climax*** in phrasing serves as the opposite of cadence (from Gr. *klimax*—'ladder'). It marks the point of the highest tension, usually achieved by the ascending melodic motion that reaches the most unstable pitch-class, shortening of rhythmic values, engaging the overall *crescendo* (i.e., gradual increase in intensity), and increasing harmonic dissonance (Braudo, 1961). Shaping a musical phrase in a dynamic wave, with its crest on the climax point, has a power to emphasize the integrity of this phrase, which is paramount for music that features complex textures or long phrases. In performance practice, one of the greatest merits in the mastery of phrasing is the ability to define and emphasize a single climax point for each phrase.

***Tonal gravity*** is a theoretical framework, constructed by musicologists to account for the fluctuations in tension, which are experienced as momentary increases and decreases in stability or instability. Tonal gravity was elaborated to the fullest in the "energetics" theory by the distinguished German music theorist Ernst Kurth (1931, 1991; Rothfarb, 1988). In his model of "musical forces," Steve Larson updated Kurth's approach, bringing it in accordance with the more recent psychoacoustic findings (Larson, 2012). Larson's model draws the parallel between mechanical laws that govern the motion of a body, on the one hand, and tonal rules that govern melodic motion from tone to tone, on the other hand. This parallel has been known among musicologists and composers for at least a few centuries and included such notions as inertia, momentum, gravity, mass, and force (independent conceptualization of these factors was presented in the theory of musical movement by Tiulin, developed in the 1930s). The alternative gravitational psychoacoustic models were presented by Bharucha (1996) and Lerdahl (Lerdahl & Krumhansl, 2007).

These gravitational models reflect a peculiar experience of unstable tones acting as a driving force that raises "expectancy-tension" in the listener, elevating attention for the subsequent events in anticipation of the coming of stable tones (Margulis, 2005). As a result, unstable tones are expected to

resolve into the closest-in-pitch stable tone—which Larson calls "magnetism," distinguishing it from "gravity" of stable tones that "attract" unstable tones. This model seems to be more than a mere metaphor—there is some experimental support for the propensity of instability to charge melodic motion (Hubbard & Ruppel, 2013; Larson & McAdams, 2004; Larson & Vanhandel, 2005; Vega, 2003). At the heart of the experience of musical stability seems to be the combined effect of longer rhythmic values, higher frequency of occurrence, greater stability in tuning, overall higher intensity, and placement on stronger metric time and at the phrasal ends (Nikolsky, 2017).

***Tonality*** is a method of tonal organization, where all pitch-classes in a pitch-set are subordinated to the tonic and the tonic triad (see below) and are categorized through their functional relations to one another (Lerdahl, 2009). Such organization evolved during the 16-17th centuries in Western Europe through standardization of intervallic relations between parts in a polyphonic texture (see below), tonicization of phrasal beginnings/endings, and formation of typical harmonic progressions based on vertical harmony (Wienpahl, 1972). Ability to hear equivalent concords between multiple parts and recognize them as a single typological percept was the greatest achievement of Renaissance musicians, enabling the culture of *thinking in chords* (Nutting 1974).

Inter-relations between the successive chords, in turn, forge the uniform melodic relations between their constituent pitch-classes by setting the hierarchy of "tendency tones"—i.e., standard successions of specific pitch-classes in a major or minor key (Huron, 2006). Tonal functions are manifested in the peculiar experience of imaginary hearing of the "implied chords" in a strictly monophonic melody (Holleran, Jones & Butler 1995). Listeners and makers of Western music (classical and popular) routinely experience implied chords, which enables harmonization of familiar tunes by ear even by musically untrained individuals.

Progressions of chords are regulated by the rules of tonal tension and resolution, implemented through 3 principal harmonic functions: *tonic* (stable), *dominant* (unstable) and *subdominant* (neutral). They were conceptualized by Riemann in 1893, based on the harmonic theory formulated by Rameau in 1726 to reflect on the common practices of the late Baroque music (Hyer, 2012). Such functions have acquired formative power in generating a music form, different types and different sections of which are characterized by different distribution of harmonic functions (Berry, 1987; Bobrovsky, 1978; Caplin, 1998).

Structurally, tonality can be recognized by the *importance of chords* for tonal organization, *permanence of tonic function,* abundance of *alterations* (see below) and frequent pronounced changes in *stability/instability* (Kholopov, 1975). Importantly, tonality is not limited to Western classical music. Thus, a parallel development of the so-called Mediterranean tonality (Manuel, 1989) can be inferred in the music practice of Arabo-Andalusian, Persian, Gypsy, and Jewish cultures throughout the 17-19th centuries (Nikolsky, 2016e). Unlike the Western tonality, based on major-minor keys, Mediterranean tonality relies on peculiar hemiolic (see below) and so-called dominant keys (ibid.).

Tonality, as a rule, is implemented through **keys**. Although tonality and key are often understood as synonyms, the important distinction is that tonality constitutes a general method of tonal organization that includes a finite number of keys, whereas key is a particular instance of application of tonality, characterized by its reproduction of the same tonal scheme from a given pitch. Thus, one key can differ from another key while representing the same tonal structure (e.g., C Major versus D Major). Hence, tonality can be understood as the general idea of "keyness" (Hyer, 2008). Yet another important distinction is that specific keys can deviate from the standards imposed by tonality (see "dominant keys" in the discussion of "key" below).

***Tonicity*** is the general term that covers all manifestations of tonal "gravity" (see above) in generation of perceptually "stable" tones in melody and harmony. In psychoacoustic literature, a closely related concept is "anchoring" (Bharucha, 1984, 1996, 2002). The notion of tonicity is founded on the concept of "tonic," coined in 1710 by Saint Lambert (Lester, 1989). Tonic is the most "stable" tone in a musical key that is used as *finalis* to terminate a music work (Powers, 2001).

A functional extension of "tonic" is the concept of ***tonicization***—i.e., the establishment of a temporary new "tonic" in a musical key on a degree other than the initial tonic—in essence, a brief modulation (see below) that quickly returns to the initial tonic (Drabkin, 2001f). Although this term was introduced by Schenker for the classical music of the Common Era Period, it is useful for the analysis of other kinds of music, especially those that use pitch-based modes, where fluctuations in stability are more common than in tonal keys.

A closely related phenomenon is "double-tonic complex" (Bailey, 1986). In Russian musicology it is called "modal mutability" (Bakulina, 2014), introduced by Yavorsky (Yavorskii, 1908). Modal mutability is the ongoing transfer of the tonic function from one pitch-class to another within the same mode, in the manner of a seesaw, most commonly involving the I-VI or I-II degrees (Nikolsky, 2015a). Such systemic "mutation" in gravity is exceedingly common in folk music. Tonicization and mutability, as well as weaker gravity in the structurally simplest musical modes, e.g., oligotonal (see below), prompted Alekseyev to distinguish between 3 forms of tonicity (Alekseyev, 1976, 1986):

1. The weakest of them is "*registral anchoring*," carried by the "leaning tone" (*opornyi ton*) that is more stable in tuning than the other tones in the ekmelic, khasmatonal or simplest oligotonal modes.
2. "*Motivic anchoring*," carried by the "stable tone" (*ustoi*), subordinates tones in a motif to a tone that features greater rhythmic value, intensity, tuning stability, and is placed on strong metric time. This organization characterizes more complex oligotonal and mesotonal (see below) modes.
3. "*Modal anchoring*," carried by the "tonic" (tonika), extends subordination to the entire mode and, possibly, through the entire music work or its entire section. Modal anchoring can implement double-tonic.
4. To these 3 Alekseyv's forms, I would add the 4th form of anchoring—"*tonal anchoring*," carried by the tonic triad in a key (tonality as opposed to modality), where the tonic receives the power to terminate a music work and/or its sections.

***Tonic triad*** is a chord that engages the I, III, and V degrees of a key (or a mode) to define its axis of tonal stability. Although the I degree here remains gravitationally superior to the III and V degrees, which is manifested in its position at the root of a tonic chord, the III and V degrees also carry the tonic function (i.e., have power to terminate a phrase). The use of *tonic* triad is not exclusive to Western tonality. Before the concept of harmonic tonic function within a tonal *key* was theorized in 1722 (Rameau, 1971), Johannes Lipius declared the tonic triad to form the basis for musical *modes* in 1612 (Berger, 2006). Amongst the non-Western cultures, Georgian multi-part singing uses a sophisticated typology of chords within the modes that all exhibit a pronounced tonicity (Arom, 2010).

***Key*** is a form of tonal organization based on a single tonic and gravitational hierarchy, which determines harmonic progressions, melodic tendencies, the arrangement of cadences, and music form within an entire music work (Hyer, 2001b). The concept of key evolved within the Western classical music (Berger, 2006), under the influence of theories of temperament, generally called to facilitate the use of ensembles of musical instruments to support vocalists (Barbour, 2004). Singers prefer to sing a familiar song in their most comfortable register, disregarding which exact pitch-class then is adopted as a tonic and how euphonious the key based on such a tonic actually sounds. However, for the supporting instruments, especially keyboards, some keys sounded noticeably worse (sometimes unacceptably) than others (Barbieri, 2003). The idea of defining an ideal uniform tuning and configuration of pitch-classes, reproducible from any pitch, was famously advocated in Bach's Well-Tempered Clavier (but had numerous predecessors).

However, the idea of subordinating a musical composition to a single principal pitch-class in a hierarchic framework greatly predates the Baroque era and spans beyond Western classical music. The earliest documented implementation of musical key was in the ancient Greek *tonoi*—brought to life by

the need to retune a musical instrument (lyre and harp, prevailing during the Classic period) in order to play a certain *harmonia*. The convenience of playing one preexisting tune in one mode immediately after another tune in a different mode, without stopping and retuning an instrument, incentivized a performer to keep the same tuning (i.e., key) for as long as possible and make the necessary adjustments all at once during the modulation from one mode to another (Nikolsky & Benítez-Burraco, 2022). Since the number of alterations (see below) had to be minimal to make modulation as fluent as possible, performers had to carefully select a string from which to start playing in a new mode and, if necessary, to transpose a preexisting tune to match the tuning of the strings. This practice subordinated modes to keys: modes became defined in reference to a specific key. This relation is still observable in Western classical theory—a minor key supports 3 modes: natural, harmonic, and melodic, where the transition from one mode to another does *not* constitute a modulation. The integrity of a key secures the common scheme of tonal organization that underlies all of these three modes.

The need to unify modes in some easy uniform way is not limited to the Western tradition. Similar traditions of tuning that follow a rigid procedure of deriving one pitch value from another, so that the entire set of pitch-classes is inferred from some initial "central" pitch-class, exist in many advanced music cultures that feature rich musical instrumentarium (Beliayev, 1990). All such tuning traditions can be qualified as *keys* in contradistinction from *modes*. Keys are not found only in those cultures where vocal music prevails over instrumental, transmission remains exclusively oral, formal music theory is absent, and performers do not conceive music in terms of producing intervals of a certain value (Kvitka, 1973).

Keys can feature exceptions to some important principles of tonality. Thus, so-called ***dominant keys***, common for Spanish classical music and traditions of the Near East and Middle Asia, feature gravitationally weak I degree while tonicizing the IV degree. As a result, the entire key sounds as if its I degree constitutes a so-called dominant harmony (one of 3 principal harmonic functions of tonality: tonic, dominant, subdominant) in relation to the IV degree (Sposobin, 1969). The prevalence of dominant keys characterizes the flamenco tradition of Spain.

***Modality*** is a method of tonal organization of music which is alternative to tonality and historically preceded the latter. The concept of modality was introduced in 1810 by Alexandre Choron (Choron & Fayolle, 1971)—in opposition to conventional "keys" of Western classical music as the generalization of tonal organization of the "modes" of the ancient Greek music, seen as the paradigm of modal composition. François-Joseph Fétis placed Choron's modality into the evolutionary framework, in 1840 (Fétis, 1994). He viewed modality and tonality as not mutually exclusive—reserving for modality a place in contemporary music practice of some genres and styles. Indeed, both often coexist in the same music culture: thus, Western classical music and folk music traditions can use either of these two (Powers et al., 2001). Currently, the term "modality" implies divergence from the rules of Western common practice period in favor of a more ancient or non-European descent (Tagg, 2003).

Modality can be defined as a principle of tonal organization where all tones in a musical mode are united by melodic relations—i.e., by their melodic functionality: capacity to initiate, finalize, or mark the climax in melodic phrases. Unlike tonality with its clear-cut universal rules and rather abstract semantics, modality accounts for melodic direction and contour (different rules for ascending and descending—so-called bidirectionality—or for stepwise and skipwise motions) and associates a particular tonal structure with a specific ethos and/or genre. Structurally, modality can be recognized by the absence or *unimportance of chords* for tonal organization, *permanence of scale*—i.e., scarcity of alterations (see below)—and *weak tonicity*, where a phrase can end on any tone, not necessarily "tonic" (Kholopov, 1975). In folk multi-part music, modality generally favors structurally irregular sonances over uniformly structured chords.

# Complexity of tonal organization

***Alteration*** in music refers to the raising or lowering of a pitch-class in a mode or a key by a semitone. Such events trigger a momentary increase in tension due to the expectation to discover the reason for this deviation from the norm and the resulting anticipation of the return to that norm (Margulis, 2005). Quite unprecedented wide adoption of alterations in the ancient Greek performance practice around the 5th century BC corresponds with the increase of public interest in music theater, music competitions, and the rise of programme music—posing the need for increased emotionality (Nikolsky, 2016b). The same factors have been associated with the use of alterations in Western classical music (Brothers, 1997). The most common causes for alteration are either modulation to a different mode (see below), tonicization (see above), melodic ornamentation (e.g., "shakes," a.k.a. mordents), or the need to increase the gravity of a tone that immediately follows the alteration (in which case it is usually a proximal degree of a key or its mode).

The first documented use of alteration comes from ancient Greece: e.g., the score of "Hymn to the Muse" by Mesomedes (modulation from Lydian to chromatic Hypolydian mode), 2nd century AD (Hagel, 2009). Alteration is inherently related to the presence of formal prescriptive music theory, professional music occupation, and some kind of notation—all necessary to fix the standard pitch values. For this reason, the consensus of ethnomusicologists leans towards denying the presence of alterations in folk music.

Although folk melodies might give an impression of alterations, in reality, most of the time they constitute "expressive intoning." Thus, the software-based frequency analysis of transcribed archive field recordings of Lithuanian traditional songs, followed by the statistical analysis of frequency values, revealed that many of the notated alterations were not justified and presented a cultural bias, introduced by notators (Ambrazevičius & Wiśniewska, 2008). Norman Cazden proposed to call such instances of expressive intoning "modal inflections" (Cazden, 1971). The principal difference is that alteration constitutes a pitch-subclass or even a special pitch-class (as in late Romantic music), whereas "modal inflection" retains the same pitch-class (Nikolsky, 2016a). Rare alterations in folk music usually originate from inclusion of melodic intonations from contrasting genres, which leads to semantic clash that increases emotionality of a mode—usually making this mode bidirectional (like the melodic minor in classical music) (Zemtsovsky, 1972).

***Modulation*** is the instance of transition from one musical mode or key to another. Although modulation was theorized within the framework of Western tonal music, and its ancestor music systems, such as Medieval Western, Byzantine, and ancient Greek, similar devices have been implemented in other advanced music systems, such as Indian, Arabic, and Chinese. The principal difference from alteration that frequently accompanies modulation is that modulation alters the *entire* pitch-set rather than a single pitch-class—which makes a much stronger semantic impression. For this reason, musicians as well as non-musicians familiar with common modes and keys detect modulations by ear in Western (Korsakova-Kreyn & Dowling, 2014) and non-Western traditions (Raman & Dowling, 2012). As a rule, modulation from key to key is semantically less significant (unless the change is between minor and major keys) than modulation from mode to mode. Modes are usually associated with certain ethos or similar qualia (Shestakov, 1975), so the modulation from mode to mode involves the change of the corresponding ethos, throwing listeners into a new emotional state.

Modulation in indigenous (village-like) folk music seems to be rarer than in music cultures that have formal music theory and professional education, because weak gravity enables easy tonicization of any degree, whether accidental or systematic (as in modal mutability—see above). When a village musician wants to express a different emotional state, he/she usually starts a new piece of music instead of modulating. There are generally 3 types of modulation that for some reason received taxonomic recognition only in Russian music theory, although they are applicable to all types of pitch-oriented music and are exceedingly common in practice (Kholopov, 1988):

1. “modulation” per se, characterized by the use of a common harmony (usually, a chord that is present in both source and target keys) to smoothen the transition;
2. “deviation” that does not engage common harmony, instead relying on alteration (usually, a short-living temporary tonicization that is not supported by the cadence in a new key); and
3. “invasion” that uses neither common harmony nor alteration, but without any preparation “jumps” right in a new key (or mode).

***Oligotony*** is the simplest form of emmelic (i.e., based on definite pitch) organization, characterized by the use of no more than 1-3 pitch-classes, 100-400 cents apart within a narrow ambitus that is not wider than about half-octave (Sheikin, 2002). The term “oligotonal” (Gr., *oligo*—'few') was introduced in 1927 by Kvitka (Kvitka, 1971) to refer to musical modes that featured fewer than 7 pitch-classes (directed against those theorists who considered such modes “incomplete” diatony). Oligotonal modes often contain one or two poorly defined pitch-classes, whose melodic functionality abides by the ekmelic principles, suggestive of oligotony’s descent directly from ekmelic models (Nikolsky, 2015a). Oligotonal modes usually have at least one well-defined pitch-class that is stable in tuning and “consonant” in a sense of its capacity to smoothly resolve and terminate melodic phrases. Oligotonal pitch-classes, as a rule, feature *heterarchical* rather than hierarchical relations—i.e., they are *coordinated* rather than *subordinated* to each other and do not support accumulation of tension by pitch (only by rhythmic, dynamic, and timbral means). Oligotonal modes can be monotonic, ditonic, or tritonic.

***Monotonic modes*** constitute the simplest form of oligotony, common for religious and magic applications. They use only 1 melodic intonation (see below)—that of repetition of the same pitch. Monotony is quintessential for the transition from ekmelic to emmelic organizations (Nikolsky & Benítez-Burraco, 2022). Monotony defines a pitch-oriented musical mode in the simplest possible way—by continuously reproducing the same pitch level and therefore directing attention to its pitch value. In this way, monotony “harmonizes” multiple occurrences of the same pitch-class.

***Ditonic modes*** represent the advance of monotony towards greater expressive capacity and introduce the simplest melodic syntax. It is based on the distinction between a “leaning” tone and a “supporting” (a.k.a. auxiliary) tone, distinguished by the consistency and accuracy in hitting the same pitch level. The auxiliary tone is usually less stable in its tuning, rhythmically shorter, placed on metrically weak time, and is less frequently used than the “leaning” anchor (Nikolsky, 2020). Ditony is common in epic tales, religious recitatives, and children play-songs.

***Tritonic modes*** introduce a “contrasting tone” that challenges the “leaning tone” by posing an alternative anchor. This anchor usually comes very close to the primary leaning tone in its rhythmic durations, dynamic intensity, metric placement, and frequency of use—only missing the stability in tuning: it typically drifts further apart from the leaning tone as a song progresses and the performer becomes more excited (Nikolsky, 2020). Another common tritonic scheme is the combination of a single leaning and 2 auxiliary tones above and below it. The lower one usually marks phrasal ends. Tritony can be found across many different genres.

***Mesotony*** presents the next level of complexity after oligotony. The term “mesotonal” (Gr., *meso*—'middle') was introduced in 1973 by Starostina to distinguish those modes that used 5-6 pitch-classes from oligotonal modes based on their differences in tonal organization (Starostina, 1973). Notably, mesotonal modes engage hierarchical relations and therefore support more complex syntax. After a few decades of fieldwork, Soviet ethnomusicologists adjusted Starostina’s low threshold to include tetratonic modes (which Starostina qualified as oligotonal). This correction came about since oligotonal modes are rarely encountered in Russian folk music (Starostina’s specialty), while being common in indigenous music of Northeastern ethnicities (Sheikin, 2002). The subsequent analyses of the collected Siberian folk songs

revealed that in reality oligotonal organization featured a narrow range of about 400 cents, and therefore did not support more than 3 pitch-classes. Hence, mesotony had to be redefined as the collection of 4-6 pitch-classes, distributed within the ambitus no larger than an octave, and therefore often incorporating a gap (consistently used leaps up to about 500 cents that remained unfilled for the entirety of a music work). Mesotonal classes are usually quite well-defined in pitch and support hierarchical relations, where an auxiliary degree can obtain its own supporting tone. Mesotonal modes can be tetratonic, pentatonic, or hexatonic.

***Tetratonic modes*** are much more widespread than tritonic and can be found across the globe in many different genres (Nikolsky & Benítez-Burraco, 2022). What separates tetratonic modes from tritonic modes is that 4 pitch-classes generate 24 possible combinations of tones (a.k.a. intonations—see below), whereas 3 pitch-classes support no more than 6. For this reason, tetratony supports advanced syntactic organization, whereas tritony does not. This is because the important cognitive threshold of chunking specifies 9 chunks (7 +/-2) as an upper limit for working memory to process information (G. A. Miller, 1994). "Chunk" here is a set of items treated collectively as a single unit. For melody, this means that a melodic phrase that engages more than 9 intonations (melodic dyads) requires further chunking of chunks—i.e., it requires grouping of intonations into motifs and adoption of such motifs as structural units in their own right. In other words, it demands hierarchical relations and complex syntax. Chunks of a "maximally compressed" code pose an even lower limit of 4 +/- chunks (Mathy & Feldman, 2012).

Longer tetratonic melodies are likely to exceed this limit, especially if to take into consideration that rhythmic grouping adds extra "chunks" to pitch "chunks," which is crucial for monodic performance settings (see below). Such melodies can feature 2 auxiliary relations, paired into one (e.g., III-IV degrees subordinated to I-II). The most probable candidate for cultivating such organization was the rhapsodic tradition of ancient Greece (and likely earlier Vedic tradition), where singing of epics was based on 4 pitch-classes, defined by the tuning of a 4-string lyre that supported singing (West, 1981).

***Pentatonic modes*** introduce even greater complexity by affording 120 melodic dyads. This makes a pentatonic music system by definition hierarchical and syntax-based. However, here it is important to distinguish between the diatonic and non-diatonic pentatony (see below). Majority of music cultures known as "pentatonic" today (Day-O'Connell, 2007) abide by the *diatonic* principle and constitute *anhemitonic* pentatony—i.e., they do not use semitones in the interval set of a musical mode or a key. This is in contrast to *hemitonic* pentatony, most famous in Japanese classical music, that uses semitones (Malm, 2000) and to *equidistant* pentatony, found in Africa (Kubik, 2010).

Yet another important distinction is that mesotonal pentatony does not feature ***octave equivalence***—what acousticians call "pitch chroma" (Hutchinson & Knopoff, 1978)—i.e., the notion that tones an octave apart represent the same pitch-class (e.g., C1 and C2 constitute the "same" pitch-class). The modal functionality of octave as a registral boundary should not be confused with the acoustic property of the interval of an octave to double a pitch value (Werner, 1948). Since the working range of mesotony is smaller than octave, it does not support accurate reproduction of all pitch-classes over an octave range—perhaps, one pitch-class, but not all. Full octave equivalence requires the discovery of the diatonic principle of tonal organization (that requires the presence of math-based music theory).

Absence of octave equivalence means that if the melody exceeds the ambitus of an octave, the pitch value of a pitch-class that crosses an octave boundary does not constitute an integer of the pitch value of a lower pitch-class (e.g., in what appears to be C1-D1-F1-G1-Bb1-C1-D2, the upper "D2" might be unequal to the double value of the lower "D1"). Most of pentatonic modes in indigenous folk cultures, e.g., in North America or Africa, are not fully octave-equivalent and are register-dependent (Arom et al., 2007).

***Hexatonic modes*** raise the bar of complexity even higher—affording 720 dyads—and are more likely than pentatonic modes to feature octave equivalence. However, unlike all smaller-set modes that form distinct schemes of tonal organization (monotony, ditony, tritony, tetratony, and pentatony), each characterized with their specific features, hexatonic modes seem to constitute a modification of either pentatony or heptatony (see below). Music works sustained in a hexatonic mode can present a heptatonic mode without one pitch-class, where the melody operates according to the rules of a tonal key. Alternatively, a pentatonic mode can be enriched with an extra pitch-class in such situations, where a verse of a song is pentatonic while its chorus is hexatonic. Norman Cazden regarded the hexatonic modes of Anglo-American folk tradition as a hybridization of the pentatonic folk Irish, Scottish, Amerindian, and West African traditions, on the one hand, and the heptatonic tradition of the Western classical music, on the other hand (Cazden, 1971). Similar view was expressed by Alekseyev in regard to Eurasian music. He considered pentatony and heptatony competing methods of conceiving music, the former oriented towards keeping tonal tension to the minimum, in contrast to the latter (Alekseyev, 1986). Alekseyev observed the consistent use of hexatonic modes in the Volga area, geographically enclosed by the heptatonic West and the pentatonic East. The most typical hexatonic structure, semitone-tone-tone-tone-third-tone (B-C-D-E-G-A), indeed combines the "leading tone" at the bottom, which is used like the VII degree in a heptatonic mode (e.g., C-D-E-F-G-A-B), with the tensionless pentatonic-like melodic motion at the top of a mode. Rare cases of genuine hexatony occur as a result of cultivating a melodic dubbing by minor thirds or major sixths in 2-part singing: e.g., "Istrian scale" in Croatia and Romania (A-Bb-C-Db-Eb-Fb) (Kirigin, 2014).

***Diatony*** is a method of defining the intervallic typology for musical modes and keys, based on deriving 7 pitch-classes by means of building the circle of 5ths (e.g., F-C-G-D-A-E-B), thereby producing the "diatonic scale" (C-D-E-F-G-A-B) that consists of a group of 2 whole tones (C-D-E), separated from a group of 3 whole tones (F-G-A-B) by a semitone (Kappraff, 2002). This method was already known in ancient Babylon (Crickmore, 2014), was imported by ancient Greeks (Franklin, 2002), and further spread to Western Europe and Western and Central Asia—which has been interpreted by many scholars as a proof of its universality (Johnson, 2008). However, cultural origins of diatony are indicated by experimental studies on acquisition of music skills: e.g., children younger than 3 years were shown to lack the ability to recognize diatonic organization (Trehub et al., 1986).

The problem is that many folk modes that look diatonic (i.e., formally comply with the circle of 5ths) are known to have been produced by other operations (Beliayev, 1990). To add to confusion, diatonic modes can acquire non-diatonic modifications of interval-classes through cultivating a few preferred modal intonations and transposing them from one degree of a mode to another or via importing a modal segment (dichord, trichord, or tetrachord) from some other mode for some peculiar expression—thereby generating "***mixodiatonic***" (quasi-diatonic) modes (Kholopov, 1988).

Yet another point of controversy is whether pentatonic and hexatonic modes can be diatonic. Modern Western theorists limit diatony to heptatonic modes and keys, stressing its opposition to chromatic alterations (Drabkin, 2001a). Indeed, the term "diatonic" originates from "*diatonikos*" (Gr. 'stretched' or 'spread-out') in reference to the rule that the largest interval in a tetrachord could not exceed the sum of two other intervals. In ancient Greek theory, this is what distinguished the *diatonic* genus from *chromatic* and *enharmonic,* which both divided a tetrachord in a more uneven way. Hence, the etymology of "*dia*" ('through') + "*tonos*" (tone) implied a smooth, gapless distribution of tones.

However, Chinese music theory did employ the circle of 5ths to generate pentatonic organization, just restricting the use of 2 extra pitch-classes to avoid semitonal tension that was deemed aesthetically undesirable for music (Daniélou, 1995). Other music cultures of antiquity shared similar "diatonic" approaches to pentatony (Gauldin, 1983). Limiting the circle of 5ths to just 4 rather than 6 members has been implemented in quite in a number of *folk* music systems—following the rules of informal, orally transmitted music theory that was developed to support the practice of playing fret-based string

instruments (frets make intervals visible) (Beliayev, 1990). For this reason, Catoire in 1925 suggested to qualify such forms of pentatony "***proto-diatonic***" (Catoire, 2015).

Whether a pentatonic mode is diatonic or not seems to be determined by the octave equivalence (Maceda, 1990). Accurate reproduction of all pitch-classes over the octave range requires uniformity in their tuning. Octave equivalence secures this uniformity. In fact, without octave transposition (which requires octave equivalence), building the circle of 5ths is not possible for practical reasons: the progression of 6 fifths in one direction takes the span of 3.5 octaves, which is beyond the ambitus of the simplest musical instruments used as a reference for tuning. Therefore, the *ambitus of melodies and instruments* that are common in a music culture determines whether pentatony is diatonic or not. Music cultures that do not use melodies wider than an octave simply cannot be diatonic (the occasional presence of 1-2 octave-equivalent pitch-classes can be a by-product of the acoustic production, such as falsetto, rather than modal octave equivalence).

***Chromaticism*** in classical music is usually defined by modern Western theorists as opposite to diatonic—which generally follows the historic opposition of *musica ficta* to *musica recta* during the Middle Ages—and is based on division of an octave in 12 equal semitones to use semitonal increments for chromatic alteration (Dyson & Drabkin, 2001). Russian theorists emphasize the notion of "*chroma*" (Gr. 'color') in "recoloring" of the normative diatonic pitch-classes for greater expressiveness and consider chromaticism as a 6-grade continuum between the purely heptatonic and purely dodecaphonic organization (Kholopov, 1988). Here is the entire list:

1. "*inter-systemic* alterations"—enabling modulations from one diatonic key to another,
2. "*intra-systemic* alterations"—supporting temporary deviations within the same key,
3. "*leading-tone* alterations"—modeling the semitonal VII-I relation on other diatonic degrees (e.g., F#-G) to increase tonal tension,
4. "*passing* chromatization"—using successive chromatic alterations in a scale-like fashion (e.g., C-C#-D), especially in melodic dubs and chordal progressions,
5. "*mixodiatonic* chromatization"—mixing the harmonic and melodic patterns of parallel keys (i.e., C Major and C Minor: for example, E-F-A-G-Eb-D-C) within the same phrase,
6. "*autonomous* chromaticism"—abandoning the diatonic framework in favor of dodecaphonic modes, either full 12-tone or incomplete.

Chromaticism in antiquity had a different etiology (see "enharmonic genus" below). It appears to be peculiar to ancient Greece (Gurney, 1994), being invented there in the 5th century BC (first described by Aristoxenus) and associated with the "avant-garde" music, introduced by professional citharodes (Hagel, 2009). The rise of new music probably answered the call for modal creativity in extremely popular music competitions (Christesen & Kyle, 2013) that financially rewarded originality, innovation, and technical proficiency (Csapo & Wilson, 2009). As a result, for about 500 years, chromatic music greatly exceeded the popularity of diatonic music that obtained the stigma of being too predictable and unimpressive (Franklin, 2002). Despite the vocal opposition by Plato and Aristotle, who viewed chromatic music as detrimental to the Greek society due to its (in their words) effeminate and sensual character (Stamou, 2002), chromatic "sweetening" of intervals by illuminating a subtle delicacy in their tonal shading was highly appreciated for their capacity to express a range of feelings, from "pleasant" to "lugubrious" (Franklin, 2005). Greek chromatic music was a tonal system designed to present emotional theater as a form of entertainment. For this very reason it was rejected as sinful by the Fathers of the Church (Shestakov, 1975). And the same theatricality that had attracted the ancient Greeks inspired the flourishing of chromatic "mannerism" towards the end of Renaissance, driven by the efforts of such theorists as Vicentino to restore the ancient Greek genera (Maniates, 1993).

However, the Greek chromaticism differed from the Western classical chromaticism in one extremely important respect—it was implemented on a system level as 13 chromatic keys, built by

stitching together tetrachords from each of the 12 semitones between *Hypodorian F2* and *Hyperphrygian F3* (Hagel, 2009). Later theorists of antiquity updated 13 keys of Aristoxenus to 15 (Mathiesen, 2001). Western chromaticism lacked such systemic sophistication and grew anew, independently from Greek sources, at first as the alteration of the diatonic hexachordal system (Bent, 1984), and after the 15th century, as the "leading tones" towards the triadic tones (Clough, 1957).

***Hemiolic mode and key*** is a peculiar type of tonal organization that stands between diatonic and chromatic, forming octave-equivalent "*mixodiatonic*" or "*mixochromatic*" structures by mixing diatonic and chromatic tetrachords, while featuring a characteristic "hemiolic gap" (from Gr. *hemiolia*—the 1½:1 ratio) in one or both tetrachords (Kholopov 1988). This organization is common for Arabic, Turkish, Persian, Andalusian, Gypsy, Jewish, Balkan and Middle Asian traditional music—constituting a landmark of the "Mediterranean tonality" (Nikolsky, 2016e). The origin of hemiolic structures seems to be the ***enharmonic genus*** of ancient Greek music: thus, modern Syriac chant uses modes that are nearly identical to enharmonic structures described by ancient Greek theorists (Nikolsky, 2016c).

Enharmonic genus emerged simultaneously with diatonic genus, both credited to Olympus, in the 7th century BC (Barker, 2007). Probably, both genera constituted 2 alternative methods of turning the "Archaic trichord" (E-F-A) into a tetrachord (West, 1981). In contrast to the gapless diatonic tetrachord (E-F-G-A), its enharmonic version featured a prominent gap between the higher *hegemon* (E-F𝄳-G𝄫-A) and 3 lower tones that together comprised *pyknos* (Gr. 'pinch'). The distribution of tonal gravity was determined by the opposition between the stable fixed marginal tones of a tetrachord (*phthongoi hestotes*) and the unstable movable (i.e., alterable) tones in its middle (*phthongoi kinoumenoi*). The latter were systemically altered, whenever this tetrachord was turned into enharmonic or chromatic.

This organization featured 2 levels of hierarchy: the stable couple of marginal tetrachordal tones (E-A) subordinated the unstable couple inside the tetrachord (F-G), but if the tones of the former were coordinated, the tones of the latter were not—the lower of them (*parhypate*—i.e., F) subordinated the upper tone (*lichanos*—G) (Kholopov, 2006). This gravitational scheme, as well as the naming scheme of Greek pitch-classes, the exclusively descending direction of alterations (West, 1992), plus the statement of pseudo-Aristotle that descending melodies were perceived more harmonious than ascending melodies because of greater euphony of lower tones (Aristotle & Mayhew, 2011)—all of these suggest the association of ascending motion with accumulation of tension, while descending motion—with relaxation.

Such tetrachordal modal organization characterizes the music systems of the entire Mediterranean region and Central Asia (Nikolsky, 2016e). The Greek enharmonic and chromatic genera (the latter presenting a later semitonal simplification of enharmonic microtonal shading of the unstable degrees) probably were adopted by the Sabaean civilization together with the Dionysian cult (enharmonic genus was associated with the Dionysian genre of dithyramb) and passed on to the Qaynah culture and the earliest Hijaz school of the Arabian music (the Hijaz mode of the maqamat exemplifies the typical hemiolic structure). Parallel cultural transmission might have occurred through the Jewish and Gypsy agency. At any rate, hemiolic organization constitutes its own special kind, different from Western keys with chromatic alterations as well as pentatonic keys with their diatonic gaps.

***Multitony*** presents a further expansion of a mode to include 7-11 pitch-classes, featuring a pronounced hierarchy of stability and instability and distinguished by the contrast of even and odd degrees in a stepwise scale. Multitonal modes often feature modal mutability (see above) of 2 tonics (usually, one initiating a phrase and another terminating it). The concept of "multitonal mode" was introduced by Starostina as a product of diatonization—in contrast to mesotonal modes (Starostina, 1973). What distinguishes multitonal modes from keys is limited octave equivalence and presence of bidirectional melodic rules (different intervals reserved for ascending versus descending motion). Multitonal music often spans over the ambitus of an octave and forms the so-called false relations between the low and high versions of the same unstable degree: most commonly, sharpened low VII ("leading tone" in reference to

the tonic I degree) versus natural upper VII (e.g., G#-A-B-C-D-E-F-G) or normal low VI versus flattened upper VI degree (e.g., A-B-C-D-E-F-G-Ab).

The prevalence of multitony in a music culture tends to promote the formation of multi-part tradition (Jordania, 2006). Mesotony usually expands into multitony by means of "***triadic induction***": a pitch-class, added above/below a stable pitch-class, acquires a supporting function, whereas a pitch-class added above/below an auxiliary pitch-class becomes stable (Mazel, 1952). In a hemitonic scale, this invariably produces *triadic functionality*: three odd degrees (i.e. I-III-V) share a stable function, while three even degrees (II-IV-VI) jointly carry a supporting function. This arrangement promotes melodic dubbing by a second part, generating streaks of parallel thirds (C/E-D/F-E/G …). Kubik describes such multi-part dubbing in African indigenous music, calling it a "counter-note pattern" (Kubik, 2010). Once discovered, such melodic dubbings easily upgrade to form parallel triadic motion by adding one more part a third above the upper duetic part. The systemic assignment of stability to the odd degrees and instability to the even degrees generates hierarchical relations and subordination of pitch-classes. Multitonal modes are usually heptatonic, featuring 1 or 2 false relations, but sometimes can form octatonic modes.

***Heptatonic modes*** constitute the most common implementation of multitony. One reason for this is that "triadic induction" (see above) keeps extending the number of pitch-classes to no more than 7. This is because the stable tonic triad, made of the odd degrees, sets the axis of gravity—surrounded by the unstable triads on the II and low VII, both of which harmonically *contrast* the tonic intervallic structure (Nikolsky, 2015a). If the tonic triad is major (C-E-G), the II triad is minor (D-F-A), and the VII triad is diminished (B-D-F). If the tonic triad is minor (A-C-E), the II triad is diminished (B-D-F), and the VII triad is major (G-B-F). This contrast promotes the emergence of harmonic functions and facilitates implicit learning of modal (or tonal) melodic and harmonic rules.

The tonic axis is limited to the tonic third (I/III) and triad (I/III/V). Further expansion of tonicity to the 7th-chord (I/III/V/VII) is impossible, since the principal melodic function of the VII degree is that of a "leading tone"—the most unstable pitch-class that is eager to resolve into the tonic by the ascending step.

Yet another, perhaps even more important reason for the current prevalence of heptatonic modes in the world is that the simplest ratios of 5/4, 4/3, 3/2, 5/3 and 1/2 produce exactly I, III, IV, V, VI and VIII=I degrees of a heptatonic scale (Shepard, 2010). These "sweet spots" leave only two "valleys" for II and VII degrees to close the gaps and fill the octave with tones, distributed by the diatonic principle. Hence, *octave equivalence "resonates" with triadic genesis*. Each of the pitch-classes in a heptatonic mode receives its unique set of melodic and harmonic identifiers. The importance of this can hardly be overestimated in a mode that affords 5,040 dyads. Such diversity requires very intense chunking and high levels of compression, which translates into very complex syntax.

***Octatonic modes*** do not constitute a widespread scheme of tonal organization in world's music—even more marginal than hexatonic modes. The most common are 2 symmetrical (non-diatonic) modes: "tone-semitone" (A-B-C-D-Eb-F-F#-G#) and "semitone-tone" (A-Bb-C-Db-Eb-E-F#-G), both invented during the Romantic period of classical music, actively explored by Liszt, Glinka, and Rimsky-Korsakov, and favored by Stravinsky (Taruskin, 1985). The primary reason for using these modes is harmonic: they provide a number of consonant chords to form harmonic progressions that differ very much from those generated by conventional functional harmony in Western keys.

Technically speaking, the octatonic chords are modal, featuring low harmonic tension, and the symmetrical structure of these modes makes gravitational gradation of their pitch-classes nearly impossible (Kholopov, 1988). Yet another octatonic mode, based on the so-called Alexandrian pentachord (C#-D-E-F-G-Ab-Bb-C), is often named after Shostakovich, although other composers, like Szymanovsky, used such modes earlier than him (Dolzhansky, 1962). There are very few uses

of octatonic modes, reported in folk music: thus, the Istrian scale is sometimes implemented as a symmetrical octatonic row, such as D-E-F-G-Ab-Bb-Cb-Db (Marušić, 2007).

***Hypermode*** is a method of reducing tonal tension in diatonic music and avoiding chromatic alterations by joining together a few diatonic trichords, tetrachords, or/and pentachords, while employing non-octave equivalence and thereby generating systemic “false relations” (see “multitony”) between the lower and upper registers of the ambitus that usually spreads over 10-14 pitch-classes (Nikolsky, 2016f). False relations are usually avoided within the same musical phrase. The term “hypermode” was proposed by Pashinian in reference to the compositional method of chaining a number of conventional “sub-modes,” whose pitch-classes mostly coincide—except a few non-octave equivalencies, so that if to listen to the entire hypermode from its lowest to its highest degree, the scale appears to contain modulations from one “sub-mode” to another (Pashinian, 1973).

In essence, hypermode “diatonisizes” chromatic alterations by *placing them in different registers*. This peculiar method of tonal organization originates from the ancient Greek *Systema Metabolon* that united 3 tetrachords to avoid *diezeuxis* (a disjunction between two middle tetrachords), thereby producing a row of 11 tones: A2-B2-C2-D2-E2-F2-G2-A3-Bb3-C3-D3, with one false relation—B/Bb (Kholopov, 2006). Contrary to the widespread belief that stemmed from confusion over the historic transformations of the term “mode” (Cazden, 1971), ancient Greek music was built on equivalence of not octave but 4th. Thus, Aristoxenus described “modulation by an octave”—which indicates *octave inequivalence* (Hagel, 2009). The Greek naming scheme for the pitch-classes of a music system did not correspond to the octave equivalence, instead reflecting the equivalence of 4ths (West, 1992). The fact that joining two tetrachords made an octave was a coincidence and not a principle of ancient Greek *melopoeia* (art of making melody): Greeks simply did not have a name for intervals larger than 5th (Kholopov, 2006). Octave species were by-products of conjoining the tetrachords (occasionally, pentachords) within a given key—for this reason, keys had names, whereas species remained nameless (Gombosi, 1951).

Music systems that descended from the Greek one inherited the non-octave design: Byzantine *oktōēchos*, Daseian notation, Persian *dastgah*, Arabic *maqam* and its Eastern derivatives (Turkish *ma.k.a.m*, Central Asian *shashmaqam*, etc.)—they all feature non-octave naming scheme and tetrachordal/trichordal principle of music-making. Hypermode was transformed from a music system into a mode for creating melodies in the practice of Byzantine *hexáechos* (Gr. ‘6 modes’)—i.e., a smaller version of the principal system of *oktōēchos.* The *hexáechos* G2-A2-B2-C2-D2-E2-F2-G2-A2-Bb2-C3-D3 was adopted by the Russian Orthodox Church as the *obykhodnyi* mode, comprised of 4 trichords, named “simple,” “dark,” “light,” and “ultra-light” and featuring 3 modal inversions: major (from G), minor (from A), and diminished (from B) (Schidlovsky, 2009).

Similar design (although with different sub-modal divisions) characterized implementations of *hexáechos* in other Churches: Armenian, Georgian, Bulgarian, as well as Western, where it received more sophisticated arrangement in the Daseian scale. The latter contained not 1 but 4 false relations—augmented octaves Bb/B, F/F#, C/C#, broken apart by tetrachords, plus optional chromatic inflection E/Eb (Spiess, 1957). Hypermodal design was favored by Christian authorities, who sided with Platonic condemnation of chromatic music as overly sensual and therefore sinful. Hypermodal technique provided a way around chromatic alterations while supporting a greater variety of expressions than those that the strictly diatonic music could convey. In Eastern Europe and the Caucasus, once established in plainchant, hypermode made its way to secular genres, including traditional folk music (Rudneva, 1994). Islamic music cultures of this region (Azerbaijan, Turkey, as well as neighboring Syria and Iraq) also employ hypermodal organization through their shared ancient Greek heritage. Thus, maqam Saba commonly employs the combination of *jins* (i.e., subset of 3-5 adjacent pitch-classes) of Saba, Ajam, and Nikriz, forming 10 pitch-classes (D-E𝄳-F-Gb-A-Bb-C-Db-E-F) that generate 2 false relations (Farraj & Shumays, 2019).

# Melodic typology

***Melodic intonation*** is a concept introduced by Boleslav Yavorsky for the analysis of music as part of his theory of "musical speech," defined by him as the elementary unit of music structure that binds its semantic content to a similar verbal intonation, based on their shared prosody (Yavorskii, 1908). Boris Asafyev further elaborated Yavorsky's theory by viewing musical intonation [Rus. "*intonatsiya*"] as a semiotic "tone-cell," characterized by 3 parameters: intervallic size, melodic direction and relative euphony—altogether defining the semantic value of a given intonation by means of perceptual associations, selected and conserved by cultural conventions (Asafyev, 1952). Here, melodic euphony was understood as comparative "melodiousness" that reflected psycho-physiological ease of singing of a given interval, and the extent of cultural preference for it. In essence, "melodic intonation" to melody is what "chord" is to harmony (Mazel, 1982).

In this framework, a musical mode constitutes a "container" of intonations selected by the performance practice for expressions that are most important for a given musical culture (Tull & Asafyev, 2000). This was a convenient model for understanding the tonal organization of traditions based on oral transmission as well as for notated "art music" (Bytchkov, 1987). It is this understanding of "musical intonation" as a semiotic sign, forged in a community of music-users, that was adopted by musicologists of countries of the former Soviet bloc and became the cornerstone of the musicological intonational analysis of all forms of music (Zemtsovsky, 1980). In essence, "intonation theory" should be viewed as a restoration of the semiotic musicology, developed by the 18-19th centuries *Formenlehre* scholars such as Kirnberger, Koch, Riepel, and Reicha, who focused on investigation of melody as the most expressive aspect of music. This line of study was interrupted by the shift of interest of Western musicologists to harmony (with very few exceptions, like Kurth), in quest of underlying principles of morphology of music (Baker, 1976). Because Eastern European and Asian music has been strongly influenced by modality and melodic orientation (numerous cultures completely lack multi-part musical traditions—see (Nikolsky & Benítez-Burraco, 2022)), researchers of their music remained interested in the tonal organization of melody. This should explain why Asafyev's theory was adopted by musicologists in the East but not in the West.

This "melodic intonation" should not be confused with the "performance intonation"—i.e., the acoustic accuracy of playing or singing in tune, according to the standards of a given music system and the perceived need in expressive exaggerations (Leedy & Haynes, 2001). However, "performance intonation" is closely related to the theory of melodic intonation, since the rules of expressive "intoning" are shaped by cultivation of specific "tone-cells." Conjoining 2 pitch-levels within a melodic intonation reflects a preferred method of tone generation, articulation, and resonance—thereby enabling the comparative study of "ethnophony" (i.e., music system of a specific ethnos; Kvitka, 1971). Furthermore, melodic intonation inherently determines melodic typology, both structurally and semantically, by setting a melodic momentum, since a single tone is melodically meaningless—only its repetition or change can specify its semantic framework (Zemtsovsky, 2012). Intonational analysis is indispensable for adequate identification of musical modes, because it is the only means of uncovering modal *homonymy*—i.e., melodic structures that only superficially resemble each other (e.g., Chinese and Scottish pentatonic intonations), while being governed by different modal principles (Zemtsovsky, 1974). There are 4 general types of intonation: anthropophonic, naturophonic, organophonic, and metrophonic.

***Anthropophonic intonation*** characterizes timbre-oriented music and resembles linguistic phonemes. It was conceptualized by Alekseyev as the expressive use of anatomical capacities of the human body to generate sounds, following an impulse to vent out certain emotions, direct one's thought towards a desired goal, or engage a partner into a mutual activity (Alekseyev, 1993). This includes "singing for oneself" to accompany some solitary activity, playing "musicking" games like Inuit *assalalaa*, or performing important religious rites. Typical production involves glottal, nasal, wheezing, croaking, or subglottal utterances within a selected frequency range. The resultant melodic intonation is not "pure pitch" generated by "true vocal folds," but "dirty pitch," modulated by "false vocal folds." Although such

inonation can appear to possess a pitch value or to follow a specific melodic shape, in reality, these outcomes are unintentional by-products of the production of timbre-classes in a way, essentially similar to constructing syllables by combining the vowel phonemes (pitch component) with the consonant phonemes (noise component).

***Naturophonic intonation*** characterizes timbre-oriented, ekmelic, and khasmatonal music. In contrast to anthropophonic intonation that serves to reflect a certain *internal* state (like infant's vocalizations), this intonation aims at reproduction of naturally occurring sound sources (the onomatopoeic model) that are *external* to the sound-maker (Nikolsky & Benítez-Burraco, 2022). Imitation of environmental sounds can be done by vocal or instrumental means—using such phono-instruments (see below) as decoys. Unlike imitations by means of sophisticated multi-pitch musical instruments (e.g., flute imitations of bird calls), these imitations are timbre-oriented and strive for maximal accuracy of reproduction. However, once adopted by a music culture, naturophonic intonation can receive a creative treatment within a particular music genre (Sheikin, 2002). One intonation can be combined with another to form a compound, whose expression then would combine the expressions of both constituents—e.g., onomatopoeic imitations on the Jaw Harp (Alekseyeva, 1986).

> ***Phono-instrument*** is a sound-producing tool, manufactured for some common application other than music-making, but used for musicking, and appreciated for its capacity to generate a particular sonority (Yesipova, 2008). The most common phono-instruments are whip, cane, flask, jingle-bells—the everyday accessories of shepherds that are also commonly used by children as a toy (Mazepus & Galitskaya, 1997). The term "phono-instrument" was introduced by Sheikin (Sheikin, 1996) to supplement the Sachs/Hornbostel structural classification of musical instruments by the classification of their functionality, where "phono-instruments" represent the archaic forms of musicking—preceding the invention of instruments, designed specifically to generate a particular type of music (Sheikin, 2002).

***Organophonic intonation*** characterizes ekmelic and simplest emmelic forms of music (e.g., oligotonal). This term was introduced by Sheikin in reference to those music traditions that adopt the sound of a particular musical instrument as a default model for melody-making in general, including vocal music (Sheikin, 2002). Thus, tambourine constitutes a sonic ideal for Chukchi, whereas musical log—for Nivkhi. Such idealizations often distinguish one indigenous music culture from another. The term "organophonic" is derived from "organology"—the discipline that studies musical instruments. Sheikin insists that through organophonic model, every indigenous musical culture defines its favorite "acoustic medium" and perpetuates it in the tonal organization of some central music genre: usually ritual (e.g., wedding, burial) and/or epic (heroic epos, tales). Such genres become canonized and carefully preserved. Once an ethnicity invents or borrows an array of pitch-oriented musical instruments, its music system starts shifting towards pitch-based tonal organization and collective music-making (a good example is the Chukchi culture—see (Sheikin, 2018)). Sometimes, one organophonic model replaces another throughout history: e.g., Hellenic lyra was replaced by Hellenistic aulos for the ancient Greeks (Hagel, 2009).

***Metrophonic intonation*** characterizes music systems that started emerging during the Bronze Age. Such systems are distinguished by having a math-based music theory, formal music education, professional music occupation, and some form of musical notation. The term "metrophonic" was proposed by Nikolsky and Benítez-Burraco (Nikolsky & Benítez-Burraco, 2022) in place of the term "metric" originally used by Beliayev in reference to "the system of metric temperament" (Beliaev, 1963). This renaming was intended to keep the names of all intonation types uniform and avoid the confusion with the conventional meaning of the term "metric" in musicology (see above). Beliayev had in mind something completely different from metro-rhythmic organization: a method of constructing musical instruments by placing the holes on woodwind instruments or frets on string instruments in *correspondence with the relative size of common interval-classes*, according to the incremental measuring of linear distances

between those holes/frets (Beliayev, 1990). Hence, the word "metric" here was used in the same sense as "metric system" of measurements. Beliayev inferred his metric temperament from comparative measurements of various Eurasian musical instruments that belonged to the same organological class (Beliayev, 1931) and from the comparative analysis of the documented historic tunings (Beliayev, 1971).

According to Beliayev, in many folk music cultures, instrument-makers empirically figure out that dividing a string in half generates the octave and in 3⁄4—the interval of a 4th. With these two interval-classes in place, the intervallic value of a 5th can be inferred from an octave by inverting the interval of a 4th. Once the "metric" equivalents of 5th and 4th are established, it is easy to define the value of a whole step by deducting the 4th from the 5th. Dividing this whole step in halves gives the "metric" value for a semitone. This basic arithmetic is widely used for constructing musical instruments across the Eastern Eurasia. Sheikin confirmed that such "metric method" was known in some of those Siberian indigenous cultures that retained timbre-oriented and ekmelic traditions—probably as a result of imitating the construction of Russian, Chinese, and Korean musical instruments (Sheikin, 2002).

Kvitka noted that the same principle of construction was evident in some Neolithic flutes discovered by archaeologists (Kvitka, 1971). Beliayev's model allows a modern scholar to infer the common interval-classes of the oldest Paleolithic bone flutes (Nikolsky, 2015b). What is most important, once musical instruments built according to a "metric principle" are adopted as organophonic models for a musical culture, its other traditions (including vocal) acquire metrophonic intonation. Such development is evident in the history of ancient Greek music (Hagel, 2009) and was reported in other musical traditions in the Caucasian region (Kushnaryov, 1958). Obviously, metrophonic intonation can be implemented in a range of music systems—from mesotonal to full-fledged tonality—where on the one pole we have *implicit*, orally transmitted music theory that *describes* common practices, whereas on the other pole, there is *explicit* formal music theory that *prescribes* grammatical rules. The boundary between both is laid by the notion of error: folk music usually does not support it. The first documented reference to musical error belongs to ancient Greek culture, as evident from the opposition of "ekmelic" and "emmelic" sounds by Greek theorists (Lippman, 1964).

***Motif*** is usually defined as the shortest subdivision of a theme that still maintains its identity as a musical idea—distinguished primarily by its melodic structure, but often with the contribution of a characteristic harmonic or/and rhythmic pattern (Drabkin, 2001b). The concept of motif was forged in the linguistically oriented German musicology of the 18th century (Mattheson, Koch), but the term itself originated in Italy, where it referred to the thematic impetus of an aria (from Lat. *moveo*) (Bobrovsky, 1976). In the musical intonation theory, motif is viewed as the memorable harmonic and rhythmo-metric arrangement of a salient melodic intonation, designed to carry a specific expression, and often supported by secondary intonations (Tiulin, 1969).

"Catchiness" of an opening motif determines the expressive capacity of the entire theme and, thereby, an entire music work. The unveiling of an expressive music work occurs through the motif-work, where motifs are fragmented, extended, contrasted, or derived from each other. Music with unclear motif-work usually appears babbling (which might be appropriate for passages or bridges). The size of a motif is determined by the semanticity of its intonation(s): a single laconic energetic intonation forms brief motifs that are 3-5 tones long (e.g., Beethoven's 5th Symphony), whereas the combination of 2 or more contrasting bright intonations can generate complex motifs that might be mistaken for a phrase (Tchaikovsky's 1st Piano Concerto, the opening theme in Db major).

***Phrase*** in music is usually defined as a syntactic-semantic unit, greater than motif, but shorter than sentence. Modern English-speaking music theorists pretty much left the issues of phrasing and musical phrase behind as too controversial—noteworthy, the Grove Dictionary covers the "phrase" in just 3 sentences. However, in the history of Western music, the notion of "phrase" inseparable from the notion of tonality. The term "phrase" in reference to music was introduced in 1722 by Couperin as a basic meaningful unit of a musical composition, not always marked by a pause and therefore needing a special notation sign. Rousseau, in his dictionary (1768), provided a more elaborated definition: "Phrase is an

uninterrupted harmonic or melodic progression that carries a more or less complete idea and ends with a more or less perfect cadence." This is in agreement with the etymology of the term: from Gr. *phrasis* ['declaration'].

Mattheson, Kirnberger, Schulz, and Koch forged the convention of defining "phrase" as a 2-bar structure, tying it to meter to distinguish it from a 1-bar-long motif and a 4-bar-long sentence. This metric reference was further elaborated by Catoire and Taneyev, who stressed the inadequacy of strictly metric definition of a phrase, pointing to the contribution of thematicism that could shape a phrase in an unconventional way, e.g., as a 3-bar unit (Lavrentyeva, 1981). The boundaries of phrases are determined by the termination of harmonic, melodic, and rhythmic tension with relaxation, thereby forming "resolution" of tension in a cadence, the end of which is marked by injection of a momentary pause (a.k.a. "caesura") in order to break the metric regularity of a musical movement (Tiulin, 1969).

The existence of phrases in music has been experimentally demonstrated—psycho-physiologists call musical phrases "music closure positive shift" and measure them using event-related brain potentials (ERPs) and event-related magnetic fields (ERFs) (Nan et al., 2006). Musicians have been shown to process musical phrases in a structured manner, similar to language, in contrast to non-musicians who detect primarily discontinuities in the melodic flow in form of caesuras rather than actual pauses (Neuhaus et al., 2006). A follow-up cross-cultural ERP study revealed that non-musicians rely on bottom-up parsing and cultural familiarity with melodies, recognizing phrases about 3-5 times slower than musicians, who achieve higher rates due to the effective top-down processing (Nan et al., 2009). Furthermore, musically trained and untrained listeners both show consistent emotional response to the detected phrases, where phrase boundaries act as points of perceptual salience (Livingstone et al., 2009).

***Sentence*** in music is recognized primarily as a section of the simplest music form—a so-called period, defined as: "a musical statement terminated by a cadence or built of complementary members, each generally 2-8 bars long and respectively called 'antecedent' and 'consequent'" (Ratner, 2001). The traditional (from the 16th century on) equation of musical "period" with the linguistic sentence is responsible for a great confusion over the musical "sentence"—especially amongst English-speaking theorists. If German theorists qualified musical sentence as "Satz," a couple of which would comprise "Period," French theorists correspondingly named both "Phrase" and "Clause," Russian theorists—"Fraza" ["phrase"] and "Predlozheniye" ["sentence"], yet English-speaking musicologists referred to both by the same name—"phrase" (Benjamin et al., 2015).

The confusion was exacerbated by the preoccupation of musicology in English with rigid schemes of music form, taken in isolation from the thematic organization. While European musicologists recognize motif and phrase as *thematic* distinctions in contrast to sentence and period, both of which are considered *formal* distinctions, their English colleagues generally do not draw this distinction. A short anonymous entry in the Grove dictionary tells that "sentence" is "a term adopted from linguistic syntax and used for a complete musical idea, for instance a self-contained theme" and "has much the same meaning as 'period,' though it lacks the flexibility of the latter term, being restricted to dance-like and other symmetrically built musical statements." This clearly inadequate and confusing definition reflects the general lack of interest in the English-speaking music theory to the rhetorical tradition and, broader, musical semiotics.

In practice, musical phrases more often than not form a 4-bar sentence that can be stand-alone—albeit the relation of phrase and sentence is far from clear-cut: a complex phrase can approximate a sentence, or a complex sentence can approximate a period (Tiulin, 1969). What is important, in such unclear cases the distinction between phrase and sentence comes from *thematic* and *harmonic* analysis rather than stereotypical structural schemes, taken in isolation. In other words, syntactic divisions in music are determined by rhetorical compositional principles that take into consideration typologies of expression.

One of the main means of securing the compositional integrity of a musical sentence is harmony—the relation of cadences at the phrasal ends. There is experimental evidence that long-distance key dependencies between the sentences are indeed perceived by the musically trained and untrained

listeners (Woolhouse et al., 2016). Such dependencies are qualified as "tail recursion" (Rohrmeier et al., 2014) in the theory of a generative syntax of tonal harmony and are believed to be implemented at multiple hierarchical "levels of harmony"—in a way similar to syntactic structures in languages (Rohrmeier, 2011). The available ERP and behavioral evidence indicate that listeners process phrasal ends in musical sentences differently, depending on their perceived harmonic stability or instability (Koelsch et al., 2013).

***Melodic inclination*** is an important parameter of melodic and harmonic expression that is often overlooked by modern Western music theorists (see (Nikolsky, 2017). Throughout the course of the 16th century, Western classical music developed the concepts of major and minor triads, which after formation of tonality were elaborated into major and minor keys (Lester, 1989). Both were regarded as generic categories—something akin to "majorness" and "minorness" (McKinney, 2016). To address this distinction, Hugo Riemann introduced the term *Tongeschlecht* (Ger. 'Tone gender'). It was translated into English as "mode," or "tonal genus," or "Clang genus" (Riemann 1896), but neither of these translations have been adopted in English musicology since then. This is especially surprising, provided that the distinction between major and minor is readily heard and experienced as happy versus sad, both in harmonic (Bakker & Martin, 2015) and, to a lesser extent, in melodic implementations (Halpern et al., 2008)—possibly presenting one of the very few musical universalities (Virtala & Tervaniemi, 2017).

German theorists subsequently generalized the *Tongeschlecht* distinction into a dualistic theory, stressing its biologic connotation: major as the embodiment of masculine, active, positive qualities; and minor as the embodiment of feminine, passive and negative qualities (Rothfarb, 1979). Ernst Kurth formulated a melodic implementation of *Tongeschlecht:* according to him, *major* scale entailed the inherently active idea of *ascension* toward the upper tonic—as opposed to *minor* scale, whose passivity was manifested in its propensity to *descend* (Kurth, 1931). This was not a scholastic generalization. The statistical analysis of the corpus of classical instrumental themes demonstrates that minor themes are on average lower in pitch than major themes and have smaller intervals (Huron, 2008). This agrees with Mazel's finding that major keys are characterized by the ascending resolutions in cadences, in contrast to minor keys (Mazel, 1952). Ascending major scales are perceived as more positive than descending major scales, while descending minor scales—as more negative than ascending minor scales (Gerardi & Gerken, 1995). Unlike major scales, ascending minor scales are perceived as awkward in comparison to descending minor scales (Collier & Hubbard, 2001). Musicians and nonmusicians consider ascending scales brightening as they approach the top, whereas descending scales darkening (Collier & Hubbard, 2004). The semantics of major and minor directionality very well might have originated in speech—the spectra of the intervals that distinguish major modes resembles the spectra of *excited* speech, whereas that of minor modes resembles *subdued* speech (Bowling et al., 2010).

Asafyev advanced Kurth's theory by introducing the term ***inclination*** [*nakloneniye*]—borrowed from linguistics, where it referred to the distinction between an active and passive voice sentences—in a sense of directing the melody up (majorness) or down (minorness) towards a target pitch-class (Tull & Asafyev, 2000). Aleksei Ogolevets developed a methodology to estimate the index of "majorness" and "minorness" of a particular mode, based on the distribution of its tones by the circle of 5th in relation to its tonic (Ogolevets, 1941). The theory of melodic inclination was co-developed with the theory of musical mode in the exploration of folk music. Feodosii Rubtsov investigated the historic development of classical music and folk music in Russia and introduced the third inclination—***neutral***, peculiar to folk song and characterized by neither major, nor minor 3rds in relation to tonic (Rubtsov, 1964). Indeed, neutral tonic thirds are exceedingly common for many folk music cultures.

Yet another known inclination is ***diminished.*** It is especially pronounced in the so-called Locrian mode (B-C-D-E-F-G-A), which can be characterized as "ultra-minor" due to its tonic tritone in comparison to the tonic 5th of regular minor (Ogolevets, 1941). Diminished triad has been experimentally found to trigger negative associations in listeners, such as danger or violence (Smith & Williams, 1999). Accordingly, music in Locrian mode, where the diminished triad executes the role of the tonic, is perceived as angry rather than sad, in distinction from minor modes (Trochidis & Bigand, 2013). Locrian

music is associated with loathing, boredom, and disgust (Straehley & Loebach, 2014). Locrian mode is easily detectable and distinguished from major and minor modes by musically untrained people (Ramos et al., 2011). Diminished inclination can be found in a variety of intervallic typologies: diatonic Locrian, common in South Balkans; symmetrical chromatic octatonic (see above); mesotonal Istrian (B-C-D-Eb-F-Gb); and non-octave hypermodal, such as Russian *ukosnyonnyi obikhod* (B-C-D-E-F-G-A-Bb-C), common not only in ecclesiastical but also in folk music (Rudneva, 1994).

***Augmented*** inclination exists in the archaic *Setu* mode, found in Estonia (Ambrazevičius & Pärtlas, 2011) and Siberia (Kalkun & Oras, 2014). It features 2 salient augmented triads (C-E-G# and Db-F-A in the scale of C-Db-E-F-G#-A), where the lower triad is usually tonicized, supporting 2-part singing in parallel major 3rds. Yet another case of augmented inclination constitutes the so-called *whole-tone scale* (C-D-E-F#-G#-A#). It is characterized by the reduction of tonal tension due to the absence of semitones and the resulting perfect uniformity of steps and absence of any other triads but the augmented ones (Kholopov, 1988). This method of tonal organization was invented by such Western composers as Rossini, Schubert, Berlioz, and Glinka to emphasize exotic or strange characters. They used whole-tone scale episodically, limiting it to melody alone and harmonizing it according to harmonic conventions of Western tonality. Romantic Russian composers (Dargomyzhsky, Borodin, Rimsky-Korsakov) started employing a whole-tone mode to generate unusual harmonies, characteristic of supernatural or inhuman imagery, which prompted Modernist composers (Rebikov and Debussy) to pose whole-tone modality as an alternative to the major-minor system (Andrews, 2001). Structurally very similar scales—*equitonic heptatonic*—have been reported in some non-Western music cultures: Fataleka Au-Ero panpipe-bands from Solomon Islands (Kaeppler et al., 2013), Chopi xylophone tradition from Uganda (Haddon, 1952), and Nzakara music of Central Africa (Arom, 2004). The step-equivalence of such music enables parallel progressions of the adjacent degrees, moving by parallel major 2nds.

# Textural organization

***Voluminousness*** is a peculiar aspect of texture in music that for some reason has not been recognized in music theory until the second half of the 20th century. This term was introduced by Nazaikinsky to refer to the quantitative contrasts in performance of the same part in texture: ***solo*** as opposed to ***group***, and group as opposed to ***tutti*** (Ital. 'all', i.e., the entire ensemble) (Nazaikinsky, 1982). These 3 categories are fundamental for orchestration and instrumentation (Kreitner et al., 2001), as well as for the arrangement of choral music (Ades, 1966). In practice of scoring, there are terms reserved for indicating the number of performers required to carry out a specific part: "*a due*," "*a tre*," and "*a quattro*" being most common—and the indications to perform by an entire orchestral group: *archi*, *legni*, and *ottoni*. Most of such quantitative gradations are quite easily distinguished from each other by the minute discrepancies in phase between the simultaneous sounds produced by the participants—which is known as "chorus effect" in electronic music. The contrasts in voluminousness are often used creatively within a composition (e.g., the Baroque Concerto Grosso is based on the opposition of *tutti* and *soli*) or in a responsorial setting to increase the contrast between the call (*proposta*) and the response (*riposta*). Such compositional contrasts are not limited to Western classical music. Thus, the Arabic genre of Nubah is based on the same contrasts between *soli* and *tutti* (Pacholczyk, 1993). Similar principles are often at work in the arrangement of folk choral music (Jordania, 2006).

***Part*** (from Lat. *partio*—'divide') is the elementary unit of music texture, comprised by a continuous exposure of the same thematic material within the same register, if it retains the same textural function (e.g., melody, counter-melody, bass, accompaniment of a certain kind) in relation to the other simultaneously engaged layers of texture (Kholopova, 2002). The concept of "part" is often convoluted by the contribution of voluminousness. The word "part" usually refers to the notation of music that is supposed to be played by an individual performer (e.g., "first violin part" or "piano part"), which might or might not coincide with the notion of the "textural part." Thus, all performers (i.e., many different

*performance* parts) might be required to play in perfect unison, generating a single *textural* part. Vice versa, a single *performance* part in orchestral music can generate multiple *textural* parts, if an instrumentalist can make pitches with both hands (e.g., piano, harp, vibraphone). Defining a part in a Western composition might be tricky, especially in scores for the instruments that can play many tones at the same time, most notably piano solo—since parts might not be clearly marked by the composer (usually, parts are indicated by adding stems that point at the opposite directions). This is especially common for the technically elaborated accompaniments that often layer out "sub-parts" by stressing the bass line and bringing out the uppermost notes (Chopin—Etude F Minor op.10 No.9) .

***Voice*** is the "subatomic" particle of a chord in a homophonic texture, usually generated by "voicing"—i.e., tracing the melodic continuity between the constituent tones of the adjacent chords (most commonly, the upper tone forms a voice line, as in Chopin's Prelude E Minor No.4). In homophonic choral and instrumental chamber music that are based on the ongoing succession of chords, voicing has to follow strict rules, often determined by psychoacoustic factors (Huron, 2001). In piano, guitar, and harp music, chords often have to be "voiced"—i.e., their utmost upper (sometimes, lower) constituent tone must be emphasized (by means of dynamics or articulation, e.g., legato). Within this context, "textural voice" is understood as equivalent to the "voice" of a singer (the term "voice" comes from Lat. "*vox*"—'vocal'). A single singer is confined to monophonic sound production. It is as though a chord progression in a piano composition is distributed between the singers (1 singer per 1 note of a chord). In performance practice, complex textural units, such as chords and double-notes, have to be split into voices to optimize phrasing and articulation. However, the words "part" and "voice" are sometimes used indiscriminately, especially in popular music.

***Monophony*** is such an arrangement of texture, where no more than a single pitch- or timbre-class sounds at any point of time. In Western classical music, plainchant is an example of strict monophony. Music for a wind solo instrument usually produces monophony—except in the Western avant-garde music that makes use of mutiphonic techniques, such as singing while playing, overblowing, or using unorthodox fingering on woodwinds. Singing while playing is also used in some instrumental folk indigenous traditions, such as Bashkir *kurai*. In the absolute majority of cases, solo singing is monophonic—with a notable exception of the Mongolian and Tuvan traditions of throat singing, where a single singer generates two discrete timbrally-contrasting pitches at the same time.

***Monody*** is often misunderstood as synonymous with monophony, when taken outside of the adequate historical context (Fortune & Carter, 2001). The term "monody" (Gr. *monodia*—'singing alone') was introduced in ancient Greece to refer to lyric songs, traditionally performed with the self-accompaniment on a string instrument—and later applied to Italian arias that featured similar performance settings in an attempt to revive the ancient Greek lyrical tradition (Palisca, 1960). Already in ancient Greek music, monody was not monophonic: singing was supported in a free fashion rather than dubbing a vocal melody note-for-note, and most probably used simple figurative accompaniment at times (West, 1992). In the Renaissance arias and madrigals solo, the accompaniment for sure differed from the melody, often featuring chords and a bass line that was fixed in notation (Tomlinson, 1981). In the 17th century, such arrangement was theorized as *basso continuo* (a.k.a. "figured bass") and reflected in scores by a special numerical notation. Confusingly, compositions titled as "sonata for flute solo," in reality were performed by 3 musicians: a soloist, a bass-player, and a chord-player. Obviously, the resultant texture was not monophonic but homophonic (see below). The only nuance that distinguished such monody from the normative homophony was that monodic accompaniment was improvised (just roughly following the notated "general bass") on unspecified musical instruments. This is in contrast to the prescribed notes of melody, arranged specifically for a given instrument or vocals. In non-Western traditions, monody is typical for epic singing and for classical traditions of Western and Central Asian civilizations that developed formal music theory and notation. Monody can be found in some oligotonal, ekmelic, and even

timbre-oriented cultures, where solo singing is accompanied on a rhythmic instrument, such as tambourine (Sheikin, 2002).

***Heterophony*** is such an arrangement of texture, where a single melody is performed by more than one performer, and at least one performer occasionally deviates from others in rendering this melody, so that from time to time, 2 or more simultaneous pitches are generated, forming harmonic "patches" in otherwise monophonic model (Nikolsky, 2018). The etymology of this term reflects its opposition to monophony: Gr. *Monophonia*, 'sounding as one', versus *heterophonia*, 'sounding as others'. The essence of heterophony is the collective rendition of a preexisting monophonic melody by singers or/and instrumentalists, who differ in their capacity to produce pitches: e.g., for one singer a particular tone of the melody is too high or too low, requiring to replace it with the nearest available pitch that harmonically agrees with another singer, who reproduces the same melody properly.

Musical instruments are even more prone to transform a monophonic melody into heterophonic. The difference in sound production often generates pronounced differences in articulation: e.g., a plucking sound quickly decays, in contrast to a sound produced by blowing, so in order to play a long note of a loud melody, a harpist would have to play some extra pitches (since repeated striking of the same string would attenuate the sound), while a flutist holds the same pitch. Such mismatches will generate heterophony.

Alternatively, heterophony might emerge as a result of creative input on part of performers, trying to make the melody more expressive (Swan, 1943). Heterophony is often mistaken for polyphony (see below), especially in relation to monody. This is because heterophony is opposed to monophony, whereas polyphony is opposed to homophony—but both pairs are *not* opposed. In fact, "monophony" resembles "homophony" by its underlying idea of integration, and "polyphony" might resemble "heterophony" by its idea of differentiation. In practice, hardly anyone would mistake monophonic texture for homophonic. But on the other pole, a complex heterophonic texture might make a "polyphonic" impression (e.g., a large-scale performance of Nouba). In such cases, it is important to understand the "etiology" of heterophony.

Stumpf found the term "heterophony" in Plato's "Laws," where it referred to the pitch and rhythm discrepancy between the vocals and the lyre performing the very same tune (Stumpf, 1897). Stumpf used this term to categorize a peculiar arrangement of a Thai music work, whose parts generally followed the same melodic contour, while differing in detail, so that minute discrepancies would meet again in unison (Stumpf, 1901). Adler generalized Stumpf's interpretation of Plato's term, adopting it as the principle of arranging texture that is alternative to polyphony and homophony (Adler, 1908). Adler pointed out that some music cultures, such as Siamese, Japanese, Javanese, and especially Russian, favored heterophonic arrangement over others. Hence, the clearest difference between heterophony and polyphony is in the distribution of thematic material within a texture. Heterophony is bound to a single material, presented more or less in sync (the greatest deviations of timing constitute no longer than a beat or two and are fragmentary, as in Hebrides psalms—see (Cooke, 2001)). Although polyphony can be imitative and distribute a single theme between multiple parts, their distribution is deliberately divorced in time, so that the imitation usually starts a bar or two later (occasionally, half-bar, but hardly ever 1 beat) and stays until the end of a music work.

***Polyphony*** is such an arrangement of texture, where two or more parts *each* carry out a melodic line, thereby generating a continuous progression of various harmonic intervals, according to a set of harmonic rules. In Western classical music, such rules are specified by the theory and technique of ***counterpoint*** (from Lat. "*contra*" + "*punctum*," i.e., 'pricked against'—in reference to a method of composing music, where each tone of one melody is defined against a simultaneously sounding tone of another melody). Although the technique of counterpoint often relies on notation: singers can generate new parts by looking at the notated part (Bukofzer, 1940), or composers sketch parts on an erasable tablet (Owens, 1998)—a number of non-Western folk music cultures developed equivalent techniques of generating and harmonic matching of parts without any notation, exclusively by ear, usually together with a well-known

partner, a friend or a family member (Jordania, 2011). A well-known example are folk rounds (e.g., “Three Blind Mice”).

Traditionally, Western polyphony is classified in a number of types (Protopopov, 1978):

1. ***contrasting polyphony*** (each part contains a melody, based on its own thematic material, all synchronously combined—e.g., the genre of chorale prelude),
2. ***imitating polyphony*** (parts diachronically reproduce the same theme—e.g., fugue or round),
3. ***ostinato*** (one part keeps repeating the same melodic formula, while other parts keep varying a similar or contrasting material, continuously renewing it—e.g., passacaglia),
4. ***diaphonia*** or ***fauxbourdon*** (one part carries a thematic material, whereas another part or parts consistently dub it in some interval with occasional melodic deviations, joining in cadences—e.g., organum),
5. ***drone*** or ***bourdon*** (the simplest case of polyphony, when one part carries a melody and another part sustains a pedal tone or tones, like bagpipe, which can occasionally move to another pitch level to support a melodic modulation—e.g., the *ison* technique of the Byzantine chant and the derivative Balkan traditions of chant and folk music—see Koço, 2015.

These types can be combined to form more complex textures. Still more complex polyphonic textures can be generated by mixing polyphonic and homophonic textures—as in a fugue with the basso continuo accompaniment (Kholopova, 1979).

Unfortunately, the term “polyphony” has been quite often misapplied to heterophonic and homophonic textures, so a careful examination of the music in question is needed in order to evaluate whether it constitutes polyphony or not. Even the Grove Dictionary entry on “part” erroneously lists “four-part harmony” as belonging to polyphonic music (Drabkin, 2001c)—in reality, music like “barbershop harmony” (a.k.a. barbershop quartet) constitutes homophony.

Even greater confusion has been amongst ethnomusicologists. Some of them refuse to acknowledge that oral transmission can support counterpoint and therefore use terms alternative to “polyphonic” (polivocal, plurivocal, multi-phonic, multi-sonic, diaphonic, and disphonic). Other ethnomusicologists equate polyphony with “multi-part” to include homophony and heterophony. Still others, in the opposite, narrow polyphony down to refer to the contrasting polyphony alone. And many others reject any classification at all, considering classification a Eurocentric cultural bias, detrimental to indigenous cultures, and use the indigenous terms instead, thereby obstructing cross-cultural comparisons (Frobenius et al., 2001). An international ethnomusicological conference dedicated to polyphony coined the following definition: “polyphony is a mode of expression, based on simultaneous combination of separate parts that are perceived and produced intentionally in their mutual differentiation, in a given formal order” (Agamennone, 1996).

***Homophony*** is such an arrangement of texture, where parts are functionally categorized into subordinating (i.e., melody) and subordinated (accompaniment)—in contradistinction from polyphony that does not observe such distinction, so that polyphonic parts are *coordinated* rather than *subordinated* to each other (Kholopov, 1973). This opposition is reflected in the etymology of both terms: Gr. *homophonia*—‘sounding alike’ (i.e., fusing sounds together) versus *polyphonia*—‘sounding different’ (i.e., many sounds, each on its own). Amazingly, this clearcut opposition quite often eludes modern English-speaking music theorists. Thus, the Grove Dictionary paradoxically defines homophony as: “Polyphonic music in which all melodic parts move together at more or less the same pace” (Hyer, 2001a)—clearly, mistaking “polyphonic” for “multi-part” and narrowing homophony to the chorale-like texture. In reality, the typology of homophonic textures is no less diverse than that of polyphony and is not limited to Western classical music (Nikolsky, 2016h):

1. ***parallel mono-chordal homophony***—strict synchronization of all parts, dubbing the same melody in a fixed harmonic interval (similar to polyphonic *fauxbourdon*, but with strict parallel voicing of chords throughout an entire music work—e.g., *Ompeh* music of the Akan people from Ghana);
2. ***modal multi-chordal homophony***—similar to parallel, but featuring different chords that form various harmonic relations (based on the differentiation between a few chordal structures—e.g., Georgian traditional 3-part singing, often inaccurately called "polyphonic");
3. ***functional equi-rhythmic homophony***—similar to multi-chordal, but recognizing inversions of chords and featuring chord progressions, according to the harmonic functionality of chords (introducing the fluctuations in tension—e.g., chorales or barbershop singing, also instrumental music for harmonica or accordion solo);
4. ***functional iso-rhythmic homophony***—similar to above, but featuring asynchronous melodic motion between the parts that manifest melodic differentiation (e.g., the bass part contrasts the treble part by containing longer rhythmic values—typical for multi-string instruments solo (lute) and small ensembles (flamenco music);
5. ***figurative homophony***—the replacement of chords with melodic figurations, based on "broken chords" (e.g., using a progression of single pitches C-G-E-G—a.k.a. "Alberti bass"—instead of the chord C/E/G) to accompany a melody (very common for monodic singing with the self-accompaniment on a guitar and for string ensembles);
6. ***multi-functional homophony***—layering the texture into 3-4 functionally different parts, bound together by the harmonic progressions (1) a melody and an elaborate melodic figuration, layered in multiple voices—e.g., Chopin—the Revolutionary Etude C Minor for piano solo; or, 2) a melody, a chordal accompaniment, a figurative accompaniment, and a bass—common for larger ensembles that mix different instruments, like a Latin American *típica* or Hungarian Gypsy *Táncház* band).

Further complexity is achieved by injecting the polyphonic components in homophonic textures (Kholopova, 1979): a principal melody can be imitated or "answered" (as in responsorial) in another register; the entire homophonic texture can be placed over the drone-like pedal or an ostinato formula in the bass; or a salient melodic figuration can be imitated or contrasted by another melodic figuration. Such "polyphonized" homophonic textures are common in Western popular music. A standard setup includes the leading singer (the principal melody), the background singers (chordal accompaniment or imitations of the melody), the rhythmic guitar (an alternative chordal accompaniment with a sustained rhythmic figure), the lead guitar (a melodic figuration, "licks" in pauses of the vocals, and improvisation solo), the keyboard (pads or an extra figuration), the bass (the ostinato repetitions of a riff), and, optionally, the "horns" (a group of brass instruments or/and saxophones playing "licks," "riffs," or pads). The orchestral music, both in classical and popular genres, can be even more complex.

All such polyphonized textures differ from purely polyphonic textures by containing chords (actual or broken) and featuring a *harmonic pulse*. The latter is formed by the patterns of changes in harmony that occur on stronger metric time (downbeat and half-bars in compound meters). Unlike metric pulse, harmonic pulse usually changes: speeding in climaxes and slowing in cadences (Dubovsky et al., 1965). In English musicology, this is called "harmonic rhythm" (Swain, 2002).

***Accompaniment*** is the function of a textural part, which is supposed to distinguish homophony from polyphony. Unfortunately, English-speaking music theorists often misunderstand this term. Thus, the Grove Dictionary states that "the meaning of the term 'accompaniment' is variable and not subject to rigorous definition," listing the *countersubject* in a fugue or the parts superimposed on *cantus firmus* in strict polyphony as examples of an accompaniment (Fuller, 2001). This erroneous attribution of accompaniment to polyphony stems from the lack of thematic analysis and inability to distinguish

between the subordination and coordination of two different thematic materials. The countersubject in a fugue can be said to "accompany" the fugue's theme (a.k.a. subject) only in the loose sense of occurring at the same time rather than as a musical term. The very name "countersubject" implies that it opposes "subject" and *not* supports it—as the accompaniment does.

The term "accompaniment" comes from Fr. "à" + "compaignier" ('to' + 'companion') and it was introduced as a music term in the 16th century to refer to those parts that were supposed to support the principal melody solo throughout the entirety of a music work (Korykhalova, 1973). In practice, this meant that the accompanying parts contrasted the melodic part by using non-salient unmemorable generic thematic material in an improvising manner (*ad libitum*) and on unspecified instruments, whichever happened to be available to the performers. The melodic part was supposed to feature a bright memorable theme that passed through expressive changes and took advantage of the expressive capacities of a specific instrument or vocals (specified in a score). This functional distinction between the melodic and the accompanying parts was sustained throughout the entire composition. Later advance of piano and organ music enabled a single performer to play melody and accompaniment simultaneously, which made it possible to switch registral positions of melody and accompaniment within the same composition (e.g., rotate the parts). However, still the accompanying material remained "loose" in relation to the "solid" and "fixed" melodic material—no matter in which register both were presented. The same distinction remains in homophonic orchestral music that affords much more frequent registral changes, while sustaining the superiority of a leading melodic line (Skrebkova-Filatova, 1985).

***Isophony*** is such an arrangement of texture that uses brief calls, continuously reproduced by multiple performers without any coordination in timing and pitch, where each participant modulates pitch, timbre, and rhythm according to their likes—altogether producing a "jumbled" effect (Nikolsky, 2018). The concept of isophony was introduced by Nikolsky (2016h) to refer to the peculiar type of texture, generated by collective animal vocalizations, such as howling of a wolf pack, but also encountered in some indigenous cultures, such as the Akia people of the Amazon (Seeger, 2004). What distinguishes this texture from heterophony and polyphony (not to speak of even stricter monophony and homophony) is the complete absence of metric and rhythmic synchronization and coordination in pitch. Everyone reproduces the same call at a comfortable pitch level and at the preferred moment of time, disregarding the sounds produced by other partners. Isophony is designed to expose each participant's identity without enmeshing into the ensemble. Isophony involves the *assembly of individuals*, rather than a single entity ("choir").

# Bibliography

Ades, H. (1966). *Choral Arranging*. Shawnee Press.
Adler, G. (1908). Über Heterophonie. In R. Schwartz (Ed.), *Jahrbuch der Musikbibliothek Peters XV* (pp. 17–27). Edition Peters.
Agamennone, M. (1996). *Polifonie. Procedimenti, tassonomie e forme: Una riflessione "a più voci."* Edizioni Il Cardo.
Agrawal, P. K. (2018). *Indian Culture, Art and Heritage*. Prabhat Pra.k.a.shan.
Alekseyev, E. Ye. (1976). *Problems in the genesis of musical mode (on the example of Yakut folksong): Analysis [Проблемы формирования лада (на материале якутской народной песни): Исследование]*. Muzyka.
Alekseyev, E. Ye. (1986). *Musical intonation in the earliest forms of folklore. The aspect of pitch [Раннефольклорное интонирование: Звуковысотный аспект]*. Soviet Composer. http://eduard.alekseyev.org/rfi/index.html
Alekseyev, E. Ye. (1993). *Speaking and Singing: Prolegomena to Anthropophonics [Пение и говорение. Основы антропофоники]* (p. 19). The Institute of History of Arts at the Ministry of Culture of

the Russian Federation. http://eduard.alekseyev.org/work42.html
Alekseyeva, G. G. (1986). Khomus, play your song for them! [Заиграй им, хомус, свою песню]. In A. Grigoryeva (Ed.), *Music of Russia: Musical creativity and musical life in the republics of Russian Federation [Музыка России: Музыкальное творчество и музыкальная жизнь республик Российской Федерации]* (Vol. 8, pp. 321–332). Soviet Composer.
Ambrazevičius, R., & Pärtlas, Ž. (2011). Searching for the "natural" origins of the symmetrical scales: Traditional multipart Setu songs. *Journal of Interdisciplinary Music Studies*, *5*(1), 1–17. https://doi.org/10/gmnfg6
Ambrazevičius, R., & Wiśniewska, I. (2008). Chromaticisms or Performance Rules? Evidence from Traditional Singing Pitch transcriptions. *Journal of Interdisciplinary Music Studies*, *2*(1/2), 19–31.
Andrews, H. K. (2001). H. In *Grove Music Online*. Oxford University Press. https://doi.org/10.1093/gmo/9781561592630.article.30242
Aristotle, & Mayhew, R. (2011). *Problems* (Vol. 1). Harvard University Press.
Arom, S. (2004). *African Polyphony and Polyrhythm: Musical Structure and Methodology* (M. Thom, B. Tuckett, & R. Boyd, Trans.). Cambridge University Press.
Arom, S. (2010). Outline of a syntax of chords in some songs from Samegrelo. In R. Tsurtsumia & J. Jordania (Eds.), *Proceedings: The Fifth International Symposium on Traditional Polyphony: 4–8 October, 2010, Tbilisi, Georgia* (pp. 266–277). Tbilisi State Conservatoire.
Arom, S., Fernando-Marandola, N., & Marandola, F. (2007). An Innovative Method for the Study of African Musical Scales: Cognitive and Technical Aspects. In C. Spyridis, A. Georgaki, G. Kouroupetroglou, & C. Anagnostopoulou (Eds.), *Proceedings of the 4th Sound and Music Computing Conference, Lefkada, Greece* (Issue July, pp. 107–116). University of Athens.
Asafyev, B. (1952). *Selected Works [Избранные труды]* (Vol. 1). Academy of Science of the USSR.
Bailey, R. (1986). *Richard Wagner: Prelude and Transfiguration from "Tristan und Isolde."* W. W. Norton & Company.
Baker, N. K. (1976). Heinrich Koch and the Theory of Melody. *Journal of Music Theory*, *20*(1), 1–48. https://doi.org/10/fh3wz7
Bakker, D. R., & Martin, F. H. (2015). Musical chords and emotion: Major and minor triads are processed for emotion. *Cognitive, Affective, & Behavioral Neuroscience*, *15*(1), 15–31. https://doi.org/10.3758/s13415-014-0309-4
Bakulina, E. (2014). The Concept of Mutability in Russian Theory. *Music Theory Online*, *20*(3). https://doi.org/10/gmnfh3
Banshchikov, G. (1997). *The rules of functional instrumentation [Законы функциональной инструментовки]*. Kompozitor.
Barbieri, P. (2003). Temperaments: Historical. In R. Palmieri & M. Palmieri (Eds.), *Piano: An Encyclopedia* (pp. 402–409). Routledge.
Barbour, J. M. (2004). *Tuning and Temperament: A Historical Survey*. Dover Publications.
Barker, A. (2007). *The Science of Harmonics in Classical Greece*. Cambridge University Press.
Beliaev, V. (1963). The Formation of folk modal systems. *Journal of the International Folk Music Council*, *15*, 4–9. https://doi.org/10/bmtgff
Beliayev, V. M. (1931). *The handbook for measuring folk musical instruments [Руководство для обмера народных музыкальных инструментов]*. State musical publishing [Госмузиздат].
Beliayev, V. M. (1971). *On musical folklore and ancient writing systems [О музыкальном фольклоре и древней письменности]*. Soviet Composer [Советский композитор].
Beliayev, V. M. (1990). Modal systems in the traditional music of the USSR [Ладовые системы в музыке народов СССР]. In I. Travkina (Ed.), *Viktor Mikhailovich Beliayev [Виктор Михайлович Беляев]* (pp. 223–377). Soviet Composer.
Benjamin, T., Horvit, M. M., & Nelson, R. (2015). *Techniques and materials of music: From the common practice period through the twentieth century*. Cengage Learning.
Benson, D. J. (2007). *Music: A mathematical offering*. Cambridge University Press.

Bent, M. (1984). Diatonic “Ficta.” *Early Music History*, *4*, 1–48. https://doi.org/10/bbk59k
Benward, B., & Saker, M. N. (2009). *Music in theory and practice* (Vol. 1). McGraw-Hill.
Berger, K. (2006). Concepts and developments in music theory. In J. Haar (Ed.), *European Music, 1520-1640* (pp. 304–328). Boydell Press.
Berndt, A., & Hähnel, T. (2010). Modeling musical dynamics. In K. Brandenburg (Ed.), *Proceedings of the 5th Audio Mostly Conference: A Conference on Interaction with Sound, Piteå, Sweden—September 15—17, 2010* (pp. 1–8). Fraunhofer Institute for Digital Media Technology. https://doi.org/10/bwxmn2
Berry, W. T. (1987). *Structural Functions in Music*. Dover Publications.
Bharucha, J. J. (1984). Anchoring effects in music: The resolution of dissonance. *Cognitive Psychology*, *16*(4), 485–518. https://doi.org/10/bw3bps
Bharucha, J. J. (1996). Melodic anchoring. *Music Perception*, *13*(3), 383–400. https://doi.org/10/gmnfg3
Bharucha, J. J. (2002). Neural nets, temporal composites, and tonality. In D. Levitin (Ed.), *Foundations of cognitive psychology: Core readings* (pp. 455–480). Bradford Books MIT Press.
Bobrovsky, V. P. (1976). Motif [Мотив]. In Y. V. Keldysh (Ed.), *Encyclopedia of Music [Музыкальная энциклопедия]* (Vol. 3, pp. 696–698). https://www.music-dic.ru/html-music-enc/m/5242.html
Bobrovsky, V. P. (1978). *The functional basics of musical form [Функциональные основы музыкальной формы]*. Muzyka.
Bolger, D., & Griffith, N. (2005). Multidimensional timbre analysis of shakuhachi honkyoku. In C. Traube & S. Lacasse (Eds.), *Proceedings of the Conference on Interdisciplinary Musicology (CIM05) Montréal (Québec) Canada, 10-12/03/2005* (pp. 10–12). Université Pierre et Marie Curie.
Borovik, T. (2006). *Teaching intervals in the ear-training lessons [Изучение интервалов на уроках сольфеджио]*. Klassika XXI.
Bowling, D. L., Gill, K. Z., Choi, J. D., Prinz, J., & Purves, D. (2010). Major and minor music compared to excited and subdued speech. *Journal of the Acoustical Society of America*, *127*(1), 491–503. https://doi.org/10/c42q3v
Brandl, R. (2008). New Considerations of Diaphony in Southeast Europe. In A. Ahmedaja & G. Haid (Eds.), *European Voices: Multipart singing in the Balkans and the Mediterranean* (Vol. 1, pp. 281–297). Böhlau Verlag.
Braudo, I. A. (1961). *Articulation. On pronunciation of melody [Артикуляция. О произношении мелодии.]*. State musical publishing.
Bregman, A. S. (1994). *Auditory Scene Analysis: The Perceptual Organization of Sound*. MIT Press.
Bregman, A. S., & McAdams, S. (1979). Hearing Musical Streams. *Computer Music Journal*, *3*(4), 26–43.
Brothers, T. D. (1997). *Chromatic Beauty in the Late Medieval Chanson: An Interpretation of Manuscript Accidentals*. Cambridge University Press.
Bukofzer, M. F. (1940). Popular Polyphony in the Middle Ages. *The Musical Quarterly*, *XXVI*(1), 31–49. https://doi.org/10/fnkbpq
Bukofzer, M. F. (2008). *Music in the Baroque Era—From Monteverdi to Bach*. Read Books.
Burnett, H. (1980). An Introduction to the History and Aesthetics of Japanese Jiuta-Tegotomono. *Asian Music*, *11*(2), 11–40. https://doi.org/10/ct2x94
Bytchkov, Y. N. (1987). *On systemic nature of modal organization in music [О системном характере ладовой организации в музыке]*. Gnessin Russian Academy of Music [Российская академия музыки имени Гнесиных].
Cambouropoulos, E. (2008). Voice And Stream: Perceptual And Computational Modeling Of Voice Separation. *Music Perception*, *26*(1), 75–94. https://doi.org/10/bmdsbr
Cambouropoulos, E. (2010). The Musical Surface: Challenging Basic Assumptions. *Musicae Scientiae*, *14*(2_suppl), 131–147. https://doi.org/10/gmnfvt
Caplin, W. E. (1998). *Classical form: A theory of formal functions for the instrumental music of Haydn, Mozart, and Beethoven*. Oxford University Press. https://global.oup.com/ushe/product/classical-

form-9780195143997?cc=us&lang=en&
Catoire, G. L. (2015). *The Theoretical course of harmony [Теоретический курс гармонии]*. USSR.
Cazden, N. (1958). Pythagoras and Aristoxenos Reconciled. *Journal of the American Musicological Society*, *11*(2/3), 97–105. https://doi.org/10/gmnfj5
Cazden, N. (1971). A Simplified Mode Classification for Traditional Anglo-American Song Tunes. In C. Haywood (Ed.), *Yearbook of the International Folk Music Council: Vol. Urbana, Il* (pp. 45–78). University of Illinois Press. https://doi.org/10.2307/767456
Cazden, N. (1980). The Definition of Consonance and Dissonance. *International Review of the Aesthetics and Sociology of Music*, *11*(2), 123–168. https://doi.org/10/ccqc4r
Chew, G. (2001). Articulation and phrasing. In S. Sadie & J. Tyrrell (Eds.), *The New Grove Dictionary of Music and Musicians*. 10.1093/gmo/9781561592630.article.08458. https://doi.org/10.1093/gmo/9781561592630.article.40952
Choron, A.-E., & Fayolle, F.-J.-M. (1971). *Dictionnaire Historique Des Musiciens*. Georg Olms.
Christesen, P., & Kyle, D. G. (2013). *A Companion to Sport and Spectacle in Greek and Roman Antiquity* (Vol. 8). John Wiley & Sons.
Clarke, E. F. (2001). Meaning and the specification of motion in music. *Musicae Scientiae*, *5*(2), 213–234. https://doi.org/10/gmnfg5
Clayton, M. R. L. (2000). *Time in Indian Music: Rhythm, Metre, and Form in North Indian Rag Performance*. Oxford University Press.
Clough, J. (1957). The Leading Tone in Direct Chromaticism: From Renaissance to Baroque. *Journal of Music Theory*, *1*(1), 2–21. https://doi.org/10/bfdq2w
Collier, W. G., & Hubbard, T. L. (2001). Musical scales and evaluations of happiness and awkwardness: Effects of pitch, direction, and scale mode. *American Journal of Psychology*, *114*(3), 355–375. https://doi.org/10/dvv7nw
Collier, W. G., & Hubbard, T. L. (2004). Musical scales and brightness evaluations: Effects of pitch, direction, and scale mode. *Musicae Scientiae*, *8*(2), 151–173. https://doi.org/10/gmnfhb
Cook, N. D. (2009). Harmony Perception: Harmoniousness is More Than the Sum of Interval Consonance. *Music Perception*, *27*(1), 25–42. https://doi.org/10.1525/mp.2009.27.1.25
Cooke, P. (2001). Heterophony. In S. Sadie & J. Tyrrell (Eds.), *The New Grove Dictionary of Music and Musicians*. Macmillan Publishers. https://doi.org/10.1093/gmo/9781561592630.article.12945
Costa, M., Ricci Bitti, P. E., & Bonfiglioli, L. (2000). Psychological Connotations of Harmonic Musical Intervals. *Psychology of Music*, *28*(1), 4–22. https://doi.org/10.1177/0305735600281002
Crickmore, L. (2014). The Ubiquity of the Diatonic Scale. *Proceedings of the ICONEA Conference "Arithmetical Subjectivism or Unconscious Knowledge", 10-12 Dec., 2014, Oxford*, 1–11.
Csapo, E. G., & Wilson, P. (2009). Timotheus the New Musician. In F. Budelmann (Ed.), *The Cambridge Companion to Greek Lyric* (pp. 277–293). Cambridge University Press.
Daniélou, A. (1995). *Music and the Power of Sound: The Influence of Tuning and Interval on Consciousness*. Inner Traditions.
Day-O'Connell, J. (2007). *Pentatonicism from the Eighteenth Century to Debussy*. University of Rochester Press.
Dean, R. T., Bailes, F., & Schubert, E. (2011). Acoustic Intensity Causes Perceived Changes in Arousal Levels in Music: An Experimental Investigation. *PLoS ONE*, *6*(4), e18591. https://doi.org/10/b82cdp
Dolzhansky, A. (1962). On the modal foundation of the compositions by Shostakovich [О ладовой основе сочинений Шостаковича]. In *The features of Shostakovich' style [Черты стиля Шостаковича]* (pp. 24–42).
Drabkin, W. (2001a). Diatonic. In *Grove Music Online* (Vol. 1). Oxford University Press. https://doi.org/10.1093/gmo/9781561592630.article.07727
Drabkin, W. (2001b). Motif. In *Grove Music Online*. Oxford University Press. https://doi.org/10.1093/gmo/9781561592630.article.19221
Drabkin, W. (2001c). Part. In *Grove Music Online*. Oxford University Press.

https://doi.org/10.1093/gmo/9781561592630.article.53861
Drabkin, W. (2001d). Register. In S. Sadie & J. Tyrrell (Eds.), *The New Grove Dictionary of Music and Musicians*. Macmillan Publishers. https://doi.org/10.1093/gmo/9781561592630.article.23072
Drabkin, W. (2001e). Theme. In S. Sadie & J. Tyrrell (Eds.), *The New Grove Dictionary of Music and Musicians*. Macmillan Publishers. https://doi.org/10.1093/gmo/9781561592630.article.27789
Drabkin, W. (2001f). Tonicization. In S. Sadie & J. Tyrrel (Eds.), *Grove Music Online*. Oxford University Press. https://doi.org/10.1093/gmo/9781561592630.article.28123
Dubovsky, I. I., Yevseyev, S. V., Sokolov, V. V., & Sposobin, I. V. (1965). *The textbook of harmony [Учебник гармонии]*. Muzyka.
Dyson, G., & Drabkin, W. (2001). Chromatic. In *Grove Music Online*. Oxford University Press. https://doi.org/10.1093/gmo/9781561592630.article.05718
Fabian, D. (2014). Commercial Sound Recordings and Trends in Expressive Music Performance. In D. Fabian, R. Timmers, & E. Schubert (Eds.), *Empirical approaches across styles and cultures* (pp. 58–79). Oxford University Press. https://www.academia.edu/24388496/Commercial_sound_recordings_and_trends_in_expressive_music_performance
Fabian, D., & Schubert, E. (2008). Musical Character and the Performance and Perception of Dotting, Articulation and Tempo in 34 Recordings of Variation 7 from J.S. Bach's Goldberg Variations (BWV 988). *Musicae Scientiae*, *12*(2), 177–206. https://doi.org/10/gmnfwb
Farhat, H. (2004). *The Dastgah Concept in Persian Music*. Cambridge University Press.
Farraj, J., & Shumays, S. A. (2019). *Inside Arabic Music: Arabic Maqam Performance and Theory in the 20th Century*. Oxford University Press.
Ferreira, M. P. (2015). Rhythmic paradigms in the *Cantigas de Santa Maria:* French versus Arabic precedent. *Plainsong and Medieval Music*, *24*(1), 1–24. https://doi.org/10.1017/S0961137115000017
Fétis, F.-J. (1994). *Esquisse de L'histoire de L'harmonie* (M. I. Arlin, Trans.). Pendragon Press.
Fortune, N., & Carter, T. (2001). Monody. In *Grove Music Online*. Oxford University Press. https://doi.org/10.1093/gmo/9781561592630.article.18977
Franklin, J. C. (2002). Diatonic Music in Greece: A Reassessment of Its Antiquity. *Mnemosyne*, *55*, 669–702. https://doi.org/10/d2vkvs
Franklin, J. C. (2005). Hearing Greek Microtones. *Ancient Greek Music in Performance. Vienna: Wiener Studien Beiheft*, *29*, 9–50.
Frobenius, W., Cooke, P., Bithell, C., & Zemtsovsky, I. (2001). Polyphony. In *Grove Music Online*. Oxford University Press. https://doi.org/10.1093/gmo/9781561592630.article.42927
Fuller, D. (2001). Accompaniment. In *Grove Music Online* (Vol. 1). Oxford University Press. https://doi.org/10.1093/gmo/9781561592630.article.00110
Garbuzov, N. (1950). *Zonal nature of tempo and rhythm [Зонная природа темпа и ритма]*. Academy of Science of the USSR.
Garbuzov, N. (1955). *Zonal nature of hearing of dynamics [Зонная природа динамического слуха]*. Gos Muz Izdat [State Musical Publishing].
Gauldin, R. (1983). The cycle-7 complex: Relations of diatonic set theory to the evolution of ancient tonal systems. *Music Theory Spectrum*, *5*(1), 39–55. https://doi.org/10/gmnfhf
Geiser, E., Ziegler, E., Jancke, L., & Meyer, M. (2009). Early electrophysiological correlates of meter and rhythm processing in music perception. *Cortex*, *45*(1), 93–102. https://doi.org/10.1016/j.cortex.2007.09.010
Gerardi, G. M., & Gerken, L. (1995). The development of affective responses to modality and melodic contour. *Music Perception*, *12*(3), 279–290. https://doi.org/10/gmnfjd
Gombosi, O. (1951). Key , Mode , Species. *Journal of the American Musicological Society*, *4*(1), 20–26.
Gurney, O. R. (1994). Babylonian Music Again. *Iraq*, *56*(1994), 101–106.
Haddon, E. (1952). Possible Origin of the Chopi Timbila Xylophone. *African Music Society Newsletter*, *1*(5), 61–67.

Hagel, S. (2009). *Ancient Greek Music: A New Technical History*. Cambridge University Press.
Halpern, A. R., Martin, J. S., & Reed, T. D. (2008). An ERP Study of Major-Minor Classification in Melodies. *Music Perception: An Interdisciplinary Journal*, *25*(3), 181–191. https://doi.org/10/dtv35v
Helmholtz, H. von. (1877). *On the Sensations of Tone as a Physiological Basis for the Theory of Music* (A. J. Ellis, Trans.). Longmans, Green and Co.
Henrich, N. (2006). Mirroring the voice from Garcia to the present day: Some insights into singing voice registers. *Logopedics Phoniatrics Vocology*, *31*(1), 3–14. https://doi.org/10/dqnh5z
Honing, H. (2002). Structure and interpretation of rhythm and timing. *Tijdschrift Voor Muziektheorie*, *7*(3), 227–232.
Honing, H., & Ladinig, O. (2009). Exposure influences expressive timing judgments in music. *Journal of Experimental Psychology: Human Perception and Performance*, *35*(1), 281–288. https://doi.org/10.1037/a0012732
Hornbostel, E. M. von. (1913). *Melody and Scale*. C.F. Peters.
Hubbard, T. L., & Ruppel, S. E. (2013). A Fröhlich effect and representational gravity in memory for auditory pitch. *Journal of Experimental Psychology.*, *39*(4), 1153–1164. https://doi.org/10/f46kw9
Huron, D. (1989). Characterizing Musical Textures. *Proceedings: 1989 International Computer Music Conference, November 2-5*, 131–134. http://hdl.handle.net/2027/spo.bbp2372.1989.033
Huron, D. (2001). Tone and Voice: A Derivation of the Rules of Voice-Leading from Perceptual Principles. *Music Perception*, *19*(1), 1–64. https://doi.org/10/c7xtfn
Huron, D. (2006). *Sweet Anticipation: Music and the Psychology of Expectation*. MIT Press.
Huron, D. (2008). A comparison of average pitch height and interval size in major and minor-key themes: Evidence consistent with affect-related pitch prosody. *Empirical Musicology Review*, *3*(2), 59–63. https://www.academia.edu/4478147/A_comparison_of_average_pitch_height_and_interval_size_in_major_and_minor_key_themes_Evidence_consistent_with_affect_related_pitch_prosody
Hutchinson, W., & Knopoff, L. (1978). The acoustic component of western consonance. *Interface*, *7*(1), 1–29. https://doi.org/10/bttf9c
Hyer, B. (2001a). Homophony. In S. Sadie & J. Tyrrell (Eds.), *The New Grove Dictionary of Music and Musicians*. Macmillan Publishers. https://doi.org/10.1093/gmo/9781561592630.article.13291
Hyer, B. (2001b). Key. In S. Sadie & J. Tyrrell (Eds.), *The New Grove Dictionary of Music and Musicians*. Macmillan Publishers. https://doi.org/10.1093/gmo/9781561592630.article.50818
Hyer, B. (2008). Tonality. In T. Christensen (Ed.), *The Cambridge History of Western Music Theory* (pp. 726–752). Cambridge University Press.
Hyer, B. (2012). What *is* a Function? In E. Gollin & A. Rehding (Eds.), *The Oxford Handbook of Neo-Riemannian Music Theories* (1st ed., pp. 92–139). Oxford University Press. https://doi.org/10.1093/oxfordhb/9780195321333.013.0003
Ivanchenko, G. V. (2001). *Psychology of music perception: Approaches, problems and prospects [Психология восприятия музыки: Подходы, проблемы, перспективы]*. Smysl.
Jerkert, J. (2003). *Measurements and models of musical articulation*. KTH Royal Institute of Technology.
Johnson, T. A. (2008). *Foundations of Diatonic Theory: A Mathematically Based Approach to Music Fundamentals*. Scarecrow Press.
Jones, M. R. (2016). Musical time. In S. Hallam, I. Cross, & M. Thaut (Eds.), *The Oxford Handbook of Music Psychology* (pp. 125–141). Oxford University Press.
Jordania, J. (2006). *Who Asked the First Question? The Origins of Human Choral Singing, Intelligence, Language and Speech*. Logos.
Jordania, J. (2011). *Why Do People Sing?: Music in Human Evolution*. Logos.
Kaeppler, A., Niles, D., Chenoweth, V., Love, J. W., & Zemp, H. (2013). Solomon Islands. In E. Koskoff (Ed.), *The Concise Garland Encyclopedia of World Music* (Vol. 9, pp. 682–688). Routledge.
Kalkun, A., & Oras, J. (2014). Seto Singing Tradition in Siberia: Songs and 'Non-Songs.' *Folklore*, *58*, 149–186. https://doi.org/10.7592/FEJF2014.58.kalkun

Kappraff, J. (2002). *Beyond Measure: A Guided Tour Through Nature, Myth, and Number*. World Scientific.
Keller, H. (1973). *Phrasing and articulation: A contribution to a rhetoric of music, with 152 musical examples.* W.W. Norton.
Kharlap, M. (1978). The Tactus System of Musical Rhythmics [Тактовая система музыкальной ритмики]. In V. Kholopova (Ed.), *The Problems of Musical Rhythm: Collection of Essays [Проблемы музыкального ритма: Сборник статей]* (pp. 48–104). Muzyka.
Kholopov, Y. (1973). Homophony [Гомофония]. In *Encyclopedia of Music [Музыкальная энциклопедия]* (Vol. 1, pp. 1047–1055). Soviet Encyclopedia [Советская энциклопедия].
Kholopov, Y. (1975). Modal harmony: Modality as a type of structure [Модальная гармония: Модальность как тип структуры]. In T. Solomonova (Ed.), *Art of music: General matters of theory and esthetics of music [Музыкальное искусство. Общие вопросы теории и эстетики музыки]* (pp. 16–31). Gafur Guliam [Издательство литературы и искусства имени Гафура Гуляма].
Kholopov, Y. (1988). *Harmony: A theoretic course [Гармония: Теоретический курс]*. Muzyka.
Kholopov, Y. (2006). *Musical-theoretic systems [Музыкально-теоретические системы]*. Kompozitor.
Kholopova, V. (1979). *Texture: An Essay [Фактура: Очерк]*. Muzyka.
Kholopova, V. (2002). *Theory of music: Melos, rhythm, texture, thematicism [Теория музыки: Мелодика, ритмика, фактура, те-матизм]*. Lan.
Kirigin, I. (2014). Some Theoretical Statements on the Art of Musical Folklore. *Journal of the International Folk Music Council*, *4*(1952), 54–56.
Knighton, T., & Fallows, D. (1997). *Companion to Medieval and Renaissance Music*. University of California Press.
Koço, E. (2015). *A Journey of the Vocal Iso(n)*. Cambridge Scholars Publishing.
Koelsch, S., Rohrmeier, M., Torrecuso, R., & Jentschke, S. (2013). Processing of hierarchical syntactic structure in music. *Proceedings of the National Academy of Sciences*, *110*(38), 15443–15448. https://doi.org/10/f5dpf7
Komar, A. J. (1971). *Theory of Suspensions: A Study of Metrical and Pitch Relations in Tonal Music*. Princeton University Press.
Kon, Y. (1973). Chord. In *Encyclopedia of Music [Музыкальная энциклопедия]* (Vol. 1, pp. 80–82). Soviet Encyclopedia [Советская энциклопедия].
Korsakova-Kreyn, M., & Dowling, W. J. (2014). Emotional processing in music: Study in affective responses to tonal modulation in controlled harmonic progressions and real music. *Psychomusicology: Music, Mind, and Brain*, *24*(1), 4–20. https://doi.org/10/gmnfhq
Korykhalova, M. P. (1973). Accompaniment [ Аккомпанемент]. In *Encyclopedia of Music [Музыкальная энциклопедия]* (Vol. 1, pp. 80–81). Soviet Encyclopedia [Советская энциклопедия].
Kreitner, K., Térey-Smith, M., Westrup, J., Holoman, D. K., Hopkins, G. W., Griffiths, P., & Conrad, J. A. (2001). Instrumentation and orchestration. In S. Sadie (Ed.), *The New Grove Dictionary of Music and Musicians*. Macmillan Publishers. https://doi.org/10.1093/gmo/9781561592630.article.20404
Kubik, G. (1999). *Africa and the Blues*. University Press of Mississippi.
Kubik, G. (2010). *Theory of African Music, Volume 1*. University of Chicago Press.
Kurth, E. (1931). *Musikpsychologie*. Max Hesses Verlag.
Kurth, E. (1991). *Ernst Kurth: Selected Writings* (L. A. Rothfarb, Ed.). Cambridge University Press. https://doi.org/10.1017/CBO9780511470288
Kushnaryov, C. (1958). *Matters of history and theory of Armenian monodic music [Вопросы истории и теории армянской монодической музыки]* (R. Atayan, Ed.). Gos Muz Izdat [State Musical Publishing].
Kvitka, K. V. (1971). *Selected Works [Избранные труды]* (V. L. Goshovsky, Ed.; Vol. 1). Soviet Composer.

Kvitka, K. V. (1973). *Selected Works [Избранные труды]* (V. L. Goshovsky, Ed.; Vol. 2). Soviet Composer.
Large, E. W., & Kolen, J. F. (1994). Resonance and the Perception of Musical Meter. *Connection Science*, *6*(2–3), 177–208. https://doi.org/10/b3mkrg
Large, E. W., & Snyder, J. S. (2009). Pulse and Meter as Neural Resonance. In S. Dalla Bella & V. B. Penhune (Eds.), *The Neurosciences and Music III : disorders and plasticity* (pp. 46–57). Published by Blackwell Pub. on behalf of the New York Academy of Sciences. https://doi.org/10.1111/j.1749-6632.2009.04550.x
Larson, S. (1997). The Problem of Prolongation in "Tonal" Music: Terminology, Perception, and Expressive Meaning. *Journal of Music Theory*, *41*, 101. https://doi.org/10/fp2r2t
Larson, S. (2012). *Musical Forces: Motion, Metaphor, and Meaning in Music*. Indiana University Press.
Larson, S., & McAdams, S. (2004). Musical forces and melodic expectations: Comparing computer models and experimental results. *Music Perception*, *21*(4), 457–498. https://doi.org/10/cgh69p
Larson, S., & Vanhandel, L. (2005). Measuring Musical Forces. *Music Perception*, *23*(2), 119–136. https://doi.org/10/ffw4zh
Lavrentyeva, I. V. (1981). Phrase [Фраза]. In Y. V. Keldysh (Ed.), *Encyclopedia of Music [Музыкальная энциклопедия]* (Vol. 5, pp. 918–922). Soviet Encyclopedia [Советская энциклопедия]. https://www.music-dic.ru/html-music-enc/f/8000.html
Leedy, D., & Haynes, B. (2001). Intonation. In S. Sadie & J. Tyrrell (Eds.), *The New Grove Dictionary of Music and Musicians*. Macmillan Publishers. https://doi.org/10.1093/gmo/9781561592630.article.53762
Lerdahl, F. (1987). Timbral hierarchies. *Contemporary Music Review*, *2*(1), 135–160. https://doi.org/10/bm74cc
Lerdahl, F. (2009). Genesis and Architecture of the GTTM Project. *Music Perception*, *26*(3), 187–194. https://doi.org/10/c6cd29
Lerdahl, F., & Jackendoff, R. S. (1985). *A Generative Theory of Tonal Music*. MIT Press.
Lerdahl, F., & Krumhansl, C. L. (2007). Modeling Tonal Tension. *Music Perception*, *24*(4), 329–366. https://doi.org/10/bw45tk
Lester, J. (1989). *Between Modes and Keys: German Theory, 1592-1802*. Pendragon Press.
Levitin, D. J. (1994). Absolute memory for musical pitch: Evidence from the production of learned melodies. *Perception & Psychophysics*, *56*(4), 414–423. https://doi.org/10/d82d3h
Lippman, E. A. (1964). *Musical Thought in Ancient Greece*. Da Capo Press.
Livingstone, S. R., Schubert, E., Loehr, J. D., & Palmer, C. (2009). Emotional arousal and the automatic detection of musical phrase boundaries. In A. Williamon, S. Pretty, & R. Buck (Eds.), *Proceedings of the International Symposium on Performance Science* (pp. 445–450). European Association of Conservatoires. https://doi.org/10.13140/RG.2.1.3957.9284
London, J. (2004). *Hearing in Time: Psychological Aspects of Musical Meter*. Oxford University Press.
Maceda, J. (1990). In search of a source of pentatonic hemitonic and anhemitonic scales in Southeast Asia. *Acta Musicologica*, *62*(2–3), 192–223. https://doi.org/10/ccf6cd
Malm, W. P. (2000). *Traditional Japanese Music and Musical Instruments* (Vol. 1). Kodansha International.
Mamcheva, N. A. (2012). *Musical instruments in Nivkh traditional culture [Музыкальные инструменты в традиционной культуре нивхов]*. GUP Sakhalinskaya Regional Press.
Maniates, M. R. (1993). Nicola Vicentino's reconstruction of the Ancient Greek Genera. *Revista de Musicología*, *16*(3), 16–36. https://doi.org/10/gmnf2q
Manuel, P. (1989). Modal Harmony in Andalusian, Eastern European, and Turkish Syncretic Musics. *Yearbook for Traditional Music*, *21*, 70–94. https://doi.org/10/bfmkpn
Margulis, E. H. (2005). A model of melodic expectation. *Music Perception*, *22*(4), 663–714. https://doi.org/10/bgbgbg
Marušić, D. (2007). Reception of Istrian musical traditions. *Музикологија: Часопис Музиколошког Института Српске Академије Наука и Уметности.*, *7*, 185–198.

Masata.k.a., N. (2006). Preference for consonance over dissonance by hearing newborns of deaf parents and of hearing parents. *Developmental Science*, *9*(1), 46–50. https://doi.org/10/cjcjd3
Mathiesen, T. J. (2001). Greece, §I, 6(iii): Ancient, Music theory: Aristoxenian tradition (e) Tonoi and harmoniai. In S. Sadie & J. Tyrrell (Eds.), *The New Grove Dictionary of Music and Musicians*. Macmillan Publishers. https://doi.org/10.1093/gmo/9781561592630.article.11694
Mathy, F., & Feldman, J. (2012). What's Magic about Magic Numbers? Chunking and Data Compression in Short-Term Memory. *Cognition*, *122*(3), 346–362. https://doi.org/10/ds3s2z
Mazel, L. (1952). *On melody [О мелодии]*. Gos Muz Izdat [State Musical Publishing].
Mazel, L. (1979). *Structuring of musical works [Строение музыкальных произведений]*. Muzyka.
Mazel, L. (1982). On certain aspects of Asafyev's concept [О некоторых сторонах концепции Б.В. Асафьева]. In I. Prudnikova (Ed.), *Essays on theory and analysis of music [Статьи по теории и анализу музыки]* (pp. 277–307). Soviet Composer.
Mazepus, V. V., & Galitskaya, S. P. (1997). *Musical culture of Siberia. Traditional culture of indigenous people of Siberia [Музыкальная культура Сибири. Традиционная культура коренных народов Сибири]* (B. A. Shindin, Ed.; Vol. 1). Novosibirsk State Conservatory named after Glinka.
McAdams, S., & Giordano, B. L. (2016). The Perception of Musical Timbre. In S. Hallam, I. Cross, & M. Thaut (Eds.), *The Oxford Handbook of Music Psychology* (pp. 113–124). Oxford University Press. https://doi.org/10.1093/oxfordhb/9780198722946.013.11
McDermott, J. H., Schultz, A. F., Undurraga, E. A., & Godoy, R. A. (2016). Indifference to dissonance in native Amazonians reveals cultural variation in music perception. *Nature*, *535*(7613), 547–550. https://doi.org/10/bmk3
McKinney, T. R. (2016). *Adrian Willaert and the Theory of Interval Affect: The Musica nova Madrigals and the Novel Theories of Zarlino and Vicentino*. Routledge. https://doi.org/10.4324/9781315565675
Messner, G. F. (1989). Jaap Kunst Revisited. Multipart singing in three East Florinese villages fifty years later: A preliminary investigation. *The World of Music*, *31*(2), 3–51. https://doi.org/10.2307/43561214
Meyer, J. (2009). *Acoustics and the performance of music manual for acousticians, audio engineers, musicians, architects and musical instruments makers* (U. Hansen, Trans.). Springer.
Miller, G. A. (1994). The magical number seven, plus or minus two: Some limits on our capacity for processing information. *Psychological Review*, *101*(2), 343–352. https://doi.org/10/d66f6q
Miller, R. J. (2014). *Contemporary Orchestration: A Practical Guide to Instruments, Ensembles, and Musicians*. Routledge. https://doi.org/10.4324/9781315815008-11
Nan, Y., Knösche, T. R., & Friederici, A. D. (2006). The perception of musical phrase structure: A cross-cultural ERP study. *Brain Research*, *1094*(1), 179–191. https://doi.org/10/d739p2
Nan, Y., Knösche, T. R., & Friederici, A. D. (2009). Non-musicians' perception of phrase boundaries in music: A cross-cultural ERP study. *Biological Psychology*, *82*(1), 70–81. https://doi.org/10/c7nc45
Nattiez, J.-J. (1983). The rekkukara of the Ainu (Japan) and the katajjaq of the Inuit (Canada): A comparison. *The World of Music*, *25*(2), 33–44. https://doi.org/10.2307/43560906
Nazaikinsky, Y. V. (1972). *On psychology of human musical perception [О психологии музыкального восприятия]*. Muzyka.
Nazaikinsky, Y. V. (1977). Interconnection between the intervallic-based and degree-based representation of music in the development of a musical ear [Взаимосвязи интервальных и ступеневых представлений в развитии музыкального слуха]. In A. Agazhanov (Ed.), *Development of Musical Hearing [Воспитание музыкального слуха]* (Vol. 1, pp. 25–77). Muzyka.
Nazaikinsky, Y. V. (1982). *The logic of musical composition [Логика музыкальной композиции]*. Muzyka.
Nazaikinsky, Y. V. (2013). *Style and genre in music [Стиль и жанр в музыке]*. Tbilisi State Conservatoire.

Nazaikinsky, Y. V., & Rags, Y. N. (1964). Perception of musical timbres and the significance of the individual harmonics in a sound [Восприятие музыкальных тембров и значение отдельных гармоник звука]. In S. S. Skrebkov (Ed.), *Application of the acoustic methods in musicology [Применение акустических методов в музыкознании]* (pp. 79–100). Muzyka.
Neuhaus, C., Knösche, T. R., & Friederici, A. D. (2006). Effects of musical expertise and boundary markers on phrase perception in music. *Journal of Cognitive Neuroscience*, *18*(3), 472–493. https://doi.org/10.1162/089892906775990642
Nikolsky, A. (2015a). Evolution of tonal organization in music mirrors symbolic representation of perceptual reality. Part-1: Prehistoric. *Frontiers in Psychology*, *6*(1405). https://doi.org/10/f7wvp8
Nikolsky, A. (2015b). Tonal Organization in Tuning of Paleolithic and Neolithic Pipes. *Frontiers in Psychology*, *6*, 14. https://doi.org/10.6084/m9.figshare.13636313
Nikolsky, A. (2016a). Can folk music be chromatic?: "Natural" microtonal modes, chromatic degrees and alterations. *Frontiers in Psychology*, *7*, 18. https://doi.org/10/gmnfzf
Nikolsky, A. (2016b). Chromatic alteration as expression of aesthetic emotion: From the Ancient doctrine of ethos to the emergence of the notion of musical error. *Frontiers in Psychology*, *7*. https://doi.org/10/gmnfz3
Nikolsky, A. (2016c). Chromaticism in the modern hemiolic mode of the Syrian Orthodox chant: The heritage of the Ancient Greek music. *Frontiers in Psychology*, *7*. https://doi.org/10/gmnfz4
Nikolsky, A. (2016d). Evolution of Tonal Organization in Music Optimizes Neural Mechanisms in Symbolic Encoding of Perceptual Reality. Part-2: Ancient to Seventeenth Century. *Frontiers in Psychology*, *7*, 211. https://doi.org/10/gmdd4n
Nikolsky, A. (2016e). Hemiolic musical mode as post-Hellenistic development of the Ancient Greek chromatic system. *Frontiers in Psychology*, *7*. https://doi.org/10/gmnfz5
Nikolsky, A. (2016f). Non-octave Hypermode as a special form of tonal organization of Orthodox Christian plainsong. *Frontiers in Psychology*, *7*. https://doi.org/10/gmnfz7
Nikolsky, A. (2016g). The commonalities between melodic line, geometric line, and environmental topography in traditional cultures of Northern Siberia: "Landscape aesthetics" as a model of musical genesis. *Frontiers in Psychology*, *7*. https://doi.org/10/gmnfz2
Nikolsky, A. (2016h). The Correspondence Between Composition in Fine Arts and Music throughout History. In *Frontiers in Psychology* (Vol. 7). https://doi.org/10.6084/m9.figshare.15081477
Nikolsky, A. (2017). *A Handbook of Structural Analysis of Music for Systematic Musicology* (p. 136). Synergo Music. https://doi.org/10.6084/m9.figshare.13627838.v2
Nikolsky, A. (2018). General typology of music texture in the evolutionary earliest forms of music. Commentary on "The 'Musilanguage' Model of Language Evolution." *Frontiers in Psychology*, *9*, 75. https://doi.org/10/gmnfrt
Nikolsky, A. (2020). A new method of modal multifactorial analysis of tonal organization in music. *Frontiers in Psychology*, *11*. https://doi.org/10/gmnfzv
Nikolsky, A., Alekseyev, E. Ye., Alekseev, I. Ye., & Dyakonova, V. E. (2017). Prolegomena of modal organization of Jaw harp music on the example of the articulatory degrees of "talking khomus" [Пролегомена ладовой организации варганной музыки на примере использования артикуляционных ступеней в "говорящем" якутском хомусе]. In O. V. Novikova (Ed.), *Systemic methods of the research on musical culture, International scientific practical conference in memory of V. V. Mazepus [Системные методы изучения музыкальной культуры, Международная научно-практическая конференция памяти В.В.Мазепуса, 31/X-1/XI 201*. Novosibirsk State Conservatory named after Glinka. https://doi.org/10/gmnfvc
Nikolsky, A., & Benítez-Burraco, A. (2022). *Human aggression and music evolution: A model.* PsyArXiv. https://doi.org/10.31234/osf.io/a8up7
Noorden, L. van. (1975). *Temporal Coherence in the Perception of Tone Sequences* (Vol. 3). Institute for Perceptual Research.
Oelmann, H., & Laeng, B. (2009). The emotional meaning of harmonic intervals. *Cognitive Processing*,

*10*(2), 113–131. https://doi.org/10.1007/s10339-008-0217-z
Ogolevets, A. (1941). *The Basics of Harmonic Language [Основы гармонического языка]*. Muzgiz.
Osgood, C. E., Suci, G. J., & Tannenbaum, P. H. (1957). *The measurement of meaning*. University of Illinois Press.
Owens, J. A. (1998). *Composers at Work: The Craft of Musical Composition 1450-1600*. Oxford University Press.
Pacholczyk, J. (1993). Early Arab Suite in Spain: An Investigation of the Past Through the Contemporary Living Traditions. *Revista de Musicología*, *16*(1), 358–366. https://doi.org/10/gmnf3g
Palisca, C. V. (1960). Vincenzo Galilei and Some Links between “Pseudo-Monody” and Monody. *The Musical Quarterly*, *46*(3), 344–360. https://doi.org/10/d9ns8s
Paraskeva, S., & McAdams, S. (1997). Influence of timbre, presence/absence of tonal hierarchy and musical training on the perception of musical tension and relaxation schemas. *Proceedings of the International Computer Music Conference, Thessaloniki, Greece, September 25-30, 1997*, 438–441.
Parncutt, R. (1989). *Harmony: A psychoacoustical approach* (pp. xii, 206). Springer-Verlag Publishing. https://doi.org/10.1007/978-3-642-74831-8
Pashinian, E. (1973). Universal super-modal system in Armenian music [Универсальная суперладовая система в армянской музыке]. *Historical Philological Journal of the National Academy of Sciences of Armenia*, *3*, 194–212.
Patterson, R. D., Gaudrain, E., & Walters, T. C. (2010). The Perception of Family and Register in Musical Tones. In Riess Jones M., R. Fay, & A. Popper (Eds.), *Springer Handbook of Auditory Research* (pp. 13–50). Springer. https://doi.org/10.1007/978-1-4419-6114-3_2
Plack, C. J., & Watkinson, R. K. (2010). Perceived continuity and pitch shifts for complex tones with unresolved harmonics. *The Journal of the Acoustical Society of America*, *128*(4), 1922–1929. https://doi.org/10/bd8v8h
Powers, H. S. (2001). Final. In S. Sadie & J. Tyrrel (Eds.), *The New Grove Dictionary of Music and Musicians*. Macmillan Publishers.
Powers, H. S., Wiering, F., Porter, J., & Cowdery, J. (2001). Mode. The term. Medieval modal theory. Modal theories and polyphonic music. Modal scales and traditional music. In S. Sadie & J. Tyrrell (Eds.), *The New Grove Dictionary of Music and Musicians*. Macmillan Publishers. https://doi.org/10.1093/gmo/9781561592630.article.43718
Protopopov, V. V. (1978). Polyphony. In Y. V. Keldysh (Ed.), *Encyclopedia of Music [Музыкальная энциклопедия]* (Vol. 4, pp. 344–364). Soviet Encyclopedia [Советская энциклопедия].
Quinn, I., & Mavromatis, P. (2011). Voice-Leading Prototypes and Harmonic Function in Two Chorale Corpora. In C. Agon, M. Andreatta, G. Assayag, E. Amiot, J. Bresson, & J. Mandereau (Eds.), *Mathematics and Computation in Music* (Vol. 6726, pp. 230–240). Springer Berlin Heidelberg.
Rags, Y. N. (1980). The concept of zonal nature of musical hearing by N.A. Garbuzov [Концепция зонной природы музыкального слуха Н.А. Гарбузова]. In Y. Rags (Ed.), *Garbuzov N.A.—Musician, researcher and pedagoge [Гарбузов Н.А.—Музыкант, исследователь, педагог]* (pp. 11–48). Muzyka.
Raman, R., & Dowling, W. J. (2012). Analyzing Modulation in Scales (Rāgams) in South Indian Classical (Carnātic) Music: A Behavioral Study. In E. Cambouropoulos, C. Tsougras, P. Mavromatis, & K. Pastiadis (Eds.), *Proceedings of the 12th International Conference on Music Perception and Cognition (ICMPC)* (pp. 837–838). Aristotle University of Thessaloniki.
Rameau, J.-P. (1971). *Treatise on Harmony [Traité de l’harmonie réduite à ses principes naturels]* (P. Gossett, Trans.). Dover Publications.
Ramos, D., Bueno, J. L. O., & Bigand, E. (2011). Manipulating Greek musical modes and tempo affects perceived musical emotion in musicians and nonmusicians. *Brazilian Journal of Medical and Biological Research*, *44*(2), 165–172. https://doi.org/10/ckt3gq
Ratner, L. G. (2001). Period. In S. Sadie & J. Tyrrell (Eds.), *The New Grove Dictionary of Music and Musicians*. Macmillan Publishers. https://doi.org/10.1093/gmo/9781561592630.article.21337

Repp, B. H. (1990). Patterns of expressive timing in performances of a Beethoven minuet by nineteen famous pianists. *The Journal of the Acoustical Society of America*, *88*(2), 622–641. https://doi.org/10.1121/1.399766

Repp, B. H. (1995). Quantitative Effects of Global Tempo on Expressive Timing in Music Performance: Some Perceptual Evidence. *Music Perception*, *13*(1), 39–57. https://doi.org/10.2307/40285684

Repp, B. H. (1998). Obligatory "expectations" of expressive timing induced by perception of musical structure. *Psychological Research*, *61*(1), 33–43. https://doi.org/10.1007/s004260050011

Réti, R. (1951). The Thematic Process in Music. In *London*. Macmillan Publishers.

Rîpă, C. (2010). Genesis of the Chords. *Recent Advances in Acoustics & Music*, *25*, 161–165.

Rockstro, W. S., Dyson, G., Drabkin, W., Powers, H. S., & Rushton, J. (2001). *Cadence*. Oxford University Press. https://doi.org/10.1093/gmo/9781561592630.article.04523

Roesner, E. H. (2001). Rhythmic modes (Modal Rhythm). In S. Sadie & J. Tyrrell (Eds.), *The New Grove Dictionary of Music and Musicians*. Macmillan Publishers. https://doi.org/10.1093/gmo/9781561592630.article.23337

Rohrmeier, M. (2011). Towards a generative syntax of tonal harmony. *Journal of Mathematics and Music*, *5*(1), 35–53. https://doi.org/10.1080/17459737.2011.573676

Rohrmeier, M., Dienes, Z., Guo, X., & Fu, Q. (2014). Implicit Learning and Recursion. In F. Lowenthal & L. Lefebvre (Eds.), *Language and Recursion* (pp. 67–85). Springer New York. https://doi.org/10.1007/978-1-4614-9414-0_6

Rothfarb, L. A. (1979). *Ernst Kurth's The Requirements for a Theory of Harmony: An annotated translation with an introductory essay*. University of Hartford.

Rothfarb, L. A. (1988). *Ernst Kurth as theorist and analyst*. University of Pennsylvania Press.

Rubtsov, F. (1964). *The foundations of modal morphology of Russian traditional songs [Основы ладового строения русских народных песен]*. Muzyka.

Rudneva, A. (1994). *Russian traditional musical works: Essays on the theory of folklore [Русское народное музыкальное творчество: Очерки по теории фольклора]*. Kompozitor.

Sa.k.a.ta, H. L., McAdams, S., & Nosulenko, V. (1996). Systematic Analysis of Verbalizations Produced in Comparing Musical Timbres. *International Journal of Psychology*, *31*(6), 255–278. https://doi.org/10/cnm9vv

Sandell, G. J. (1995). Roles for Spectral Centroid and Other Factors in Determining "Blended" Instrument Pairings in Orchestration. *Music Perception: An Interdisciplinary Journal*, *13*(2), 209–246. https://doi.org/10/gmnftt

Schidlovsky, N. (2009). Sources of Russian chant theory. In G. D. McQuere (Ed.), *Russian theoretical thought in music* (pp. 83–108). University of Rochester Press.

Schneider, A. (2013). Change and continuity in sound analysis: A review of concepts in regard to musical acoustics, music perception, and transcription. In R. Bader (Ed.), *Sound—Perception—Performance* (pp. 71–111). Springer. https://doi.org/10.1007/978-3-319-00107-4_3

Seeger, A. (2004). *Why Suyá Sing: A Musical Anthropology of an Amazonian People*. University of Illinois Press.

Sheikin, Y. I. (1996). *Musical culture of peoples of Northern Asia [Музыкальная культура народов Северной Азии]*. Yakutskii scientific center.

Sheikin, Y. I. (2002). *The history of music culture of Siberian ethnicities: A comparative historical investigation [История музыкальной культуры народов Сибири: Сравнительно-историческое исследование]*. Eastern Literature, Russian Academy of Science.

Sheikin, Y. I. (2018). *Musical culture of Chukchi [Музыкальная культура чукчей]* (O. Dobzhanskaya & T. Ignatyeva, Eds.). Nauka.

Shepard, R. N. (2010). One cognitive psychologist's quest for the structural grounds of music cognition. *Empirical Musicology Review*, *20*(1–2), 130–157. https://doi.org/10.5084/pmmb2009/20/130

Shestakov, V. P. (1975). *From Ethos to Affect. History of musical aesthetics from Antiquity to the 18th century [От Этоса к Аффекту. История музыкальной эстетики от античности до XVIII века]*. Muzyka.

Skrebkova-Filatova, M. S. (1985). *Texture in music [Фактура в музыке]*. Muzyka.
Smith, L. D., & Williams, R. N. (1999). Children's artistic responses to musical intervals. *The American Journal of Psychology*, *112*(3), 383–410.
Spiess, L. B. (1957). An introduction to the pre-history of polyphony. In *Essays on Music in Honor of Archibald Thomson Davison by his associates* (pp. 11–15). Harvard University.
Spitzer, J., & Zaslaw, N. (2004). *The Birth of the Orchestra: History of an Institution, 1650-1815*. OUP Oxford.
Sposobin, I. V. (1969). *Lectures on the course of harmony [Лекции по курсу гармонии]* (Y. Kholopov, Ed.). Muzyka.
Stamou, L. (2002). Plato and Aristotle On Music and Music Education: Lessons From Ancient Greece. *International Journal of Music Education*, *39*(1), 3–16. https://doi.org/10/c3v9nd
Starostina, T. (1973). Modal systematization of Russian traditional song [Ладовая систематика русской народной песни]. In *Harmony: Problems of science and methodology [Гармония: Проблемы науки и методики]* (Vol. 1, pp. 85–105). Muzyka.
Stefanics, G., Háden, G. P., Sziller, I., Balázs, L., Beke, A., & Winkler, I. (2009). Newborn infants process pitch intervals. *Clinical Neurophysiology*, *120*(2), 304–308. https://doi.org/10/cfxvqw
Straehley, I. C., & Loebach, J. L. (2014). The influence of mode and musical experience on the attribution of emotions to melodic sequences. *Psychomusicology: Music, Mind, and Brain*, *24*(1), 21–34. https://doi.org/10/gmnfq6
Stumpf, C. (1897). Die pseudo-aristotelischen Probleme über Musik. In *Abhandlungen der Königlichen A.k.a.demie der Wissenschaften zu Berlin* (Vol. 3). A.k.a.demie der Wissenschaften. http://echo.mpiwg-berlin.mpg.de/ECHOdocuView?url=/permanent/vlp/lit8473/index.meta
Stumpf, C. (1901). Tonsystem und Musik der Siamesen. In C. Stumpf (Ed.), *Beiträge zur Akustik und Musikwissenschaft* (Vol. 3, pp. 69–138). Veerlag von Johann Ambrosius Barth.
Stumpf, C. (1911). Konsonanz und Konkordanz. Nebst Bemerkungen über Psychologie und Wohlgefälligkeit musikalischer Zusammenklänge. *Zeitschrift Für Psychologie Und Physiologie Der Sinnesorgane*, *58*, 321-355.
Sundberg, J. (1987). *The Science of the Singing Voice*. Northern Illinois University Press.
Sussman, E. (2005). Integration and segregation in auditory scene analysis. *The Journal of the Acoustical Society of America*, *117*(3), 1285. https://doi.org/10/b9zbkm
Sussman, E., Horváth, J., Winkler, I., & Orr, M. (2007). The role of attention in the formation of auditory streams. *Attention, Perception, and Psychophysics*, *69*(1), 136–152. https://doi.org/10/fvttmd
Swain, J. P. (2002). *Harmonic Rhythm: Analysis and Interpretation*. Oxford University Press.
Swan, A. J. (1943). The nature of the Russian folk-song. *Musical Quarterly*, *29*(4), 498–516. https://doi.org/10/cf9mp6
Tagg, P. (2003). Modality. In J. Shepherd, D. Horn, D. Laing, P. Oliver, & P. Wicke (Eds.), *Continuum Encyclopedia of Popular Music of the World Part 1 Performance and Production* (Vol. 2, p. 712). Continuum.
Taruskin, R. (1985). Chernomor to Kashchei: Harmonic Sorcery; Or, Stravinsky's "Angle." *Journal of the American Musicological Society*, *38*(1), 72–142. https://doi.org/10.2307/831550
Terhardt, E. (1974). Pitch, consonance, and harmony. *The Journal of the Acoustical Society of America*, *55*(5), 1061–1069. https://doi.org/10/fks3b7
Thiemel, M. (2001). Dynamics. In S. Sadie & J. Tyrrell (Eds.), *The New Grove Dictionary of Music and Musicians*. Macmillan Publishers. https://doi.org/10.1093/gmo/9781561592630.article.08458
Titze, I. R. (1988). A framework for the study of vocal registers. *Journal of Voice*, *2*(3), 183–194. https://doi.org/10/dz2428
Titze, I. R. (2000). *Principles of voice production*. National Center for Voice and Speech.
Tiulin, Y. N. (1937). *The doctrine of harmony [Учение о гармонии]*. Muzyka.
Tiulin, Y. N. (1969). *The construction of musical speech [Строение музыкальной речи]* (2nd ed.). Muzyka.
Todd, N. (1985). A Model of Expressive Timing in Tonal Music. *Music Perception*, *3*(1), 33–57.

https://doi.org/10.2307/40285321
Tomlinson, G. (1981). Madrigal, Monody, and Monteverdi's "Via Naturale Alla Immitatione." *Journal of the American Musicological Society*, *34*(1), 60–108. https://doi.org/10.2307/831035
Touma, H. H. (1996). *The Music of the Arabs*. Amadeus Press.
Tramo, M., Cariani, P., Delgutte, B., & Braida, L. D. (2001). Neurobiological foundations for the theory of harmony in western tonal music. *Annals of the New York Academy of Sciences*, *930*(1), 92–116. https://doi.org/10/cb5g5d
Trehub, S. E., Cohen, A. J., Thorpe, L. A., & Morrongiello, B. A. (1986). Development of the perception of musical relations: Semitone and diatonic structure. *Journal of Experimental Psychology. Human Perception and Performance*, *12*(3), 295–301. https://doi.org/10/b7h3tt
Trochidis, K., & Bigand, E. (2013). Investigation of the Effect of Mode and Tempo on Emotional Responses to Music Using EEG Power Asymmetry. *Journal of Psychophysiology*, *27*(3), 142–148. https://doi.org/10/gmnfq5
Tull, J. R., & Asafyev, B. (2000). *B.V. Asaf'ev's Musical form as a process: Translation and commentary: Vol. 3 volumes* (J. R. Tull, Trans.). University Microfilms International [Publisher]. https://doi.org/610363518
Val'kova, V. B. (1992). *Musical thematicism, cognition, culture [Музыкальный тематизм, мышление, культура]*. State Nizhegorodskii University.
Vassilakis, P. N. (2005). Auditory roughness as means of musical expression. *Selected Reports in Ethnomusicology*, *12*(Selected Reports in Ethnomusicology), 119–144.
Vega, D. (2003). A perceptual experiment on harmonic tension and melodic attraction in Lerdahl's Tonal Pitch Space. *Musicae Scientiae*, *7*(1), 35–55. https://doi.org/10/gmnfg2
Virtala, P., & Tervaniemi, M. (2017). Neurocognition of Major-Minor and Consonance-Dissonance. *Music Perception*, *34*(4), 387–404. https://doi.org/10.1525/mp.2017.34.4.387
Volodin, A. A. (1970). The role of harmonic spectrum in perception of pitch and timbre [Роль гармонического спектра в восприятии высоты и тембра звука]. In *Musical art and science [Музыкальное искусство и наука]* (Vol. 1, pp. 11–38). Muzyka.
Volodin, A. A. (1972). *Psychological aspects of perception of music [Психологические аспекты восприятия музыки]*. The Institute of Evolutionary Physiology and Biochemistry named after Sechenov.
von Bismarck, G. (1974). Timbre of steady sounds: A factorial investigation of its verbal attributes. *Acustica*, *30*(3), 146–159.
Werner, E. (1948). The origin of the eight modes of music (Octoechos). A study in musical symbolism. *Hebrew Union College Annual*, *21*, 211–255.
West, M. L. (1981). The Singing of Homer and the Modes of Early Greek Music. *The Journal of Hellenic Studies*, *101*, 113–129. https://doi.org/10/dmwpnn
West, M. L. (1992). *Ancient Greek music*. Oxford University Press.
Wienpahl, R. W. (1972). Modality, monality and tonality in the sixteenth and seventeenth centuries: II. *Music & Letters*, *53*(1), 59–73. https://doi.org/10/bbxb8s
Wiora, W. (1959). Older than Pentatony. In B. Rejeczky (Ed.), *Studia Memoriae Bela Bartok Sacra* (pp. 183–206). Boosey and Hawkes.
Woolhouse, M., Cross, I., & Horton, T. (2016). Perception of nonadjacent tonic-key relationships. *Psychology of Music*, *44*(4), 802–815. https://doi.org/10.1177/0305735615593409
Yavorskii, B. (1908). *The construction of musical speech. Data and notes [Строение музыкальной речи. Материалы и заметки]* (Vol. 1). Aralov, G.
Yemelyanov, V. (2000). *Voice development: Coordination and training [Развитие голоса: Координация и тренаж]* (Yu. Sandulov, Ed.). Lan.
Yesipova, M. V. (2008). Phono-instruments (Ethnophones). In M. V. Yesipova (Ed.), *Musical Instruments [Музыкальные инструменты]* (p. 633). Deka-VS.
Zacharakis, A., Pastiadis, K., & Reiss, J. D. (2014). An Interlanguage Study of Musical Timbre Semantic Dimensions and Their Acoustic Correlates. *Music Perception: An Interdisciplinary Journal*,

*31*(4), 339–358. https://doi.org/10/ggs8mh
Zagretdinov, R. A. (1997). *The school of playing kubyz: A practical methodological aid [Школа игры на кубызе: Учебно-Методическое Пособие]* (T. Z. M. Alkin, Ed.). Belaya Reka.
Zemtsovsky, I. (1972). *Slavic musical folklore: Articles and materials [Славянский музыкальный фольклор: Статьи и материалы]*. Muzyka.
Zemtsovsky, I. (1974). Semasiology of Musical Folklore (Methodological Premises) [Семасиология музыкального фольклора (методологические предпосылки)]. In M. Aranovsky (Ed.), *Problems of Musical Thinking [Проблемы музыкального мышления]* (pp. 177–207). Muzyka.
Zemtsovsky, I. (1980). Asafyev and methodological foundations of intonational analysis of the folk music [Б.В.Асафьев и методологические основы интонационного анализа народной музыки]. In O. P. Kolovskii (Ed.), *Criticism and musicology [Критика и музыкознание]* (Vol. 2, pp. 184–198). Muzyka.
Zemtsovsky, I. (2012). Again on the origin of music [Снова о происхождении музыки]. In A. F. Nekrylova (Ed.), *Zelenyi Zal [Зеленый зал: Альманах РИИИ]* (Vol. 3, pp. 7–27). The Russian Institute of History of Arts.
